# Anti-cancer activity elucidation of geissolosimine as an MDM2-p53 interaction inhibitor: An *in-silico* study

**DOI:** 10.1371/journal.pone.0323003

**Published:** 2025-05-08

**Authors:** Md. Al-Amin, Rehnuma Tanjin, Md. Rasul Karim, Jannatul Mawa Etee, Ayesha Siddika, Nafisa Akter, Md. Helal Uddin, Ratul Mahmud, Tasfia Saffat, Md. Faruk Hossen, Samira Idris Mowlee, Elmu Kabir Rafa, Sumi Akter

**Affiliations:** Department of Pharmacy, Islamic University, Kushtia, Bangladesh; Nnamdi Azikiwe University, NIGERIA

## Abstract

For cancer treatment, Inhibition of murine double minute (MDM2) & p53 interaction is considered an attractive therapeutic approach. In this study, we performed an integrated virtual screening (*i.e., QSAR,* structural similarity, molecular docking, and molecular dynamic simulation) on the in-house building alkaloids library. Geissolosimine (*i.e.,* an indole alkaloid) was predicted as a potential inhibitor for MDM2-p53 interaction. The predicted pIC50 value of Geissolosimine, was 7.013 M. Moreover, Geissolosimine showed 0.62% structural similarity to ‘SAR405838’ (*i.e.,* a clinical trial inhibitor for MDM2-p53 interaction inhibition); and a docking score of -10.9 kcal/mol that was higher than the ‘SAR405838’.100 ns molecular dynamics simulation (MDS) was performed to validate the docking result and it exhibited better binding stability to MDM2. The pharmacokinetic & drug-likeness analysis suggested that Geissolosimine had potential to be a drug-like compound. However, in vitro & in vivo assays will be required to validate this study.

## 1. Introduction

In a normal cellular state, p53 (*i.e.,* tumor suppressor) causes cell cycle arrest in the G1 phase in response to DNA damage [1]. However, in approximately 50% of human cancers, p53 is mutated. In the other 50% of cancers, it retains wild-type activity but its tumor suppressor activity is significantly inhibited by an endogenous ubiquitin protein called murine double minute 2 (MDM2) [2].

MDM2 downregulates p53 activity through the several mechanisms such as (a) It binds with the transactivation domain of p53 to inhibit transactivation activity, (b) it exports p53 out of the nucleus & exerts ubiquitination ligase activity by proteasomes [3,4]. However, overexpression & frequent activation of MDM2 are observed in many human cancers including hepatocellular carcinoma [5]. Therefore, inhibition of MDM2-p53 interaction can restore the tumor suppressor activity of p53 and this strategy emerges as a novel therapeutic approach for cancer treatment [6]. Conventional chemotherapeutic agents cause DNA damage which can lead to a secondary malignancy in some cases. On the other hand, MDM2 inhibitors do not cause DNA damage directly [3].

Up to date, a handful of MDM2-p53 interaction inhibitors are undergoing clinical trial, for example RG7112, idasanutlin, SAR405838, milademetan, APG-115, AMG 232, NVP-CGM097, siremadlin, MK-8242 [7]. It seems that the MDM2-p53 interaction inhibitors discovery pipeline is not sufficient. Hence, discovering new inhibitors for MDM2-p53 interaction is an urgent need.

In the last 50 years of the 20^th^ century, approved ~50% of anti-tumor agents were directly derived from natural sources or semi-synthetic analogues of natural source-based compounds. Because phyto-compounds have less toxic properties than synthetic compounds and also possess greater stability, longer-lasting target effects, *etc.* [8]. For instance, several alkaloids were approved for the cancer treatment such as homoharringtonine (*i.e.,* approved in 2012 for myeloid leukemia treatment), vincristine, vinblastine, paclitaxel (PTX), and camptothecin [9,10].

Quantitative structure-activity relationship (QSAR) quantitatively relates structural descriptors of compounds to biological activities. The principle beyond QSAR is that the structural information of compounds is encoded into various descriptors such as constitutional, topological, thermodynamic, steric, electronics*, etc.* to construct a mathematical relationship between the descriptors and biological activities [11,12].

Structurally similar compounds may have similar physio-chemical and biological activities [13]. For similarity searching, various 1D, 2D and 3D fingerprints are used such as extended connectivity fingerprint (ECFP), MACCS, *etc* [13].

Several studies conducted to identify potential inhibitors of MDM2-p53 interaction such as Ghafoor *et al*. performed drug repurposing of FDA-approved drugs and suggested two antihistamine drugs: cetirizine and rupatadine as potential repurposing candidates [14]. Moreover, Sirous *et al.* applied structural similarity, molecular docking & simulation-based approach to screening the PubChem database & found some compounds as MDM2-p53 interaction inhibitors [15]. Here, solely screening alkaloids as MDM2-p53 interaction inhibitors by implementing an integrated QSAR, structural similarity searchinng, molecular docking & simulation-based approach is a distinct point of our study. This study aims to explore the alkaloids library to elucidate their potential as MDM2-p53 interaction inhibitors.

## 2. Materials and methods

### 2.1 Alkaloid enlistment

FDA-approved 60% of antineoplastic drugs are connected to natural sources-based compounds for example, paclitaxel, topotecan, or vincristine, *etc* [16]. So, natural sources have a greater potential for anti-cancer drug discovery. In this study, we chose alkaloids to explore their potential as MDM2-p53 interaction inhibitors. A total of 502 alkaloids of various categories such as indole, isoquinoline, *etc,* were enlisted through literature studies and database searching. The enlisted alkaloids are given as a piece of supporting information [S1 File Enlisted Alkaloids.xlsx].

### 2.2 Bioassay data collection

557 compounds with experimentally measured IC50 values against MDM2-p53 interaction were collected from the PubChem database. The Accession IDs of the bioassay are 587948,1963570, 1919421, 1855504, 1805159, 1804183, 739072, 706602, 621843, 587948,438428, 241501, 594312, 706601, 739073, 1387289, and 1686430 [S2 File Bioassay Data.zip].

A Partial descriptions of the some collected bioassay data are given below.

(a) *PubChem AID: 587948*

Isoindolinone derivatives were examined as an MDM2-p53 inhibitor. 91 compounds with IC50 data for MDM2-p53 interaction inhibition were mentioned in this bioassay.

(b) *PubChem AID: 1963570*

A new spiro compound called 3H-indole-3,2’-pyrrolidin]-2(1h)-one & its derivatives were examined as MDM2-p53 inhibitors.56 compounds with IC50 data for MDM2-p53 interaction inhibition were mentioned in this bioassay.

(c) *PubChem AID:1919421*

14 compounds with IC50 data for MDM2-p53 interaction inhibition were described in this bioassay. Here, substituted of pyrrolo [3,4-d] imidazoles were tested against MDM2-p53 interaction.

(d) *PubChem AID:1804183*

The Spiro[3H-indole-3,2′-pyrrolidin]-2(1H)-one compounds and its derivatives were tested. 107 compounds with IC50 data for MDM2-p53 interaction inhibition were given in this bioassay.

### 2.3 Preparation of bioassay data

The IC50 (µmol) values of the compounds were converted into the negative logarithmic scale (*i.e*. pIC50 = –log (IC50 × 10^–6^) molar (M) for numerical stability. So, a higher predicted pIC50 value of the compounds would indicate a good inhibition potential because of the minus symbol of the pIC50 equation.

In this study, the pIC50 value of ‘morpholinone 27’ was taken as a standard cutoff value that was 7 M. It is a potent MDM2 inhibitor which was evaluated by biochemical and cellular assay [17]. Here, a compound with a predicted pIC50 value greater than or equal to the pIC50 value of ‘morpholinone 27’ was considered a potential inhibitor of MDM2-p53 interaction & lower than the pIC50 value of ‘morpholinone 27’ was considered less effective or ineffective.

### 2.4 Molecular descriptors calculation

There are several types of molecular descriptors, such as 1D, 2D, & 3D. Among them log P, molecular weight, number of hydrogen bond donors, number of hydrogen bond acceptors, number of rotatable bonds, *etc,* are encompassed in 1D molecular descriptors. 2D molecular descriptors denote the presence or absence of specific functional groups in molecules [18].

The Pharmaceutical Data Exploration Laboratory (PaDEL) software can calculate 1D,2D, and 3D (*e.g.,* charged partial surface area, moment of inertia, *etc.*) molecular descriptors along with molecular fingerprints (*e.g.,* daylight, MACCS, *etc.*) [19]. Currently, PaDEL software calculates 1875 descriptors, which include 1444 of 1D and 2D descriptors; 431 of 3D descriptors, and 12 types of fingerprints (16092 bits) [20]. In this study, PaDEL software was implemented to calculate 1875 molecular descriptors of 557 bioassay compounds as well as the alkaloidal library. The calculated descriptors for all the compounds are given as supporting information [S3 File Descriptors of ML Model Building & Testing Dataset.csv, S4 File Molecular Descriptors of the Alkaloids.xlsx].

### 2.5 Data preprocessing

First of all, the low variance features were excluded from the datasets. The missing value of the features-related data can be handled by replacing them by imputing the median (or mean) of each feature [21]. Here, we used the ‘SimpleImputer class from scikit-learn’ for the missing value handling by implementing the ‘mean’ value. Then, the data was standardized by StandardScaler class of ‘scikit-learn’. Subsequently, Anova F method was applied to find out the best features.

### 2.6 Feature selection

The dimension of data is reduced by feature selection and this can reduce noise interference [22]. Thereby, the model performance is enhanced. Here, ‘Anova F’ filter method was applied to determine the most relevant features for the machine learning (ML) models. A p-value of.05 or less is considered statistically important [23]. Here, based on, a p-value of.05 features were selected by the ‘Anova F’ filter method. Above the cutoff, 549 features were selected from 1875 features. The selected features are given as a piece of supporting information. [S5 File Selected Features.csv].

The whole dataset was divided into two sub-datasets. One set contained information regarding 446 compounds that was chosen for training the models. Another set contained information on 111 compounds that was selected as the validation set.

**2.7.** **QSAR models**

#### 2.7.1 *Building of QSAR models.*

The mathematical relationship between molecular features of compounds & their biological activity could be calculated by an ML (machine learning)-based QSAR model [24]. Herein, three machine learning algorithms such as support vector machine (SVM), Random Forest (RF), and Extreme Gradient Boosting (XGBoost) were implemented to construct QSAR models. The Python packages “scikit-learn” & “XGboost” were used to build the SVM, RF, and XGboost ML-based QSAR models. The Python script used for building the best machine learning model is given as a piece of supporting information [S6 File Python Script for the QSAR Model Building.txt].

Support Vector Machine (SVM): Support Vector Machine (SVM) is a statistical method that maps data into high-dimensional space to find a lower-dimensional hyperplane that maximizes the data segregation utilizing a nonlinear kernel [25].

Random Forest (RF) Model: Random forest is a classification technique. It is based upon multiple decision trees and voting rules [26].

XGBoost (XGB): XGBoost could be defined as a decision-tree-based ensemble ML algorithm that uses a gradient-boosting framework [27]. Gradient boosting is one of the most well-known ensemble learning methods and it develops the strong learner gradually by fitting the new weak learner to residuals [28].

#### 2.7.2 *5-fold cross-validation (5FCV).*

Fivefold cross-validation (5FCV) was applied to optimize the ML (machine learning) models. In *5FCV*, the whole training dataset was divided into five subsets of equal size; then one of them (20% of data) was taken as a test set, & the rest of the four sets combined the training set (80% of data ). The procedure was iterated five times, permitting each subset as the test set.

#### 2.7.3 *Hyperparameter tuning.*

Bayesian optimization prevails over traditional methods like grid and random search for hyperparameter tuning. It effectively explores the hyperparameter space, & chooses the best parameters to build a probabilistic model [29]. Here, parameters were optimized with Bayesian optimization method. The best parameters for the generated QSAR models are shown in Table 1.

**Table 1:** The best parameter for the generated QSAR models.

QSAR Models	Best Parameters
SVM	(‘C’, 5681101.17706534), (‘epsilon’, 0.02886207535858715),(‘gamma’,0.003943250134430374)
RF	(‘max_depth’, 9), (‘max_features’, 0.75), (‘min_samples_leaf’, 6), (‘min_samples_split’, 5), (‘n_estimators’, 500)
XGB	(‘colsample_bytree’, 0.8), (‘learning_rate’, 0.2), (‘max_depth’, 3), (‘n_estimators’, 255), (‘subsample’, 0.8907908003700069)

#### 2.7.4 *QSAR models assessment.*

The performance of the generated ML models was evaluated through various statistical parameters such as (a) mean average error (MAE); (b) mean squared error (MSE); (c) and coefficient of determination (R^2^).

Mean absolute error (MAE): The average absolute distance between the observed and the predicted value is called the mean absolute error (MAE). It is expressed by the equation 1.


$$\text{MAE}=\frac{1}{Nres}=\sum_{i=1}^{Nres}\text{min}(|pi-xi|,|360\circ -(pi-xi)|)$$ (1)

where *Nres* is the total number of residues, x represents the observed value, and p represents the predicted value [30].

Mean squared error (MSE): The Mean squared error (MSE) defined as the average of the squares of the difference between the true and predicted values. It is denoted by the equation 2.


$$\text{MSE}=\frac{1}{\text{N}}\sum_{I}^{N}\left(\text{Y}measured,i-\text{Y}predicted,i\right)²$$ (2)

where N indicates the number of features evaluated, Y*measured*, *i* denotes the actual measured value, and Y*predicted, i* is the predicted value for feature *I* [31].

Coefficient of determination (R^2^): The goodness of the fit of an ML model can be measured by the Coefficient of Determination (R^2^). It is expressed by equation 3.


$$R^{2}=1-\frac{\sum_{I}^{N}(Ymeasured,i-Ypredicted,i{)}^{2}}{\sum_{I}^{N}{\left(Ymeasured,i-Y\overset{―}{predicted},i\right)}^{2}}$$ (3)

Where *N* is the number of features, *Ymeasured,i* indicates the actual measured value for feature *i*, *Ypredicted,i* is the predicted value for feature *i*, and *Ymeasured* denotes the average of all measured values [31].

### 2.8 *Structural similarity analysis*

Nine different types of fingerprints such as “standard”, “extended”, “graph”, “hybridization”, “MACCS”, “E-state”, “pubchem”, “shortestpath”, “substructure” were calculated by using “R” programming based cheminformatics toolkits “rcdk”, “fingerprint”. Moreover, the structural similarity of the compounds to the standard drug “SAR405838” was calculated by implementing “rcdk”. The similarity of compounds was calculated by several steps such as (a) similarity calculation of the compounds was performed by using the fingerprints individually one by one; (b) Finally the mean value of all the similarity scores was calculated & the compounds were ranked according to their mean similarity score to the standard drug.

### 2.9 Molecular docking

The crystal structure of MDM2 (PDB ID: 5TRF) in the complex with SAR405838 (*i.e.,* SAR405838 is an experimentally validated MDM2-p53 interaction inhibitor that enters into phase-I clinical -trail) was used for docking study [32]. Molecular Docking was conducted by the MGL software of ‘Autodock-Vina’ to figure out all the possible orientations and conformations of the ligands with the active site residues of the target protein [33]. The distance of X, Y, and Z of the grid box were 11.6437, 21.7593,34.8736 from the center which captured the active site residues of MDM2. The binding affinity of the compounds to MDM2 was calculated in kcal/mol. As a reference, the binding affinity of the SAR405838 was calculated at -10.8 kcal/mol. We validated our docking with ‘Discovery Biovia Studio’ software.

**Fig 1:**
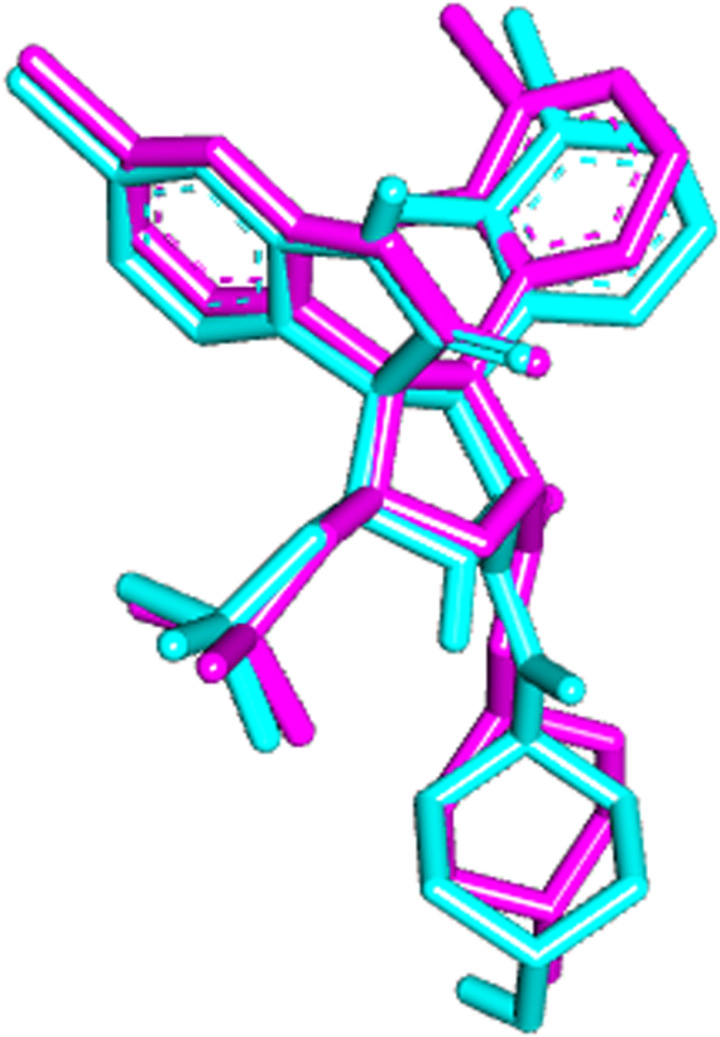
Superimposed of Autodock vina predicted best pose (cyan) of SAR405838 & its co-crystalized form (deep pink).

The spatial arrangement of co-crystallized & the theoretically predicted pose of SAR405838 in the active site are approximately similar (Fig 1). The interacted amino acids of the MDM2 with the ligands were visualized using Chimera software.

### 2.10 Molecular dynamic simulation (MDS)

Molecular Dynamic Simulation (MDS) of two ligand-protein complexes such as SAR405838-MDM2, Geissolosimine-MDM2 was performed by YASARA, version 23.9.29.W.64 throughout 100 nanoseconds(ns) with 401 snapshots and the AMBER14 force field [34]. The transferable intermolecular potential3 points (TIP3P) water model was used to solvate the protein-ligand complexes [35]. The physiologic condition in MDS was maintained with the addition of Na + and Cl− ions. All the data obtained from MDS is given as a piece of supporting information. [S7 File MDS.zip].

### 2.11 Pharmacokinetics (PK) & toxicity (T) prediction

Many drugs fail in clinical trials because of their unexpected toxicity. So, in an early phase of drug discovery, the compounds selection with desired pharmacokinetics is crucial [36]. Online-based tools such as SwissADME (*i.e.,* a charge-free web tool that is used for predicting drug-likeness, pharmacokinetics, and medicinal chemistry friendliness), admetSAR, ADMETlab, etc are used for the prediction of ADMET of compounds [37]. Here, we used SwissADME & ADMETlab for the prediction of the pharmacokinetic properties of the compounds.

## 3. Results and discussion

### 3.1 Machine learning-based QSAR models construction & implementation

#### 3.1.1 *Diversity analysis of the dataset.*

The robustness of the ML model’s prediction ability depends on the chemical diversity of compounds that are used for building ML dataset like training and testing datasets [38]. The diversity of compounds has an impact on the prediction ability of a ML model [39].

**Fig 2:**
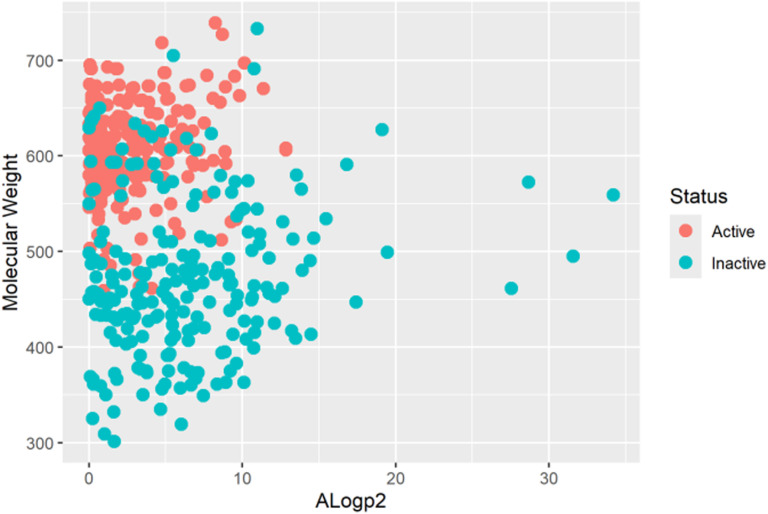
The chemical space of the ML model-building dataset is defined by the molecular weight on the y-axis and AlogP2 on the X-axis.

Usually, chemical diversity is measured by chemical spaces [38]. Therefore, we used molecular weight & AlogP to diversity analysis of the dataset Fig 2.

It is seen from Fig 2 that the active and inactive compounds (*i.e.,* the pIC50 value equal or greater than morpholinone 27 is counted as active vice versa) in the ML model-building dataset are chemically diversified Fig 2. The calculated molecular weight and AlogP2 are given as supporting information [S8 File MW & AlogP2 of Active and Compounds.csv].

#### 3.1.2 *Feature selection.*

**Fig 3:**
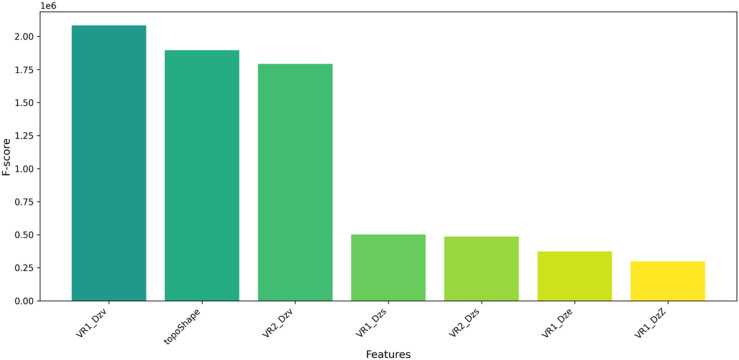
The relative importance of the several selected features along with Anova F Scores.

By implementing ANOVA F scores features selection method, ‘549’ features were selected. Here, some of the top selected feature’s relative importance based on F Scores are mentioned in Fig 3.

Fig 3 depicts the relative importance of the features to the target variable. From Fig 3, it is seen that several highly correlated features to the target variable (pIC50) are VR1_Dzv, topoShape, etc. 

#### 3.1.3 *QSAR models construction.*

For ML model validation purposes, k-fold, and jackknife cross-validation (CV) are widely employed [40]. Moreover, cross-validation can mitigate overfitting issue of ML models [41]. Here, we used k-fold cross-validation (KCV) method (i.e. where k was set as k=5; 5FCV) on the selected features of the dataset for the effectiveness of the ML models where the whole dataset was divided into 5 folds. At each of the 5FCV iterations, the 1-fold was used as a test set, and the remaining 4-folds were used as a training dataset.

#### 3.1.4 *QSAR models performance evaluation.*

To identify the best QSAR model, the several statistical parameters such as the coefficient of determination (R^2^), mean absolute error (MAE), and mean squared error (MSE) for the generated ML models are evaluated that represented in Table 2, Fig 3.

**Table 2:** The results of five-fold cross-validation.

Algorithms	Testing sub-sets	R^2^	MAE	MSE
SVM	Subset 1	0.83649063	0.436418	0.38199725
	Subset 2	0.81301568	0.47835984	0.41377118
	Subset 3	0.78846873	0.52479301	0.48541211
	Subset 4	0.81430528	0.50155052	0.4361919
	Subset 5	0.67952261	0.63047844	0.73115551
RF	Subset 1	0.87567315	0.38777536	0.28743891
	Subset 2	0.83135027	0.43628224	0.38176372
	Subset 3	0.83924314	0.49150825	.37794994
	Subset 4	0.78850292	0.50962928	0.48650685
	Subset 5	0.81269364	0.49851316	.44270255
XGB	Subset 1	0.85527726	0.42698622	0.33810716
	Subset 2	0.8058009	0.46178621	0.42973652
	Subset 3	0.82594667	0.51359725	0.39940948
	Subset 4	0.81610026	0.48505451	0.43197554
	Subset 5	0.77020838	0.53539217	0.52425979

**Fig 4:**
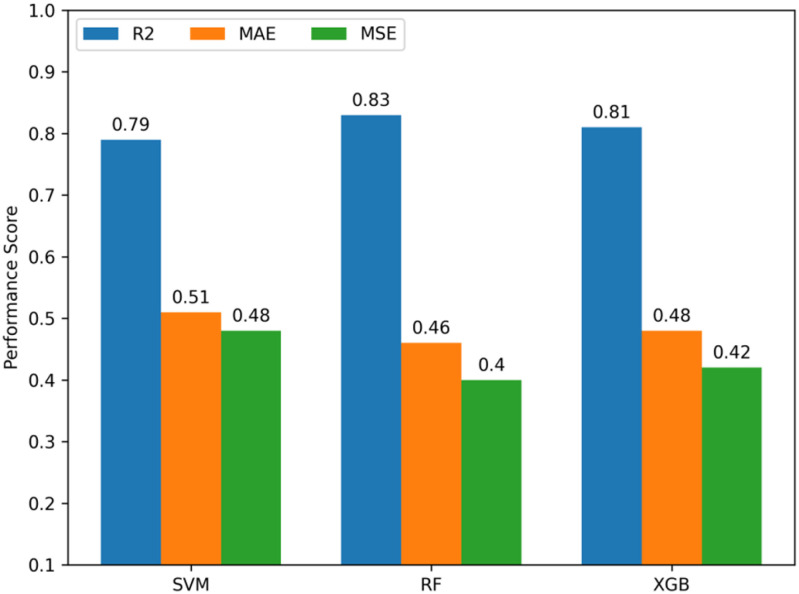
The performance indicator of the generated QSAR models. Here, R^2^ = Coefficient of determination (R^2^), MAE = Mean Absolute Error, RMSE = Root Mean Squared Error. SVM = Support Vector Machine, RF = Random Forest, XGB = Extreme Gradient Boosting.

The ‘RF’ based ML model gets the highest R^2^ values, ranging from 0.78850292 to 0.87567315, following the ‘XGB’, ‘SVM’ range from 0.77020838 to 0.85527726, 0.67952261 to 0.83649063 respectively Table 2. Regarding the MAE, the lowest range is obtained for ‘RF’ model, followed by ‘XGB’, then ‘SVM’ Table 2 and for MSE, the highest value is obtained for ‘SVM’ Table 2. However, these metrics are averaged to get a comprehensive overview of the ML-based QSAR models that are depicted in Fig 4.

The RF algorithm-based QSAR model exhibits better performance with a coefficient of determination (R^2^) value of 0.83 following XGB and SVM (Fig 4). A higher value of R^2^ signifies a good performance, a higher predictive ability, and a greater reliability of an ML regression model [42]. So, it is determined that the RF algorithm-based QSAR has the greater predictive ability and reliability than the others.

A good MAE value of an ML model is always small. The proximity of MAE values to zero indicates a good accuracy. It is seen from Fig 4 that, among the generated QSAR models the MAE value of the RF regression based QSAR model is comparatively lower than XGM, SVM, and that demonstrates the it has thegood accuracy.

The MSE values of the SVM, RF, XGB are 0.48, 0.4, and 0.42 respectively (Fig 4). A higher MSE value denotes a larger error in an ML model performance. On the other hand, a lower MSE value of an ML model points to a higher & reliable prediction ability [42]. So, the lower MSE of the RF algorithm-based QSAR indicates the it has relatively the better predictive ability (Fig 4).

#### 3.1.5 *Validation of the generated models’ performance by the unseen data.*

**Fig 5:**
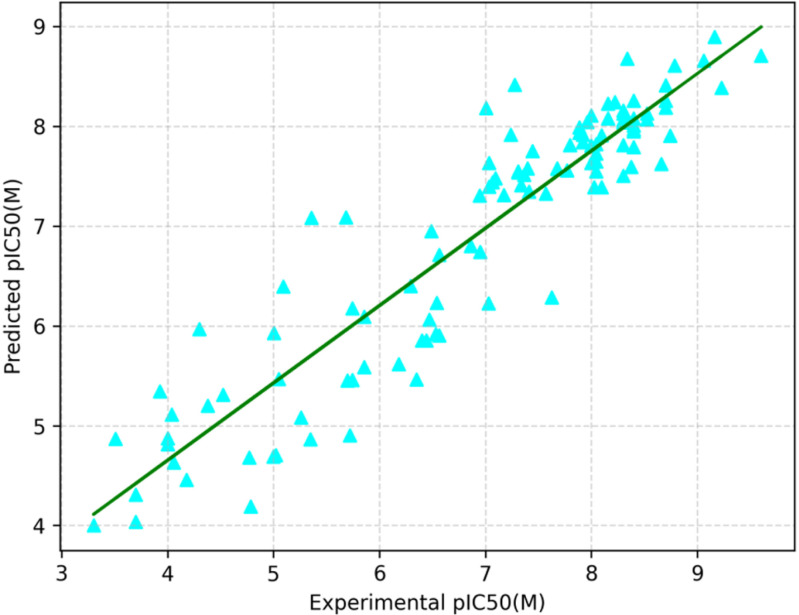
The experimental and RF regression-based QSAR model’s predicted pIC50 values of the compounds in the unseen dataset.

We assessed the performance of the trained RF regression based QSAR model on the unseendataset. The trained-RF QSAR model predicted the target variable of the dataset. By comparing the predicted target variable with the actual target value, the model performance was evaluated. In this regard threeregression metrics such as R², MAE, and MSE were calculated [S9 File The model performance on the unseen dataset.docx]. Their value is closer to the result obtained through the cross-validation. This evaluation provides the predictive capabilities of the RF regression-based QSAR model on the unseen dataset. Here, the predicted and experimentally pIC50 values by the RF regression-based QSAR for the unseen dataset is depicted in the Fig 5.

It is seen from the Fig 5 that there is a closeness between the experimental & predicted pIC50 values, so it is understood that the RF regression-based QSAR has better performance.

Here, the RF regression-based QSAR is more statistically significant than the others. Therefore, it was selected for the virtual screening of the in-house building alkaloids library to predict potential MDM2-p53 interaction inhibitors.

#### 3.1.6 *Implementation of QSAR regression model on the in-build alkaloids library.*

**Fig 6:**
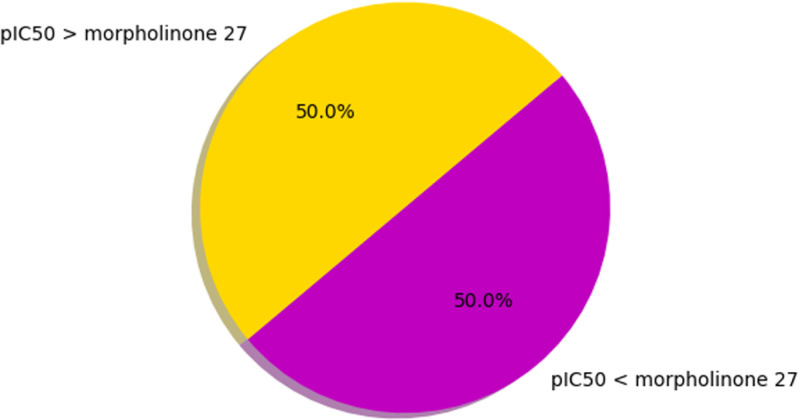
The predicted active & inactive alkaloids percentage against MDM2-p53 interaction inhibition by RF regression-based QSAR model.

The RF regression-based QSAR model was implemented to screen the in-house building alkaloids library (*i.e*., it contained 502 alkaloids comprising various categories such as indole, pyrrole, *etc.*). The screening process was based on the ranking of the predicted pIC50(M) values of the compounds. The pIC50 value of morpholinone 27 (7 M) was taken as a cutoff. The compounds with pIC50 values above or equal to the cutoff were considered potential MDM2-p53 interaction inhibitors, and those below the cutoff were considered inactive. The percentage of the predicted active and inactive alkaloids as MDM2-p53 interactiions inhibitors by RF regression-based QSAR is shown in Fig 6.

Among 502 compounds of the in-house building alkaloid library, 251 (50%) compounds are predicted to be potent MDM2-p53 inhibitors (Fig 6) and 251(50%) compounds have predicted values below the cutoff (Fig 6) is obtained as inactive. The predicted pIC50(M) value of all the alkaloids was given as supporting information [S10 File The Predicted pIC50 Value of the Alkaloids.xlsx].

### 3.2 Cheminformatics analysis

#### 3.2.1 *Structural similarity analysis of  alkaloids to SAR405838.*

The Similar Property Principle (SPP) describes that “Compounds having structural similarity to each other may have similar therapeutic activity” [43]. Various fingerprints are used such as MACCS, *etc* for similarity calculation among compounds. Integrating different fingerprints is important for similarity calculation [44]. Different coefficients are used to calculate the structural similarity analysis. Here the ‘Dice Coefficient’ was used for the structural similarity analysis. It is expressed in equation 4;


$$SA,B=\frac{2c}{a+b}$$ (4)

Here, S represents the ‘Dice Coefficient’, a = number of unique fragments in the compound ‘A’ & b = number of unique fragments in the compound ‘B’. c = numbers of unique fragments shared by the compounds A and B [45].

Nine molecular fingerprints such as “standard”, “extended”, “graph”, “hybridization”, “MACCS”, “E-state”, “pubchem”, “shortestpath”, “substructure”, were individually implemented to calculate the structural similarity to the standard. We calculated the structural similarity analysis of the compounds in two steps. First of all, molecular fingerprints were implemented one by one, to calculate the similarity of the alkaloids to the standard drug.

**Fig 7:**
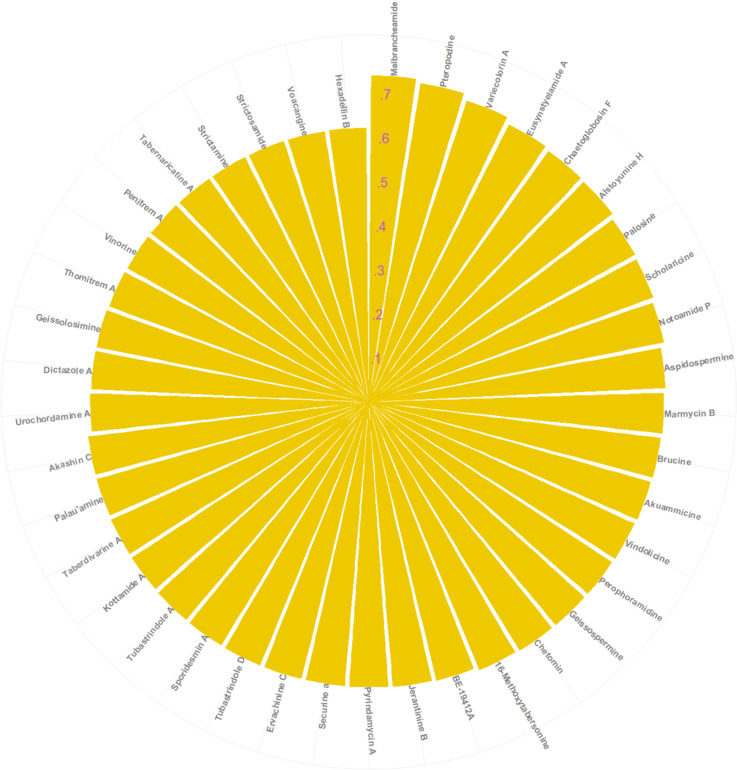
Structural Similarity of the 41 alkaloids to SAR405838.

Secondly, the mean value of the similarity scores was calculated by combining all the calculated similarity scores. Similarity scores among the compounds greatly depend on which fingerprints are used. It is not possible to fix a universal cutoff criterion for similarity calculation [46]. So, we randomly set a cut-off for the mean similarity value of 0.62. The similarity scores range from 0 to 1. The compounds have the similarity scores closer to 1 (*i.e.,* the similarity score of the SAR405838 was taken as 1), denoting the most similarity to SAR405838. On the other hand, the compounds havesimilarity scores near to zero indicate structural dissimilarity to the standard. Above this cut-off, out of 251 compounds, 41 compounds are obtained as the structurally most similar to the SAR405838. The structural similarity of the 41 alkaloids with SAR405838 is shown in Fig 7.

**Fig 8:**
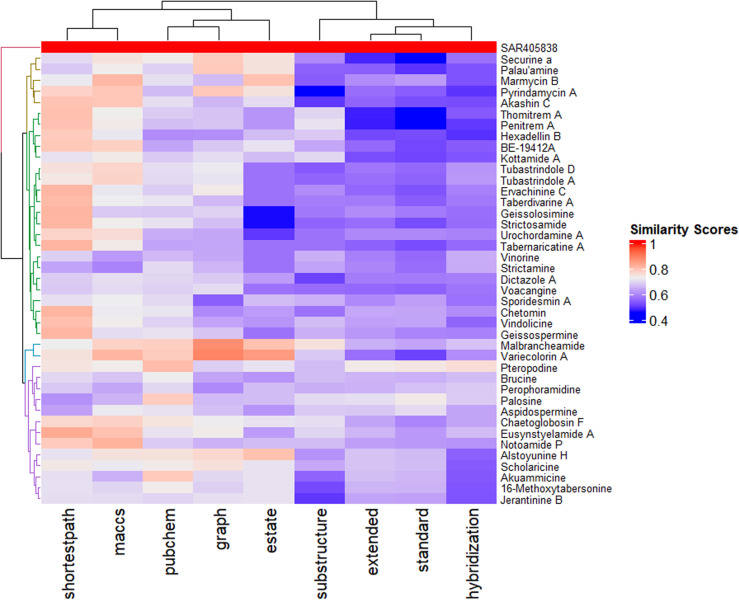
The cluster analysis of structurally similar 46 alkaloids to SAR405838. The Dendrogram & heatmap of the distance matrix are both colored according to the structural similarity (orange/red = similar, blue/violet = dissimilar).

Malbrancheamide exhibits ~ .74% structural similarity (similarity scores = ~.0.74) to the standard (Fig 7). The lowest similarity score is observed for Hexadellin B (Fig 7). All the compound’s similarity values are given as supporting information [S11 File Structural Similarity Scores.xlsx]. A comparative similarity of the 41 alkaloids based on the different fingerprints is shown in Fig 8.

In Fig 8, Rows (y-axis), hierarchical clustering dendrogram shows the correlation of the compounds based on their overall similarity scores. The compounds clustered together are structurally more similar. For example, Stictosamide, Gesissolosimine, form a cluster & this cluster link to the another cluster based on their relative similarity scores. However, the clusters that are closer to the standard are chosen for molecular docking purposes to check whether they have potential binding modes to MDM2 or not.

Here, Column (x-axis) represents the molecular fingerprints by which structural similarity was calculated. Based on the ‘Shortestpath’, ‘MACCS’, pubChem, ‘graph’, and ‘estate (E-state)’, 41 compounds show more structural similarity to the standard than the other fingerprints (Fig 8).

However, the structurally identical compounds will likely adopt similar binding modes to the target protein [46].

### 3.3 Molecular docking analysis

The disruption of MDM2-p53 interaction, by small molecules is an attractive therapeutic approach for treating various cancers [47]. Specifically, the hydrophobic surface cleft (*i.e.,* comprising amino acid residues Leu54, Leu57, Gly58, Ile61, Met62, Try67, Gln72, His73, Val75, Phe91, Val93, His96, Ile99, Tyr100) of the N-terminal domains of MDM2 interacts with p53 wild types [48].

The most structurally similar compounds to SAR405838 were undergoing molecular docking-based screening. This screening was conducted targeting the amino acid residues of the N-terminal domains of MDM2. The binding affinity score of SAR405838 was calculated as -10.8 kcal/mol and it was set as the cutoff. Here, the compound obtained with a higher binding score is shown in Table 3.

**Table 3:** Binding affinity Score.

Compounds	Category	Binding Affinity (Kcal/mol)
SAR405838	Standard	-10.8
Geissolosimine	indole alkaloid	-10.9

Among the most similar compounds that were obtained from the similarity analysis, only ‘Gessiolosimine’ (an indole alkaloids) shows a higher binding affinity Table 3. The docking files of the all compounds are given as a piece of supporting information. [S12 File Molecular Docking Files.zip].

**Fig 9:**
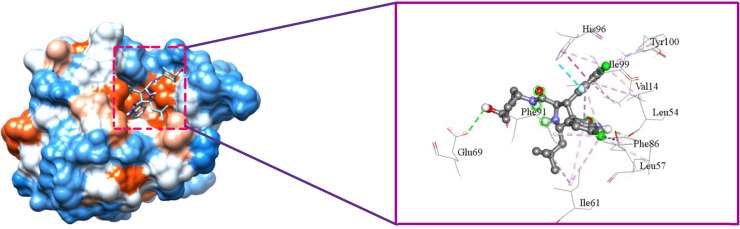
The interaction between SAR405838 & MDM2.

**Fig 10:**
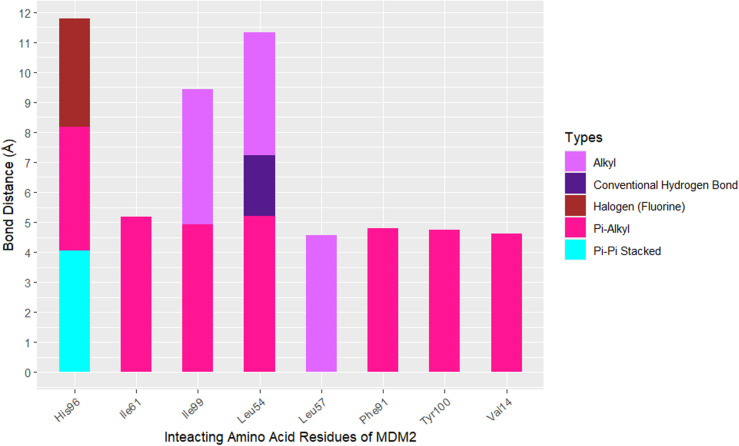
The bond distance between the SAR405838 and amino acid residues of MDM2.

**Fig 11:**
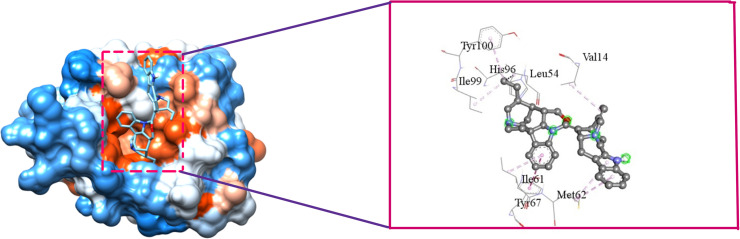
The interaction between MDM2 & Geissolosimine.

**Fig 12:**
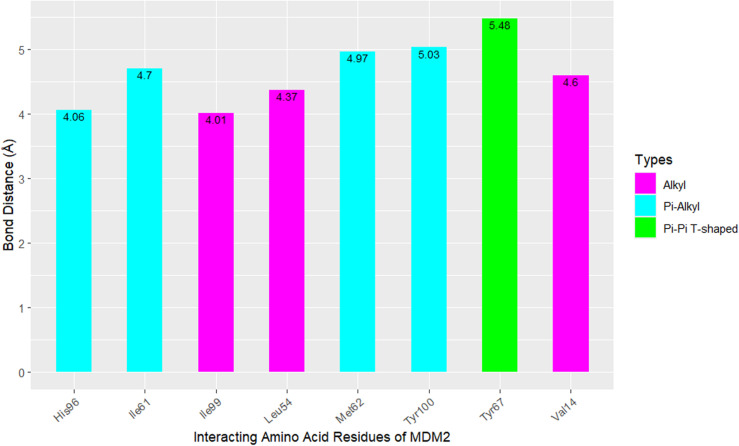
The bond distance between the Geissolosimine and amino acid residues of MDM2.

Fig 9, Fig 10, Fig 11, and Fig 12 depict the interaction of SAR405538, and Gessiolosimine with the amino acid residues of MDM2.

SAR405838 interacts with the seven N-Terminal domain residues of MDM2 such as Leu54, Leu57, Ile61, Phe91, His96, Ile99, and Tyr 100 (Fig 9). Moreover, it binds with the other sites residues of MDM2 like Val14, Glu69, and Phe86 (Fig 9). The bond distance between SAR405838 & interacted amino acid residues are shown in Fig 10.

SAR405838 forms the pi-alkyl interaction with the Val14, Leu54, Ile61, Phy91, His96, Ile99, and Tyr100 amino acid residues of MDM2 in the distance of 4.62 Å, 5.21Å, 5.19 Å, 4.79 Å, 4.13 Å, 4.93 4.74 Å respectively (Fig 10). It also forms the alkyl interaction with Leu54, Ieu57, and Ile99 amino acid residues of MDM2 in the distance of 4.1Å, 4.56Å, and 4.5Å respectively (Fig 10). Moreover, SAR405838 forms the hydrogen bond interaction with the Leu54, the Pi-Pi Staked & halogen bond interaction with the His96 amino acid residues of MDM2 (Fig 10).

Geissolosimine interacts with several N-terminal domain residues of MDM2 such as Leu54, Ile61, Met62, Try67, His96, Ile99, and Tyr100 (Fig 11). Moreover, it also interacts with Val14 (Fig 11).

It forms the Pi-Alkyl interaction with the Ile61, Met62, His96, & Tyr100 amino acid residues at a distances of 4.7Å, 4.97Å, 4.06Å, 5.03Å respectively (Fig 12). Geissolosimine also forms the alkyl interaction with Val14, Leu54, and Ile99 amino acid residues of MDM2 at distances of 4.6Å, 4.37Å, 4.01Å respectively. (Fig 12). Moreover, it forms the pi-pi T-shaped interaction with Tyr67 of MDM2 at the distance of 5.48Å (Fig 12).

SAR405838 & Geissolosimine interact with the same quantities of the N-terminal domain of amino acid residues of MDM2 (Fig 9, Fig 11). However, SAR405838 skips to interact with Met62, and Try67 of MDM2 whereas Geissolosimine interacts with those amino acid residues (Fig 9, Fig 10). Geissolosime does not binds with Leu57, Phe91 of MDM2 but SAR405838 binds with those residues. Herin the N-Terminal amino acid residues of the MDM2 which are common for SAR405538 & Geissolosimine interaction are shown in Table 4.

**Table 4:** The common interacting active site amino acid residues of MDM2 for SAR405838 and Geissolosimine.

Compounds	Amino Acid Residue	Bond Type	Distance(Å)
SAR405838	Leu54	Alkyl	4.10
	Ile61	Pi-Alkyl	5.19
	His96	Pi-Alkyl	4.13
	Ile99	Pi-Alkyl	4.93
	Tyr100	Pi-Alkyl	4.74
Geissolosimine	Leu54	Alkyl	4.37
	Ile61	Pi-Alkyl	4.70
	His96	Pi-Alkyl	4.06
	Ile99	Pi-Alkyl	4.01
	Tyr100	Pi-Alkyl	5.03

Although Geissolosimine interacts with Leu54, Tyr100 at a bit of a higher distance than SAR405838, the bond distances between Geissolosimine & other N-terminal amino acid residues of MDM2 such as Ile61, His96, Ile99 is lower than SAR405838 (Table 4). The smaller bond distances between the ligands & proteins is considered a stronger interaction. So, we anticipate that Geissolosimine may have the potential to interact more strongly with MDM2 than SAR405838. However, the pragmatic interactions between small molecules & receptors depend on the experimental technology [49]. For examples X-ray Crystallography, *etc*. In the next step, we performed MDS of 100 ns to evaluate the binding stability of SAR405838 & Geissolosimine to MDM2.

### 3.4 Molecular dynamics simulation (MDS)

The dynamic characteristics of a protein-ligand complex is studied through the Molecular Dynamics Simulation (MDS). Here, the dynamic behavior of the two protein-ligand complexes (*i.e.,* MDM2-SAR405838 and MDM2-Geissolosimine), are investigated by exploring several structural and systemic parameters, such as root mean square deviation (RMSD), radius of gyration (Rg),root mean square fluctuation (RMSF), and hydrogen bond (H-bond) analysis.

#### 3.4.1 *Root mean square deviation (RMSD) analysis.*

Upon a ligand binding with a protein, a structural dynamic occurs within the protein structure. In this respect, Root Mean Square Deviation (RMSD) is dedicated to evaluate structural dynamic alteration [50]. RMSD is expressed by the equation 5.


$$RMSD=\sqrt{\frac{\sum_{i=1}^{n}Ri*Ri}{n}}$$ (5)

where Ri is a vector linking the positions of atom i [of N atoms] in a reference snapshot [51].

**Fig 13:**
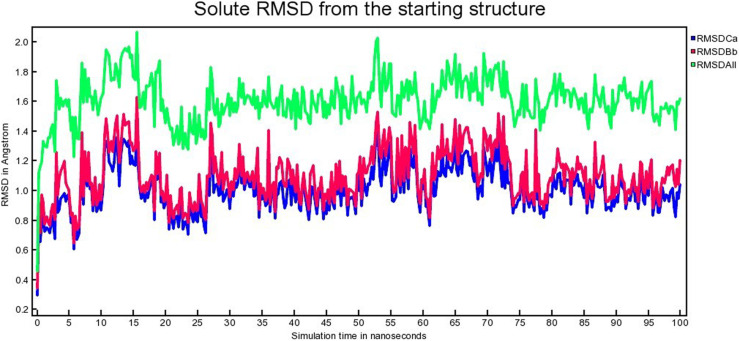
The time series of the RMSD of of Calpha [RMSDCa], backbone [RMSDBb] and all-heavy atom [RMSDAll] of MDM2- SAR405838 complex.

**Fig 14:**
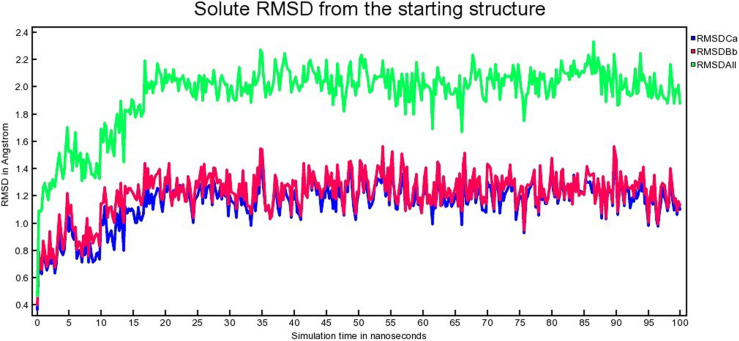
The time series of the RMSD of of Calpha [RMSDCa], backbone [RMSDBb] and all-heavy atom [RMSDAll] of MDM2- Geissolosimine complex.

The atomic RMSDs of Calpha [RMSDCa], backbone [RMSDBb], and all-heavy atom [RMSDAll] for both proteins and the ligands (*i.e.,* MDM2-SAR405838 and MDM2-Geissolosimine) for 100 ns were calculated. Figs 13, 14 represent the MDM2-SAR405838 and MDM2-Geissolosimine RMSD respectively.

It is possible to estimate how long a ligand inhibit a proteins by calculating the binding stability  [52]. A lower RMSD deviation implies, a more stable protein-ligand complex [53]. On the other hand, a greater highest RMSD deviation indicates, a ligand may escape from a protein. It is seen from Fig 13 that the MDM2- SAR405838 complex is stable at the ranges of ~ 20–25 ns, again ~35–50 ns, and ~ 90–100 ns respectively. MDM2- SAR405838 complex shows fluctuation at the ranges of ~ 1–19 ns, ~ 26–34 ns, and ~ 51–89 ns (Fig 13) which implies the SAR405838 is detached from MDM2 in these ranges.

It is seen from Fig 14, after ~15 ns, MDM2- Geissolosimine complex shows a plateau till 100 ns that means this protein-ligand complex is more stable at this range. By comparing Fig 13, and 11B, it is understood that, the stable binding range of Geissolosiminne to MDM2 is higher than SAR405838.

Since Geissolosimine has the better predictive binding stability to MDM2 than SAR405838, it may have better inhibition potential of MDM2- p53 interaction. However, experimental procedures have to be performed to validate theclaim.

#### 3.4.2 *Radius of gyration (Rg).*

**Fig 15:**
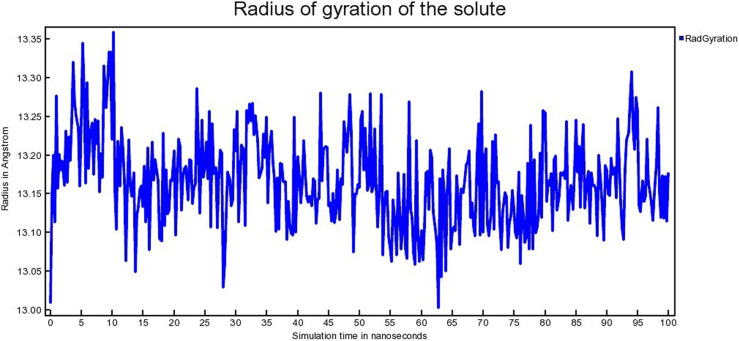
The Radius of gyration (Rg) of MDM2- SAR405838 complex.

**Fig 16:**
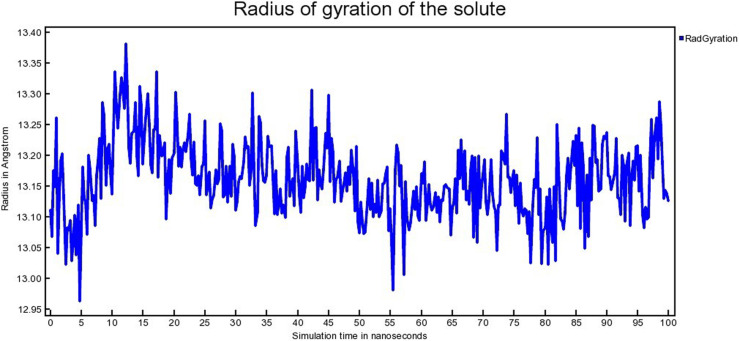
The Radius of gyration (Rg) of MDM2- Geissolosimine complex.

The radius of gyration (Rg) relates to the conformational state of a protein. It indicates the compactness as well as the folding of a protein [54]. The radius of gyration (Rg) for MDM2- SAR405838, MDM2- Geissolosimine complexes are shown in Fig 15 & Fig 16, respectively.

The MDM2- SAR405838 complex shows a lower radius of gyration (Rg) from ~ 10 ns to 25 ns, ~ 55ns to 60 ns respectively & a greater Rg from ~1 ns to 10 ns, ~ 25ns to 55 ns, then after ~ 61 ns to ~ 100 ns (Fig 15).

MDM2- Geissolosimine complex shows a lower Rg from ~1ns to 10 ns, then its Rg suddenly increases from ~10 ns to 15 ns. Then its Rg decreases from ~20 ns to 100 ns (Fig 16). A lower Rg value for a protein-ligand complex indicates the lower conformational changes of a protein upon a ligand binding in molecular dynamics simulation in terms of more compactness [55]. Since the MDM2- Geissolosimine complex shows relatively lower Rg than MDM2- SAR405838 complex, we persume that Geissolosimine may be strongly bound with MDM2.

#### 3.4.3 *Root means square fluctuation (RMSF).*

**Fig 17:**
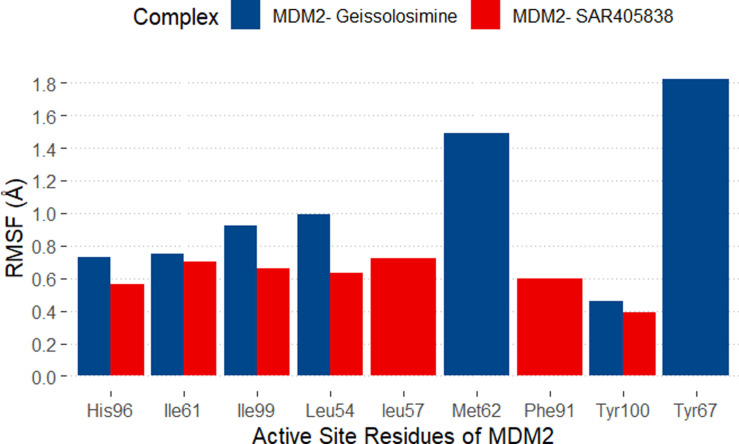
The RMSF of the active site residues of the MDM2.

Herein, the RMSF of the active site residues of MDM2 after the ligands (*i.e.,* Geissolosimine, SAR405838) binding over the simulation time is plotted in Fig 17.

The RMSF range of active site residues of MDM2 after SAR405838 binding is ~ .7 Å to ~ .5 Å. Conversely, the RMSF of the active site residues of MDM2 after Geissolosimine binding is ~ 1.8Å to.45Å. Compared to SAR405838, the Leu54, Ile61, His96, and Tyr100 residues of MDM2 show approximately minimal level RMSF upon Geissolosimine binding (Fig 17), although Met62, Try67, and Ile99 show comparatively higher RMSF (Fig 17). The higher RMSF reflects the displacement of a ligand [56]. Since, we performed rigid docking between MDM2 and ligands, the higher RMSF value of the Met62, Try67, and Ile99 of MDM2 after Geissolosimine binding indicates that the ligand may displace from those residues over the simulation time. Besides these amino acid residues, the interactions of Geissolosimine with Leu54, Ile61, His96, and Tyr100 residues of MDM2 are predicted as significant for inhibiting MDM2-p53 interaction.

#### 3.4.4 Hydrogen bond analysis.

**Fig 18:**
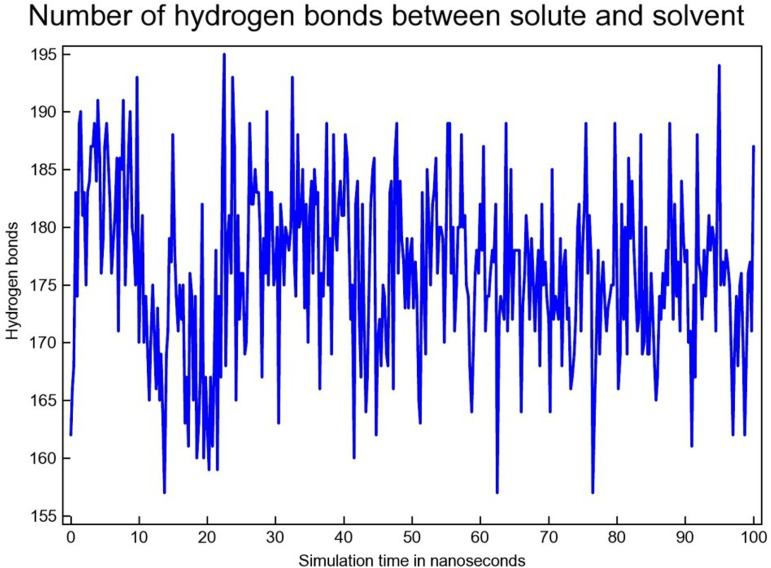
The H-bond between MDM2 & SAR405838.

Through 100 ns MDS, the number of H-bonds between SAR405838 lies in the range of ~ 180–195 (Fig 18).

**Fig 19:**
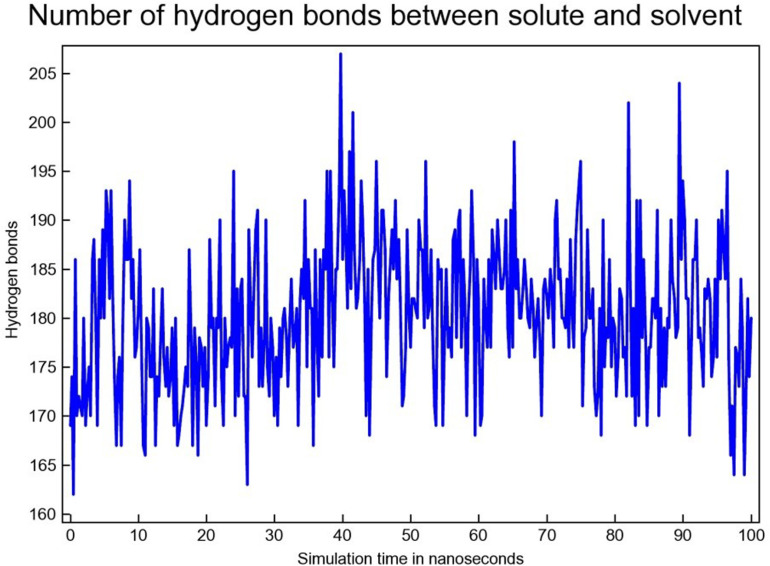
H-bond between the MDM2 & Geissolosimine.

From 10 to 40 ns, the number of H-bond formations between MDM2 & Geissolosimine lies between the range of ~ 170 to ~ 180 (Fig 19). Then between 45–80 ns, the H-bond quantities of MDM2 & Geissolosimine is 180–190 (Fig 19). From 10 to 40 ns and 45–80 ns, the H-bond formation quantities between MDM2-Gessolosimine shows a comparatively higher level (Fig 19).

It is understood from Fig 18, and Fig 19 that the maximum time of the simulation, the quantity of the H-bond formation between MDM2-Geissolosimine is lower than the of MDM2-SAR405838. The decrease of H-bonds formation between a protein & a ligand denotes the ligand occupy intramolecular space in a proteins [50]. Thus, Geissolosimine may occupy a greater intramolecular space in the MDM2 than SAR405838.

Overall, the MDS analysis suggests that Gessiolosimine may have a comprehensive binding stability with MDM2.

### 3.5 *Pharmacokinetic & drug likeness analysis*

#### 3.5.1 *Pharmacokinetic analysis.*

Several studies have identified natural source-based inhibitors of MDM2-p53 interaction or down-regulators of MDM2. For example, Hoiamide D is a natural MDM2-p53 interaction inhibitor, and Genistein is a down-regulator of MDM2[57,58]. Here, the pharmacokinetic parameters of the Geissolosimine & other MDM2-p53 interaction inhibitor or modulator were calculated using SwissADME. Table 5 summarizes the pharmacokinetics analysis of Geissolosime, Hoiamide D, and Genistein.

**Table 5:** The pharmacokinetic Parameters comparison of Geissolosimine, Hoiamide D, Genistein.

Parameters	MDM2-p53 interaction inhibitor or MDM2 Down-regulator		
Geissolosimine	Hoiamide D	Genistein
BBB permeant	Yes	No	No
GI absorption	High	Low	High
CYP1A2 inhibitor	No	No	Yes
CYP2C19 inhibitor	No	No	No
CYP2C9 inhibitor	No	No	No

Although Geissolosimine is predicted as a BBB penetrator, Hoiamide D and Genistein are not predicted to do so (Table 5). The bioavailability of a drug is directly proportional to the GI absorption. It is seen from Table 5 that Geissolosimine has a higher GI absorption probability. So, it may have a better bio-availability than Hoiamide D, Genistein. Moreover, Geissolosimine is not predicted to be a CYP1A2 inhibitor as well as Hoiamide D (Table 5). But Genistein is predicted as a CYP1A2 inhibitor (Table 5). Cytochrome P450 enzymes like CYP1A2, CYP2C19, CYP2C9 are the prominent phase I liver enzymes [59]. Therefore, the inhibition of Cytochrome affects its activity which directed to toxicity [59]. From the above pharmacokinetic parameters analysis, it is concluded that Geissolosimine may have a better pharmacokinetic possibility than Hoiamide D, and Genistein.

#### 3.5.2 *Drug likeness analysis.*

The ‘Lipskin role of five’ describes that a successful orally active drugs has the following parameters: molecular weight (MW) ≤ 500, H-bond donors ≤5, H-bond acceptors ≤5, logP ≤5 [60]. The ADMETlab predicted the molecular weight of Geissolosimine is 572.35 Dalton, H-bond acceptors are 5, H-bond donors are 1, & predicted LogP is 3.905. Though Geissolosimine has a greater MW, the other criteria of the Lipskin role of five’ are followed which indicates this compound may have potential drug-like properties.

## 4. Conclusion

In this study, we conducted an integrated virtual screening on the in-house building alkaloids library. The RF algorithms-based QSAR model revealed that out of 502 alkaloids, 251 had better predictive pIC50 values against MDM2-p53 interaction inhibition. Subsequently, structural similarity analysis showed only 41 compounds from 251 had good similarity scores above the cutoff. Then, molecular docking study of the 41 alkaloids was performed and Geissolosimine showed a better binding affinity. Further molecular dynamic simulation (MDS) analysis indicated that Geissolosimine had a comprehensive binding stability to the MDM2 than SAR405838. Moreover, the pharmacokinetic and drug-likeness analysis signified that Geissolosimine had the potential to be a drug-likeness compound. However, solely computational analysis is not sufficient for inhibitory activity prediction. Thus, the computational study mentioned here is suggested as worthy of extensive experimental testing such as in-vitro biological activity testing against MDM2 as well as in vivo testing.

## 5. Current status of the geissolosimine

**Fig 20:**
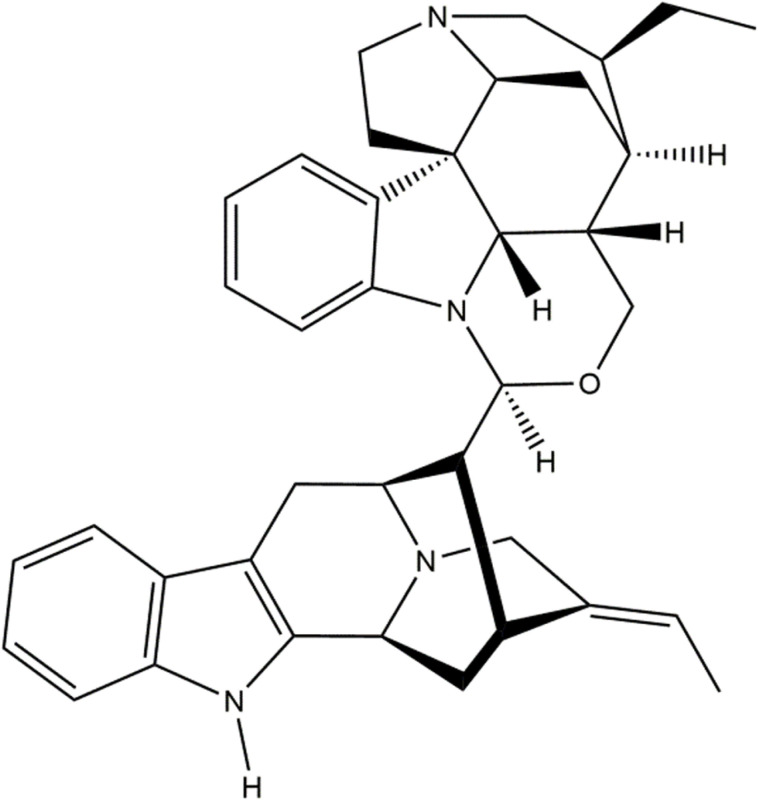
The Structure of Geissolosimine.

Geissolosimine is an indole alkaloid which was identified in *Geissospermum vellosii* & its *in-vitro* antiplasmodial activity against *Plasmodium falciparum* was examined [61]. Moreover, Geissolosimine could be feasible for *de novo* synthesis [62]. Here, the structure of Geissolosimine is depicted in below [62] (Fig 20).

## Supporting information

S1 File Enlisted Alkaloids. (XLSX)

S2 File Bioassay Data. (XLSX)

S3 File Descriptors of ML Model Building & Testing Dataset. (CSV)

S4 File Molecular Descriptors of the Alkaloids. (XLSX)

S5 File Selected Features. (XLSX)

S6 File Python Script for the QSAR Model Building. (TXT)

S7 File MDS. (DOCX)

S8 File MW & AlogP2 of Active and Compounds. (ZIP)

S9 File The model performance on the unseen dataset. (XLSX)

S10 File The predicted pIC50 value of the alkaloids. (XLSX)

S11 File Structural Similarity Scores. (XLSX)

S12 File Molecular Docking Files. (ZIP)
