# Supplementary material for: Anti-cancer activity elucidation of geissolosimine as an MDM2-p53 interaction inhibitor: An in-silico study
Source: PLoS One. 2025 May 8;20(5):e0323003. doi: 10.1371/journal.pone.0323003 (PMC12061181; doi:10.1371/journal.pone.0323003)

**Our procedure for Molecular Docking Validation:**

# Open discovery studio;

# Open the experimentally protein-ligand complex (PDB ID: 5trf)


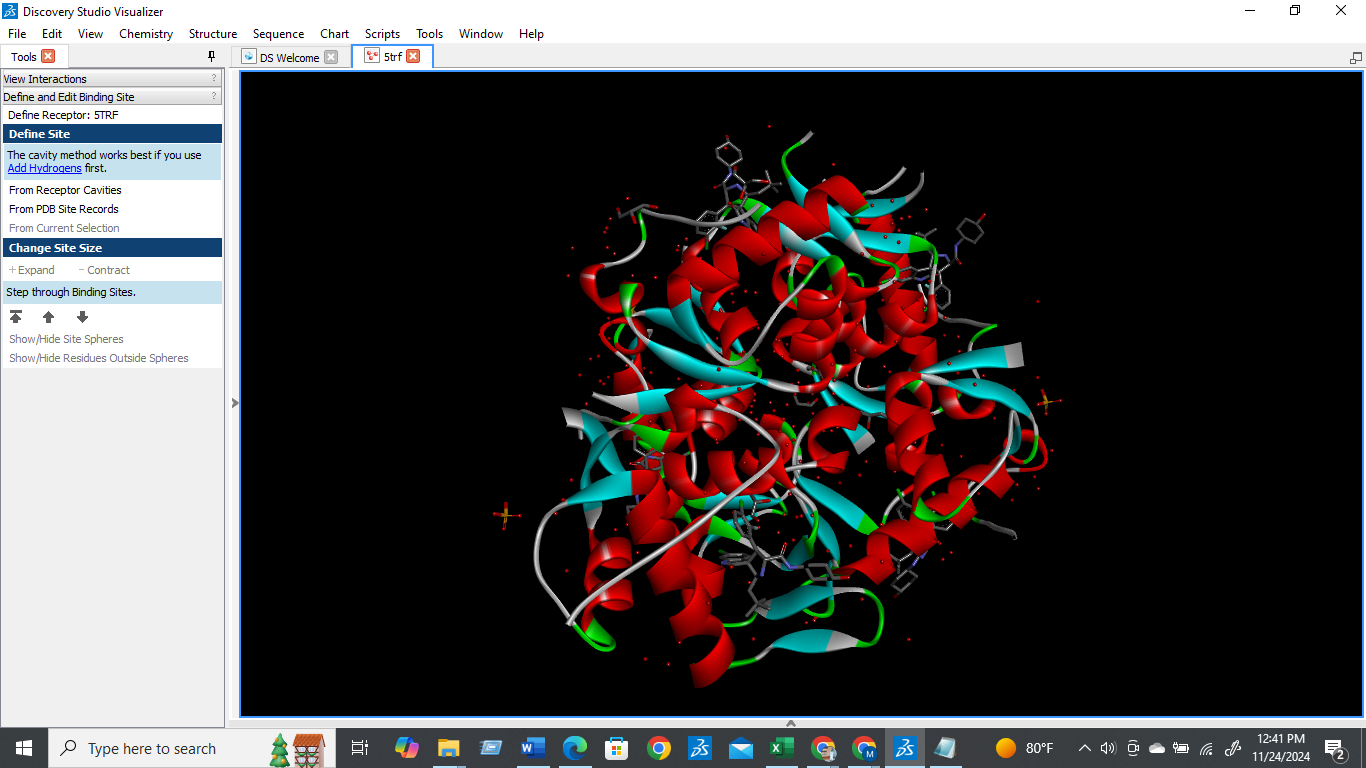


# Chose the C chain where the ligand was bound to the active site,


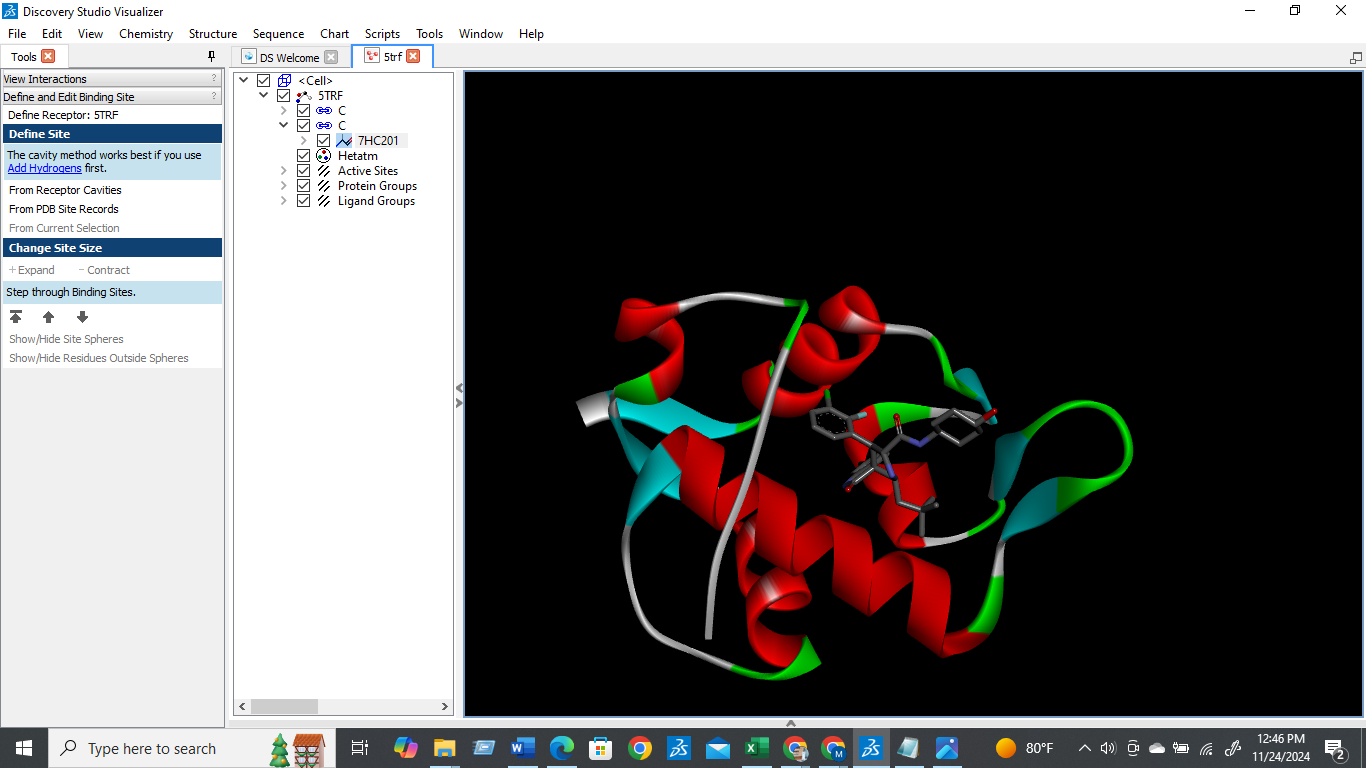


# Click on the ligand which are yellow marked;


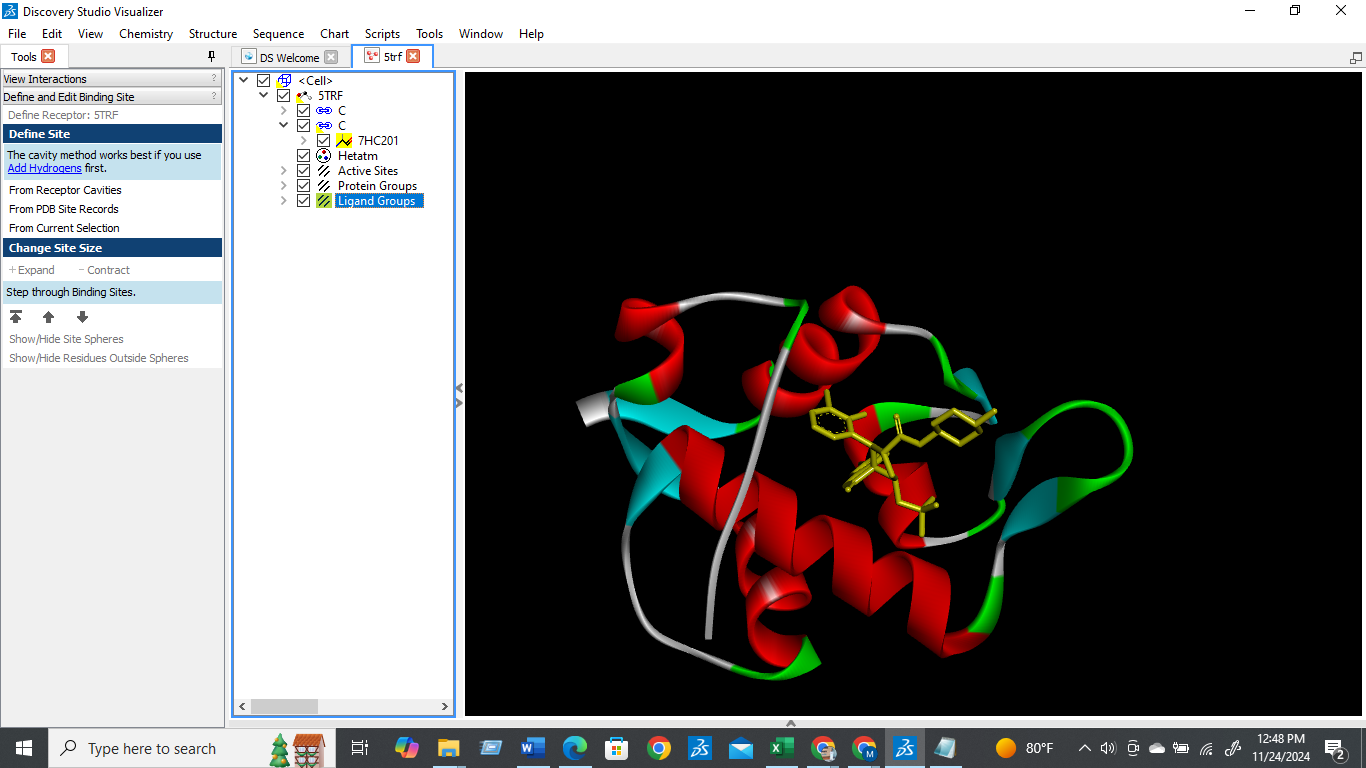


# Then click on the “Current Selection Part” then ligan was encircled with the red sphere.


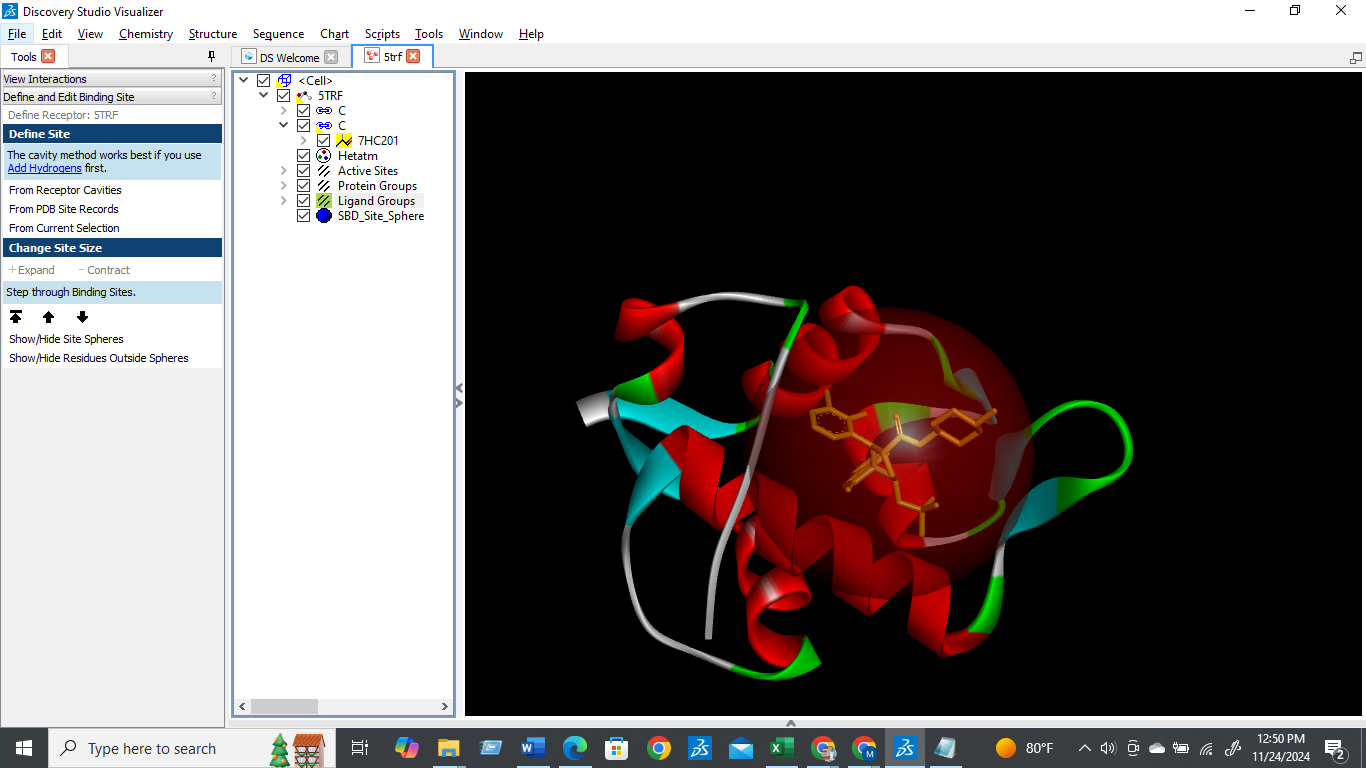


# Then click on the ‘SPDB site sphere & click on the right button. The coordinate values of the ligands was obtained.


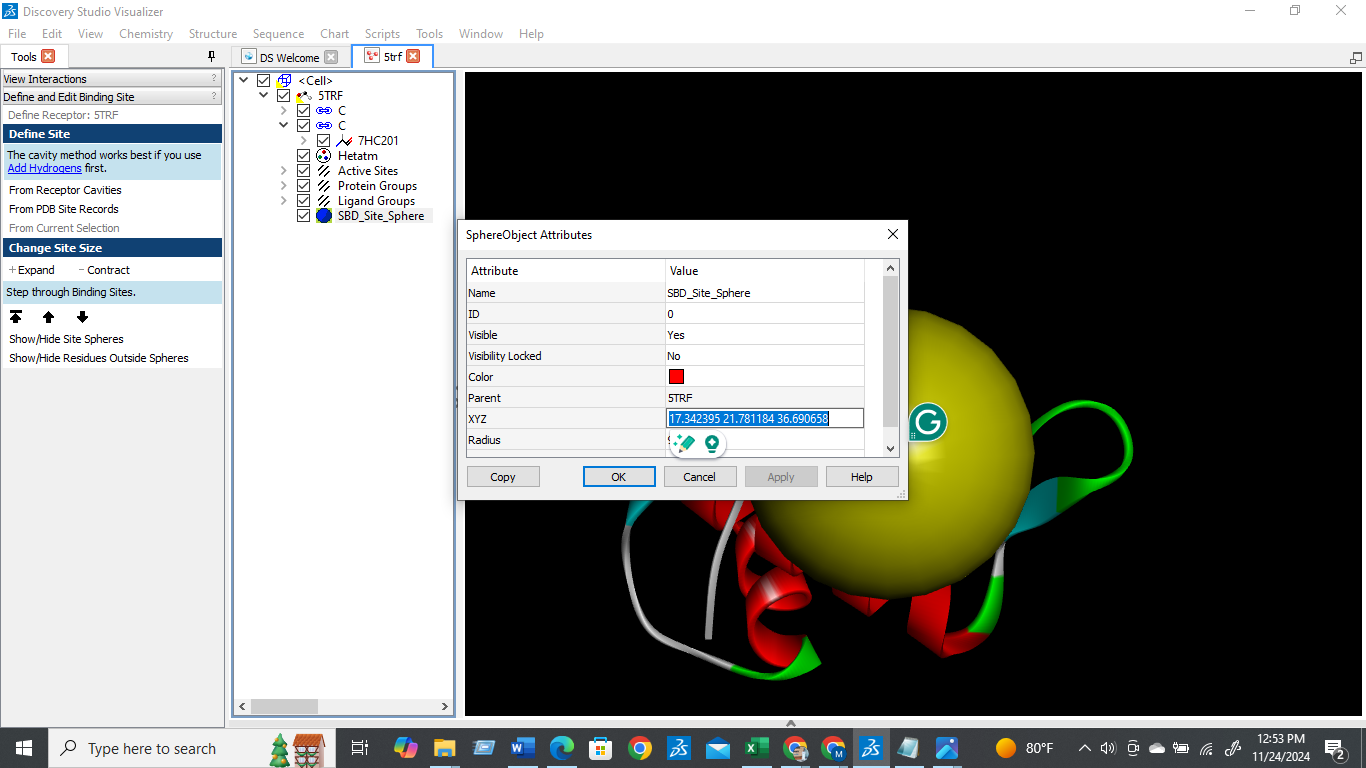


# Following the above procedure, we implemented our theoretical protein-ligand complex and obtained the coordinates value.


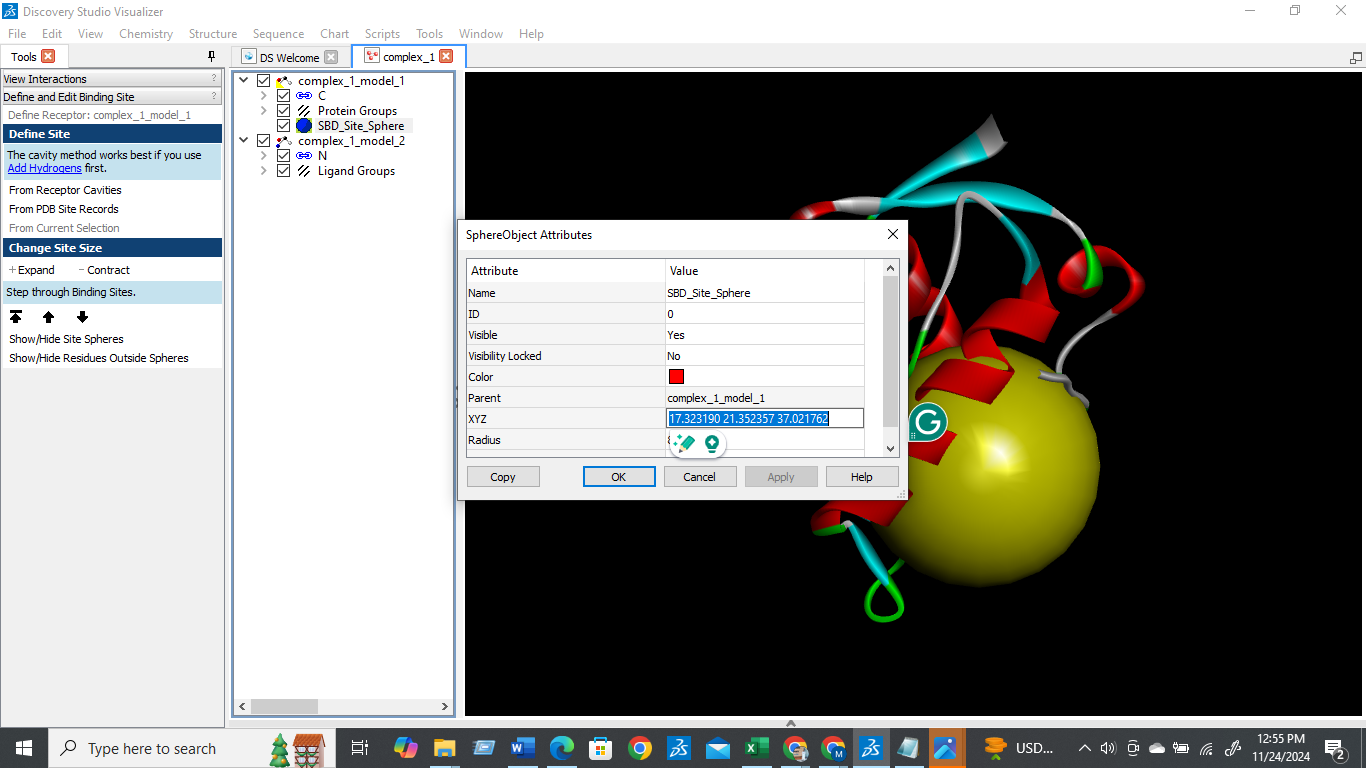

Supplement: S7 File — (DOCX) [file pone.0323003.s007.docx]
