# Supplementary material for: Anti-cancer activity elucidation of geissolosimine as an MDM2-p53 interaction inhibitor: An in-silico study
Source: PLoS One. 2025 May 8;20(5):e0323003. doi: 10.1371/journal.pone.0323003 (PMC12061181; doi:10.1371/journal.pone.0323003)
Supplement: S8 File — (ZIP) [file pone.0323003.s008.zip › Result/complex_2/complex_2_report.html]

YASARA Molecular Dynamics Trajectory Analysis for complex\_2 


# YASARA Molecular Dynamics Trajectory Analysis for complex\_2

## 1. About the simulation

The trajectory **F:\Simulation\26 Arafat\complex\_2\complex\_2** has been analyzed with YASARA version 23.9.29.W.64 over a period of 100.00 nanoseconds with 401 snapshots and the AMBER14 force field. Note that the MD simulation may have been run with a different force field, but AMBER14 was used to calculate the energies in this report. To change this, edit the ForceField setting at the start of this macro.

All plots and pictures in this report [like the simulated system below] are 1024 pixels wide, you can change the **figurewidth** variable in this macro as needed.

|  |
| --- |
|  |
**Figure-1**: A ray-traced picture of the simulated system. The simulation cell boundary is set to periodic. Atoms that stick out of the simulation cell will be wrapped to the opposite side of the cell during the simulation.

### 1.1. Composition of the system

The components of the system are shown in the table below.

|  |  |
| --- | --- |
| **Type** | **Number** |
| Protein molecules | 1 |
| Protein residues | 100 |
| Protein atoms | 1652 |
| Nucleic acid molecules | 0 |
| Nucleic acid residues | 0 |
| Nucleic acid atoms | 0 |
| Residue UNK with 86 atoms | 1 |
| Residue CIM with 1 atom of element Cl | 23 |
| Residue CIP with 1 atom of element Na | 19 |
| Water residues | 7478 |
| Total number of atoms | 24214 |
**Table-1**: Composition of the simulated system

Object 1 with name **complex\_2\_1** has been identified as the solute and is shown below. If this is not the intended solute, please change the **soluteobj** variable in this macro.

|  |
| --- |
|  |
**Figure-2**: The solute oriented along the major axes.

## 2. Analyses inside the simulation cell

This section shows all analyses that have been performed inside the simulation cell, when all atoms share the common coordinate system of the simulation cell.

Periodic boundaries are active and considered for distance measurements. Calculations that involve groups of atoms [center of mass, regression lines, enclosing spheres..] are ambiguous and should be placed in the next section, unless it is known that the atom group does not drift through a periodic boundary.

### 2.1. Simulation cell lengths

Conformational changes of the simulated solute molecules lead to fluctuations in density. If the simulation box has a constant size, changes in density lead to changes in pressure. This is not realistic, because molecules normally "live" in a constant pressure environment. During the simulation the cell is therefore rescaled to maintain a constant cell pressure. Depending on the chosen pressure control mode, the three cell axes are either rescaled together [Manometer1D], partly together [X- and Z-axes, Manometer2D, used for membrane simulations], independently [Manometer3D], or not at all [Off]. You can deduce the pressure control mode from the plot below.

|  |
| --- |
|  |
**Figure-3**: Simulation cell lengths [vertical axis] as a function of simulation time [horizontal axis]. Note: Graph **CellLengthZ** completely covers graph **CellLengthY** and graph **CellLengthX**, they share the same values.

### 2.2. Total potential energy of the system

The total potential energy of the system is plotted, according to the AMBER14 force field. If you ran the simulation with a different force field, you need to adapt the **ForceField** command at the top of this macro accordingly.

When the simulation is started from an energy-minimized "frozen" conformation, there is usually a sharp increase in energy during the first picoseconds, since the added kinetic energy is partly stored as potential energy. Also on a larger time-scale, the potential energy will often not decrease. A common reason are counter ions. These are initially placed at the positions with the lowest potential energy, usually close to charged solute groups, from where they detach to gain entropy, but also potential energy.

|  |
| --- |
|  |
**Figure-4**: Total potential energy of the system [vertical axis] as a function of simulation time [horizontal axis]. Note: The first value of the plot [-403308.90], coming from the energy minimized starting structure, has been replaced with the second value of the plot [-326545.39] to show this plot with a smaller energy range and thus a higher resolution.

### 2.3. Potential energy components

The following individual components of the total potential energy are plotted: bond energies [Bond], bond angle energies [Angle], dihedral angle energies [Dihedral], planarity or improper dihedral energies [Planarity], Van der Waals energies [VdW] and electrostatic energies [Coulomb]. Force field energies help to judge the structural quality of a protein: distortions of local covalent geometry can be found by looking at the bond, angle and planarity energies. Unrealistically close contacts [bumps] lead to a high Van der Waals energy, just like a large number of hydrogen bonds [since they pull the atoms closer than their normal Van der Waals contact distance]. The Coulomb energy is the least informative, because it strongly depends on the amino acid composition [e.g. proteins with a net charge have a higher Coulomb energy].

|  |
| --- |
|  |
**Figure-5**: Potential energy components [vertical axis] as a function of simulation time [horizontal axis].

### 2.4. Surface areas of the solute

The Van der Waals [SurfVdW], molecular [SurfMol] and solvent accessible [SurfAcc] surface areas of the solute in A^2 are plotted. The difference between these surface types can be summarized as follows:

**Van der Waals surface**: if you think of atoms as spheres with a given Van der Waals radius, then the Van der Waals surface consists of all the points on these spheres that are not inside another sphere. In practice, the Van der Waals surface is of limited use, because it can be found throughout a protein and does not tell much about the interaction with the solvent.

**Molecular surface**: this is the Van der Waals surface from the viewpoint of a solvent molecule, which is a much more useful concept. The water is assumed to be a sphere of a given radius [also called the water probe], that rolls over the solute. Those parts of the Van der Waals surface that the water probe can touch are simply copied to the molecular surface [and called the contact surface]. Clefts in the Van der Waals surface that are too narrow for the water probe to enter are replaced by the Van der Waals surface of the water probe itself [and called the reentrant surface]. So the molecular surface is a smooth composition of two Van der Waals surfaces: the one of the solute and the one of the solvent molecule while it traces the contours of the solute. Other common names for the molecular surface are solvent excluded surface or Connolly surface.

**Solvent accessible surface**: this surface consists of all the points that the center of the water probe [i.e. the nucleus of the oxygen atom in the water molecule] can reach while rolling over the solute. The shortest possible distance between the water oxygen nucleus and a solute atom is simply the sum of the Van der Waals radii of the solute atom and the water probe.

|  |
| --- |
|  |
**Figure-6**: Surface areas of the solute [vertical axis] as a function of simulation time [horizontal axis], obtained with the command "SurfObj Solute".

### 2.5. Number of hydrogen bonds in the solute

The number of hydrogen bonds inside the solute is plotted below. One hydrogen bond per hydrogen atom is assigned at most, picking the better one if two acceptors are available.The following formula yields the bond energy in [kJ/mol] as a function of the Hydrogen-Acceptor distance in [A] and two scaling factors:

The first scaling factor depends on the angle formed by Donor-Hydrogen-Acceptor:

The second scaling factor is derived from the angle formed by Hydrogen-Acceptor-X, where X is the atom covalently bound to the acceptor. If X is a heavy atom:

If X is a hydrogen, slightly smaller angles are allowed:

A hydrogen bond is counted if the hydrogen bond energy obtained with this formula is better than 6.25 kJ/mol [or 1.5 kcal/mol], which is 25% of the optimum value 25 kJ/mol.

|  |
| --- |
|  |
**Figure-7**: Number of hydrogen bonds in the solute [vertical axis] as a function of simulation time [horizontal axis].

### 2.6. Number of hydrogen bonds between solute and solvent

The plot shows the number of hydrogen bonds between solute and solvent. Together with the plot above, it is a good indicator for successful protein folding, indicated by a decreasing number of bonds with the solvent and a growing number of bonds within the solute.

|  |
| --- |
|  |
**Figure-8**: Number of hydrogen bonds between solute and solvent [vertical axis] as a function of simulation time [horizontal axis].

### 2.7. Protein secondary structure content

The total percentages of alpha helices, beta sheets, turns, coils, 3-10 helices and pi helices are calculated and plotted. For clarification, a turn is simply a stretch of four residues that are not part of other secondary structure elements and form a hydrogen bond between the O of the first and the NH of the last residue. A coil is anything that does not fit into the other categories. Note that pi-helices [helices with hydrogen bonds between residues N and N+5] are rather unstable and thus do not normally occur in proteins, except for short bulges in alpha helices [which are often the result of single residue insertions and prolines].

|  |
| --- |
|  |
**Figure-9**: Protein secondary structure content [vertical axis] as a function of simulation time [horizontal axis], obtained with the command "SecStr". Note: Graph **HelixPi** has all zero values.

### 2.8. Per-residue protein secondary structure

The following plots show the protein secondary structure per residue as a function of simulation time. They are helpful to monitor protein folding and all other kinds of structural changes. The default secondary structure colors are used, you can change them at View > Color > Parameters > Secondary structure colors. One plot per protein molecule is shown.

|  |
| --- |
|  |
**Figure-10**: Per-residue protein secondary structure as a function of simulation time [horizontal axis] for each Res number [vertical axis]. A table with the raw data including percentages is available here: complex\_2\_plotres\_secstrMolC.tab. Values 1-6 in the table correspond to the 6 labels in the plot legend.

### 2.9. Per-residue number of contacts

The number of contacts per residue as a function of simulation time is shown in the following plots. There is one plot for each protein or nucleic acid molecule. Even though contacts between atoms separated by up to four chemical bonds are excluded, neighboring residues in the molecule usually have enough close atoms to be counted as a contact. Consequently residues with zero contacts are very rare and often glycines. The number of contacts tells you how densely a certain residue range is packed and allows to identify structurally very important residues, e.g. a phenylalanine in the hydrophobic core can contact 15 or more other residues.

|  |
| --- |
|  |
**Figure-11**: Per-residue number of contacts as a function of simulation time [horizontal axis] for each Res number [vertical axis]. A table with the raw data including percentages is available here: complex\_2\_plotres\_conMolC.tab

## 3. Analyses outside the simulation cell

The following section presents data gathered outside the simulation cell, where each object has its own local coordinate system and no periodic boundaries are present. Calculations that involve the interaction between objects [common surface areas, contacts between objects..] must be placed in the previous section.

### 3.1. Radius of gyration of the solute

After determining the center of mass of the solute, the radius of gyration is calculated and plotted according to this formula:

In this formula, **C** is the center of mass, and **Ri** is the position of atom **i** of **N**.

|  |
| --- |
|  |
**Figure-12**: Radius of gyration of the solute [vertical axis] as a function of simulation time [horizontal axis], obtained with the command "RadiusObj Solute,Center=Mass,Type=Gyration".

## 4. Analyses performed with respect to the starting structure

Analyses performed with respect to the starting structure are shown in this section. These are also done outside the simulation cell, where each object has its own local coordinate systems and no periodic boundaries are present. To choose another reference snapshot than 0, edit the **refsnapshot** variable at the beginning of this macro.

### 4.1. Solute RMSD from the starting structure

The plot shows Calpha [RMSDCa], backbone [RMSDBb] and all-heavy atom [RMSDAll] RMSDs calculated according to this formula, where **Ri** is the vector linking the positions of atom **i** [of **N** atoms] in the reference snapshot and the current snapshot after optimal superposition:

The selection for the Calpha RMSD calculation is **CA Protein or C1\* NucAcid and Obj Solute**, this matched 100 atoms. The Calpha selection thus includes the main backbone carbon C1\* of nucleic acids, so the plot also shows a Calpha RMSD if you simulate just nucleic acids. In simulations of protein-DNA complexes, the Calpha RMSD therefore considers the DNA too. To change the Calpha selection, edit the **casel** variable at the beginning of this macro.

|  |
| --- |
|  |
**Figure-13**: Solute RMSD from the starting structure [vertical axis] as a function of simulation time [horizontal axis].

## 5. Solute residue RMSF

The Root Mean Square Fluctuation [RMSF] per solute residue is calculated from the average RMSF of its constituting atoms. The RMSF of atom i with j runing from 1 to 3 for the x, y, and z coordinate of the atom position vector P and k runing over the set of N evaluated snapshots is given by following formula:

Each graph in the following plot represents one molecule, so that you can easily see differences between molecules. Note: Residue numbers are not unique, so graphs can overlap.

|  |
| --- |
|  |
**Figure-14**: The Root Mean Square Fluctuation [vertical axis] per solute protein/nucleic acid residue [horizontal axis] calculated from the average RMSF of the atoms constituting the residue. A RMSF of exactly zero means that that residue number is not present in the molecule. Atom RMSF table: complex\_2\_rmsf.tab, residue RMSF table: complex\_2\_rmsfres.tab

## 6. Dynamic Cross-Correlation Matrix

The dynamic cross-correlation matrix [DCCM] is a square matrix, whose rows and columns match the selected units **Atom CA Protein or C1\* NucAcid**. To change this selection, edit the **dccmsel** variable at the beginning of this macro. The DCCM shows how the movements of all selected pairs correlate. The values in the DCCM range from -1 [perfectly anti-correlated] to +1 [perfectly correlated]. The values along the diagonal are always +1 [because the motion of an atom is perfectly correlated to itself]. The DCCM element for units i and j is obtained with the following formula:

Here **d** is the displacement between the current position and the average position of the selected unit, and the angle brackets indicate the average over all samples. The highest correlations off the diagonal can often be found for bridged cysteines.

The image below shows the correlation directly in the solute object:

|  |
| --- |
|  |
**Figure-15**: Blue and red lines are shown between 0 strongly anti- and correlated residue pairs. To change the threshold value for the correlation lines edit the **dccmcut** variable at the beginning of this macro. To look at this structure interactively, open the file complex\_2\_dccm.yob in YASARA.

In the image below, the DCCM is visualized with colors ranging from blue [-1, fully anti-correlated] to yellow [+1, fully correlated].

|  |
| --- |
|  |
**Figure-16**: Visualization of the dynamic cross-correlation matrix. Open the file complex\_2\_dccm.sce in YASARA to look at this matrix visualization interactively. In the scene file, the zero level [0, not correlated] is indicated with a wire-frame grid.

|  |  |  |  |  |  |  |  |  |  |  |  |  |  |  |  |  |  |  |  |  |  |  |  |  |  |  |  |  |  |  |  |  |  |  |  |  |  |  |  |  |  |  |  |  |  |  |  |  |  |  |  |  |  |  |  |  |  |  |  |  |  |  |  |  |  |  |  |  |  |  |  |  |  |  |  |  |  |  |  |  |  |  |  |  |  |  |  |  |  |  |  |  |  |  |  |  |  |  |  |  |
| --- | --- | --- | --- | --- | --- | --- | --- | --- | --- | --- | --- | --- | --- | --- | --- | --- | --- | --- | --- | --- | --- | --- | --- | --- | --- | --- | --- | --- | --- | --- | --- | --- | --- | --- | --- | --- | --- | --- | --- | --- | --- | --- | --- | --- | --- | --- | --- | --- | --- | --- | --- | --- | --- | --- | --- | --- | --- | --- | --- | --- | --- | --- | --- | --- | --- | --- | --- | --- | --- | --- | --- | --- | --- | --- | --- | --- | --- | --- | --- | --- | --- | --- | --- | --- | --- | --- | --- | --- | --- | --- | --- | --- | --- | --- | --- | --- | --- | --- | --- | --- |
| DCCM | C  Asp  11 | C  Gly  12 | C  Ala  13 | C  Val  14 | C  Thr  15 | C  Thr  16 | C  Ser  17 | C  Gln  18 | C  Ile  19 | C  Pro  20 | C  Ala  21 | C  Ser  22 | C  Glu  23 | C  Gln  24 | C  Glu  25 | C  Thr  26 | C  Leu  27 | C  Val  28 | C  Arg  29 | C  Pro  30 | C  Lys  31 | C  Pro  32 | C  Leu  33 | C  Leu  34 | C  Leu  35 | C  Lys  36 | C  Leu  37 | C  Leu  38 | C  Lys  39 | C  Ser  40 | C  Val  41 | C  Gly  42 | C  Ala  43 | C  Gln  44 | C  Lys  45 | C  Asp  46 | C  Thr  47 | C  Tyr  48 | C  Thr  49 | C  Met  50 | C  Lys  51 | C  Glu  52 | C  Val  53 | C  Leu  54 | C  Phe  55 | C  Tyr  56 | C  Leu  57 | C  Gly  58 | C  Gln  59 | C  Tyr  60 | C  Ile  61 | C  Met  62 | C  Thr  63 | C  Lys  64 | C  Arg  65 | C  Leu  66 | C  Tyr  67 | C  Asp  68 | C  Glu  69 | C  Lys  70 | C  Gln  71 | C  Gln  72 | C  His  73 | C  Ile  74 | C  Val  75 | C  Tyr  76 | C  Cys  77 | C  Ser  78 | C  Asn  79 | C  Asp  80 | C  Leu  81 | C  Leu  82 | C  Gly  83 | C  Asp  84 | C  Leu  85 | C  Phe  86 | C  Gly  87 | C  Val  88 | C  Pro  89 | C  Ser  90 | C  Phe  91 | C  Ser  92 | C  Val  93 | C  Lys  94 | C  Glu  95 | C  His  96 | C  Arg  97 | C  Lys  98 | C  Ile  99 | C  Tyr  100 | C  Thr  101 | C  Met  102 | C  Ile  103 | C  Tyr  104 | C  Arg  105 | C  Asn  106 | C  Leu  107 | C  Val  108 | C  Val  109 | C  Val  110 |
| C-Asp-11 | 1.00 | 0.50 | 0.24 | -0.07 | -0.03 | -0.02 | -0.04 | -0.05 | -0.07 | -0.04 | -0.03 | -0.07 | -0.02 | -0.02 | -0.10 | -0.07 | -0.07 | -0.08 | -0.05 | -0.12 | -0.11 | -0.04 | -0.01 | -0.06 | -0.13 | -0.05 | -0.04 | -0.16 | -0.15 | -0.09 | -0.10 | -0.18 | -0.07 | -0.02 | 0.03 | -0.03 | -0.03 | -0.10 | -0.10 | -0.05 | -0.07 | 0.02 | -0.15 | -0.15 | -0.07 | -0.13 | -0.14 | -0.13 | -0.18 | -0.18 | -0.12 | -0.11 | -0.10 | -0.08 | -0.07 | -0.11 | -0.13 | -0.07 | -0.15 | -0.04 | 0.09 | 0.01 | 0.02 | 0.07 | -0.09 | 0.03 | -0.01 | 0.07 | 0.04 | -0.02 | -0.01 | 0.01 | 0.03 | 0.01 | 0.01 | 0.00 | 0.04 | 0.08 | 0.10 | 0.09 | 0.05 | 0.02 | -0.08 | -0.05 | 0.00 | -0.05 | -0.06 | -0.00 | -0.02 | -0.04 | -0.02 | -0.04 | -0.04 | -0.05 | -0.06 | -0.07 | -0.06 | -0.03 | -0.03 | -0.01 |
| C-Gly-12 | 0.50 | 1.00 | 0.66 | 0.21 | 0.09 | 0.16 | 0.10 | 0.05 | -0.01 | -0.04 | -0.03 | -0.06 | -0.01 | 0.09 | 0.02 | -0.07 | -0.03 | -0.05 | -0.03 | -0.15 | -0.10 | -0.06 | -0.01 | -0.04 | -0.14 | -0.04 | -0.04 | -0.14 | -0.12 | -0.06 | -0.06 | -0.17 | -0.03 | -0.01 | 0.07 | -0.09 | -0.09 | -0.03 | 0.02 | 0.09 | 0.16 | 0.25 | -0.09 | -0.09 | 0.11 | -0.00 | -0.06 | -0.02 | -0.04 | -0.10 | -0.07 | -0.02 | -0.03 | -0.07 | -0.05 | -0.05 | -0.11 | 0.00 | -0.23 | -0.08 | -0.02 | -0.18 | -0.18 | -0.06 | -0.05 | 0.02 | -0.01 | 0.11 | 0.03 | 0.01 | 0.01 | 0.01 | 0.01 | 0.01 | -0.00 | 0.03 | 0.04 | 0.08 | 0.09 | 0.11 | 0.08 | -0.09 | -0.18 | -0.09 | -0.09 | -0.05 | -0.07 | -0.04 | -0.06 | -0.07 | -0.09 | -0.10 | -0.08 | -0.12 | -0.10 | -0.05 | -0.06 | -0.02 | -0.01 | -0.04 |
| C-Ala-13 | 0.24 | 0.66 | 1.00 | 0.56 | 0.35 | 0.34 | 0.20 | 0.11 | 0.02 | -0.02 | 0.04 | -0.01 | 0.05 | 0.22 | 0.11 | 0.02 | 0.02 | -0.04 | -0.05 | -0.19 | -0.11 | -0.09 | -0.01 | -0.02 | -0.17 | -0.03 | -0.01 | -0.15 | -0.14 | -0.03 | -0.00 | -0.14 | -0.02 | -0.06 | 0.06 | -0.15 | -0.09 | 0.03 | 0.14 | 0.25 | 0.41 | 0.36 | -0.02 | 0.05 | 0.19 | 0.06 | 0.03 | 0.05 | 0.06 | 0.02 | 0.02 | 0.04 | 0.07 | -0.00 | 0.02 | 0.01 | -0.01 | 0.05 | -0.21 | -0.10 | -0.10 | -0.35 | -0.35 | -0.17 | -0.06 | 0.00 | 0.02 | 0.11 | 0.03 | 0.03 | 0.03 | 0.02 | 0.01 | 0.01 | -0.01 | 0.00 | 0.03 | 0.07 | 0.06 | 0.07 | 0.01 | -0.23 | -0.28 | -0.17 | -0.17 | -0.08 | -0.07 | -0.09 | -0.07 | -0.09 | -0.12 | -0.12 | -0.09 | -0.16 | -0.11 | -0.07 | -0.10 | -0.05 | -0.01 | -0.02 |
| C-Val-14 | -0.07 | 0.21 | 0.56 | 1.00 | 0.59 | 0.39 | 0.24 | 0.14 | 0.06 | -0.02 | 0.08 | -0.01 | 0.02 | 0.17 | 0.14 | 0.05 | -0.01 | -0.06 | -0.07 | -0.11 | -0.02 | 0.04 | 0.07 | 0.07 | 0.01 | 0.10 | 0.11 | 0.02 | 0.02 | 0.13 | 0.14 | 0.01 | 0.07 | -0.16 | -0.09 | -0.20 | -0.13 | 0.00 | 0.12 | 0.25 | 0.50 | 0.28 | 0.07 | 0.24 | 0.26 | 0.21 | 0.15 | 0.11 | 0.13 | 0.13 | 0.07 | 0.01 | 0.02 | -0.01 | -0.09 | -0.03 | 0.06 | 0.09 | 0.02 | -0.12 | -0.33 | -0.48 | -0.45 | -0.39 | -0.13 | -0.08 | 0.02 | 0.05 | 0.05 | 0.08 | 0.08 | 0.07 | 0.05 | 0.07 | 0.03 | 0.02 | 0.04 | -0.03 | -0.06 | -0.06 | -0.12 | -0.34 | -0.32 | -0.23 | -0.21 | -0.11 | -0.00 | -0.04 | -0.06 | -0.04 | -0.04 | -0.06 | -0.04 | -0.12 | -0.09 | -0.08 | -0.08 | -0.08 | -0.05 | -0.03 |
| C-Thr-15 | -0.03 | 0.09 | 0.35 | 0.59 | 1.00 | 0.59 | 0.36 | 0.14 | 0.09 | 0.09 | 0.14 | 0.07 | 0.10 | 0.25 | 0.10 | -0.01 | -0.05 | -0.09 | -0.11 | -0.11 | -0.09 | -0.08 | 0.00 | 0.05 | -0.06 | -0.03 | 0.06 | 0.01 | -0.07 | -0.02 | -0.00 | -0.06 | -0.05 | -0.12 | -0.03 | -0.09 | -0.09 | -0.03 | 0.05 | 0.10 | 0.25 | 0.14 | -0.03 | 0.12 | 0.12 | 0.09 | 0.07 | 0.04 | 0.09 | 0.11 | 0.05 | 0.04 | 0.11 | 0.11 | 0.00 | 0.03 | -0.06 | -0.16 | -0.15 | -0.24 | -0.23 | -0.23 | -0.23 | -0.20 | -0.08 | 0.01 | 0.09 | 0.10 | 0.06 | 0.09 | 0.09 | 0.06 | 0.06 | 0.10 | 0.05 | 0.04 | 0.06 | 0.04 | 0.03 | 0.04 | -0.01 | -0.11 | -0.15 | -0.10 | -0.12 | -0.02 | -0.01 | 0.01 | 0.02 | 0.00 | -0.03 | -0.01 | -0.02 | -0.10 | -0.05 | -0.09 | -0.13 | -0.15 | -0.13 | -0.08 |
| C-Thr-16 | -0.02 | 0.16 | 0.34 | 0.39 | 0.59 | 1.00 | 0.74 | 0.54 | 0.37 | 0.18 | 0.17 | 0.04 | 0.09 | 0.25 | 0.06 | -0.04 | -0.12 | -0.17 | -0.15 | -0.14 | -0.09 | -0.07 | 0.00 | 0.04 | -0.09 | -0.04 | 0.02 | -0.01 | -0.07 | -0.02 | -0.01 | -0.08 | -0.05 | 0.02 | 0.05 | -0.07 | -0.10 | -0.08 | -0.01 | 0.05 | 0.15 | 0.09 | -0.09 | -0.00 | 0.03 | -0.03 | 0.00 | 0.00 | 0.04 | 0.03 | 0.02 | 0.06 | 0.09 | 0.09 | 0.03 | 0.00 | -0.17 | -0.16 | -0.26 | -0.20 | -0.19 | -0.21 | -0.22 | -0.19 | -0.10 | -0.05 | 0.02 | 0.05 | 0.05 | 0.05 | 0.06 | -0.03 | -0.05 | 0.05 | 0.02 | -0.04 | -0.00 | -0.01 | 0.01 | 0.02 | -0.02 | -0.13 | -0.12 | -0.03 | 0.01 | 0.16 | 0.13 | 0.10 | 0.08 | 0.07 | 0.02 | 0.01 | 0.00 | -0.02 | -0.02 | -0.05 | -0.15 | -0.17 | -0.15 | -0.13 |
| C-Ser-17 | -0.04 | 0.10 | 0.20 | 0.24 | 0.36 | 0.74 | 1.00 | 0.66 | 0.43 | 0.29 | 0.23 | 0.10 | 0.11 | 0.20 | 0.05 | -0.05 | -0.11 | -0.15 | -0.10 | -0.11 | -0.10 | -0.09 | -0.02 | 0.03 | -0.11 | -0.07 | -0.01 | -0.01 | -0.07 | -0.03 | 0.01 | -0.06 | -0.07 | 0.04 | 0.05 | -0.01 | -0.06 | -0.08 | -0.03 | 0.04 | 0.07 | 0.03 | -0.08 | -0.02 | 0.01 | -0.02 | 0.00 | 0.01 | 0.04 | 0.03 | 0.04 | 0.07 | 0.09 | 0.08 | 0.05 | 0.02 | -0.19 | -0.13 | -0.26 | -0.16 | -0.13 | -0.17 | -0.17 | -0.18 | -0.10 | -0.03 | 0.01 | 0.04 | 0.03 | 0.04 | 0.05 | -0.05 | -0.07 | 0.05 | -0.00 | -0.07 | -0.04 | -0.05 | -0.03 | -0.02 | -0.08 | -0.18 | -0.11 | -0.05 | -0.01 | 0.15 | 0.11 | 0.07 | 0.09 | 0.08 | 0.04 | 0.03 | 0.04 | 0.02 | 0.01 | -0.05 | -0.13 | -0.14 | -0.13 | -0.13 |
| C-Gln-18 | -0.05 | 0.05 | 0.11 | 0.14 | 0.14 | 0.54 | 0.66 | 1.00 | 0.71 | 0.38 | 0.22 | 0.11 | 0.15 | 0.16 | -0.02 | -0.10 | -0.18 | -0.18 | -0.12 | -0.07 | -0.01 | -0.05 | 0.02 | 0.08 | -0.08 | -0.08 | -0.01 | -0.00 | -0.09 | -0.05 | 0.01 | -0.05 | -0.08 | 0.03 | 0.03 | -0.00 | -0.11 | -0.15 | -0.10 | 0.03 | 0.02 | -0.02 | -0.07 | -0.03 | 0.02 | -0.02 | 0.01 | 0.03 | 0.04 | 0.03 | 0.05 | 0.07 | 0.07 | 0.10 | 0.08 | 0.06 | -0.14 | -0.11 | -0.27 | -0.17 | -0.14 | -0.15 | -0.13 | -0.21 | -0.08 | -0.10 | -0.07 | -0.04 | -0.00 | -0.00 | 0.02 | -0.07 | -0.11 | 0.01 | -0.01 | -0.08 | -0.09 | -0.10 | -0.09 | -0.11 | -0.16 | -0.19 | -0.01 | 0.07 | 0.15 | 0.36 | 0.31 | 0.23 | 0.25 | 0.23 | 0.16 | 0.10 | 0.08 | 0.07 | 0.02 | -0.00 | -0.12 | -0.11 | -0.12 | -0.18 |
| C-Ile-19 | -0.07 | -0.01 | 0.02 | 0.06 | 0.09 | 0.37 | 0.43 | 0.71 | 1.00 | 0.66 | 0.37 | 0.28 | 0.37 | 0.31 | 0.08 | -0.01 | -0.13 | -0.16 | -0.13 | -0.11 | -0.08 | -0.09 | -0.06 | -0.00 | -0.11 | -0.11 | -0.05 | -0.03 | -0.11 | -0.07 | 0.00 | -0.02 | -0.06 | 0.00 | 0.01 | 0.00 | -0.11 | -0.13 | -0.06 | 0.08 | 0.05 | 0.01 | -0.07 | -0.04 | -0.00 | -0.01 | 0.00 | 0.00 | 0.02 | 0.02 | 0.01 | 0.04 | 0.07 | 0.09 | 0.07 | 0.04 | -0.12 | -0.05 | -0.20 | -0.09 | -0.06 | -0.09 | -0.11 | -0.19 | -0.13 | -0.16 | -0.12 | -0.08 | -0.03 | -0.02 | 0.00 | -0.08 | -0.11 | 0.00 | -0.02 | -0.10 | -0.11 | -0.14 | -0.17 | -0.17 | -0.24 | -0.27 | -0.05 | -0.02 | 0.17 | 0.30 | 0.32 | 0.22 | 0.17 | 0.24 | 0.16 | 0.06 | 0.07 | 0.08 | 0.06 | -0.01 | -0.15 | -0.11 | -0.11 | -0.16 |
**Table-2**: Dynamic cross-correlation matrix. The full table is also available in text format, you need a proper text editor without line wrapping to look at this file: complex\_2\_dccm.tab. Note: At most 10 rows of the DCCM are shown above. Change the **tabrowsmax** variable in the macro to adjust this number.

## 7. Additional files

The following additional files have been created:

### 7.1. The main data table

The main table contains all collected data in a single file. The column names match the names used above for graphs in plots and columns in tables. You can find a more detailed explanation of this table in the user manual at Recipes > Run a molecular dynamics simulation > Analyzing a trajectory. If you parse this file automatically, keep in mind that the number of columns can change any time, so you have to use the names in the first table row to find the columns of interest: complex\_2\_analysis.tab

### 7.2. Per-atom and per-residue data tables

Data of the per-atom and per-residue plots:

complex\_2\_plotres\_secstrMolC.tab

complex\_2\_plotres\_conMolC.tab

### 7.3. The structures

The **time averaged structure** in PDB format: complex\_2\_average.pdb

The **snapshot with the minimum solute energy**. Either just the solute in PDB format complex\_2\_energymin.pdb, or the complete system including solvent as a YASARA scene complex\_2\_energymin.sce.

The **last snapshot** of the simulation. Either just the solute in PDB format complex\_2\_last.pdb, or the complete system including solvent as a YASARA scene complex\_2\_last.sce

### 7.4. The RMSF tables

A table that lists the Root Mean Square Fluctuations [RMSFs] of all atoms in [A] is available here: complex\_2\_rmsf.tab. The RMSFs have also been converted to B-factors and stored in the B-factor field of the time-average structure above.

A table with average atom RMSFs per residue can be found here: complex\_2\_rmsfres.tab.

### 7.5. High resolution plots

To facilitate publication, high resolution versions of the plots above have been created with a 4:3 aspect ratio suited for printing in a single column of a typical journal article. Just look at the figure number above to find the right file:

complex\_2\_report\_figure3\_hires.png

complex\_2\_report\_figure4\_hires.png

complex\_2\_report\_figure5\_hires.png

complex\_2\_report\_figure6\_hires.png

complex\_2\_report\_figure7\_hires.png

complex\_2\_report\_figure8\_hires.png

complex\_2\_report\_figure9\_hires.png

complex\_2\_report\_figure10\_hires.png

complex\_2\_report\_figure11\_hires.png

complex\_2\_report\_figure12\_hires.png

complex\_2\_report\_figure13\_hires.png

complex\_2\_report\_figure14\_hires.png
