# Supplementary figures and images for: Anti-cancer activity elucidation of geissolosimine as an MDM2-p53 interaction inhibitor: An in-silico study
Source: PLoS One. 2025 May 8;20(5):e0323003. doi: 10.1371/journal.pone.0323003 (PMC12061181; doi:10.1371/journal.pone.0323003)

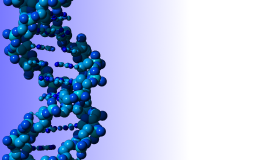

Supplement: S8 File — (ZIP) [file pone.0323003.s008.zip › Result/complex_1/complex_1_report_background.png]

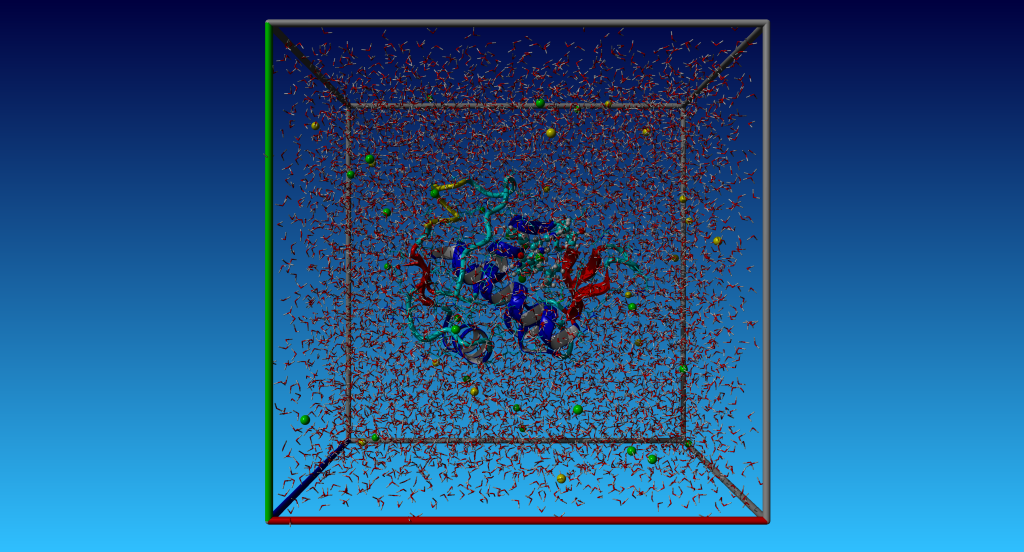

Supplement: S8 File — (ZIP) [file pone.0323003.s008.zip › Result/complex_1/complex_1_report_figure1.png]

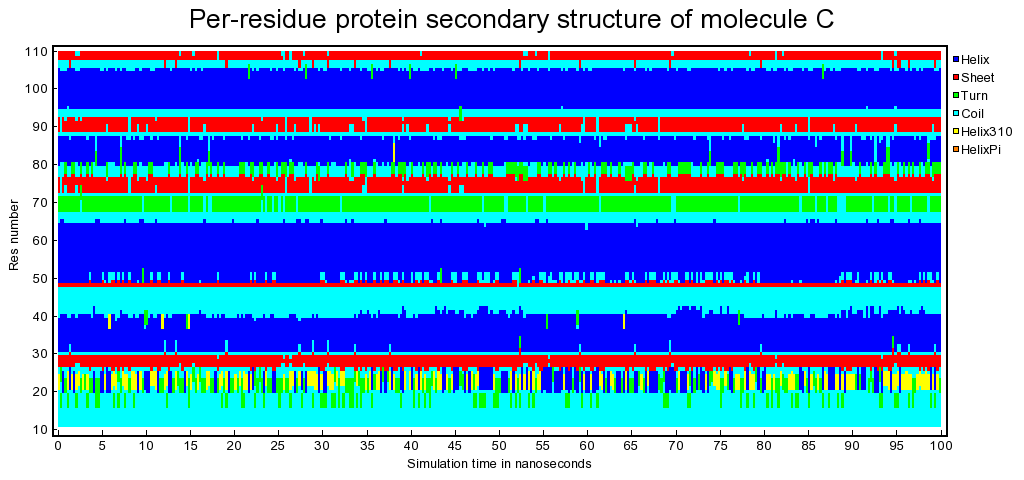

Supplement: S8 File — (ZIP) [file pone.0323003.s008.zip › Result/complex_1/complex_1_report_figure10.png]

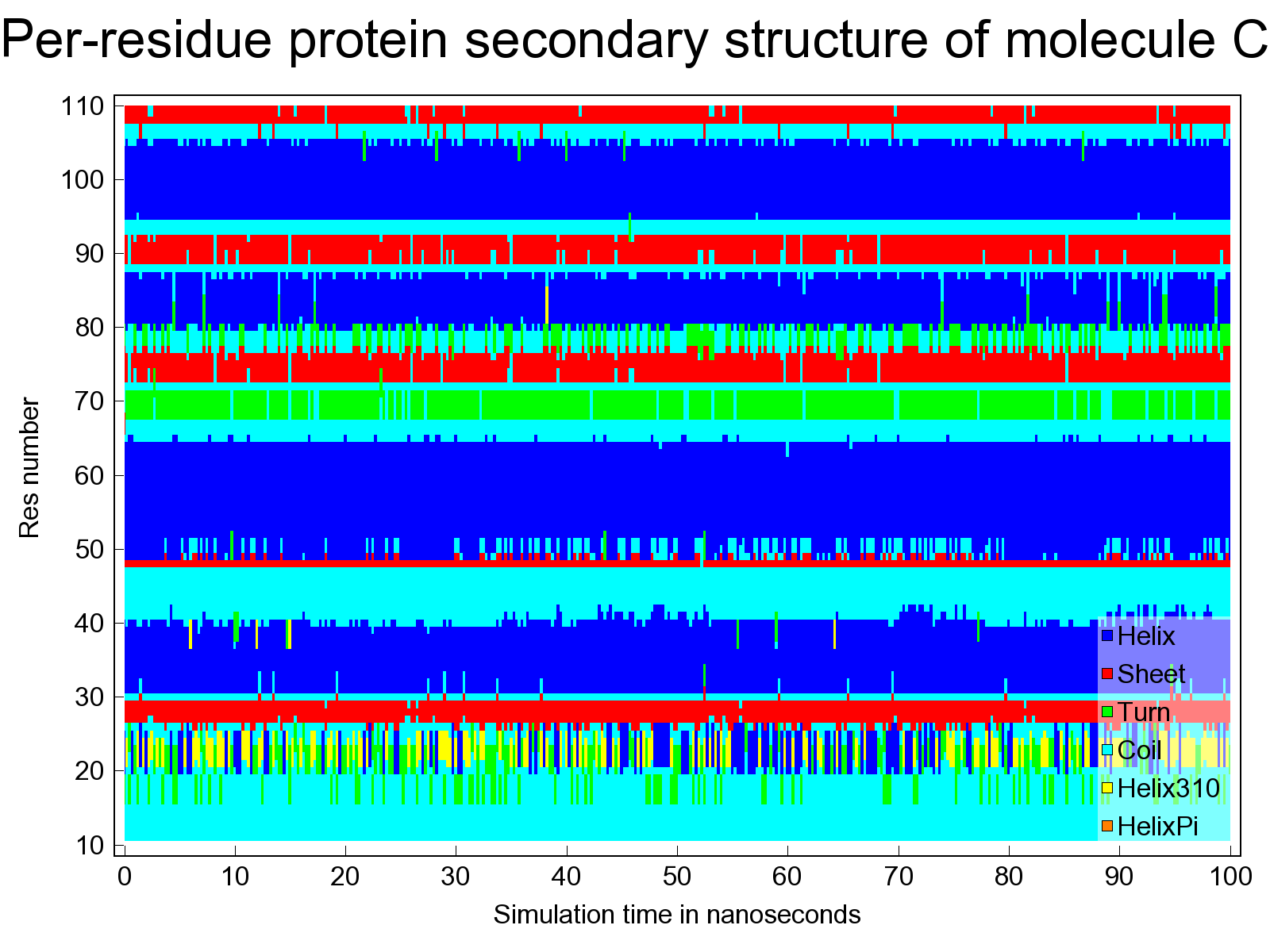

Supplement: S8 File — (ZIP) [file pone.0323003.s008.zip › Result/complex_1/complex_1_report_figure10_hires.png]

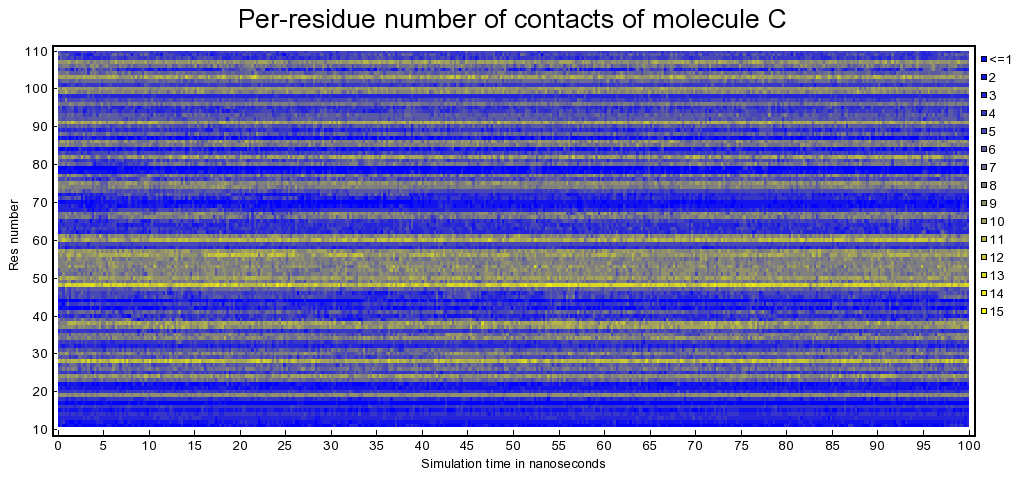

Supplement: S8 File — (ZIP) [file pone.0323003.s008.zip › Result/complex_1/complex_1_report_figure11.png]

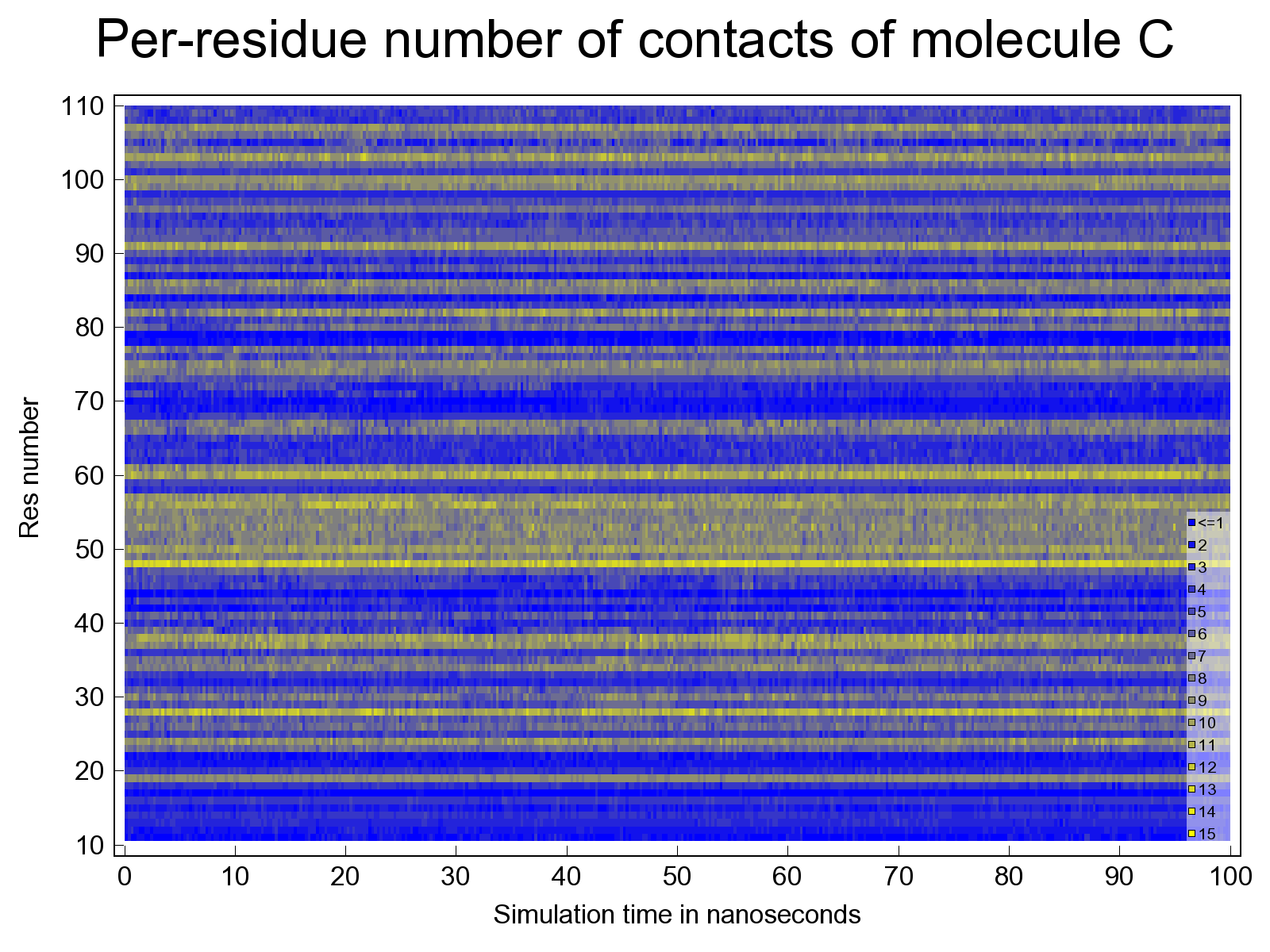

Supplement: S8 File — (ZIP) [file pone.0323003.s008.zip › Result/complex_1/complex_1_report_figure11_hires.png]

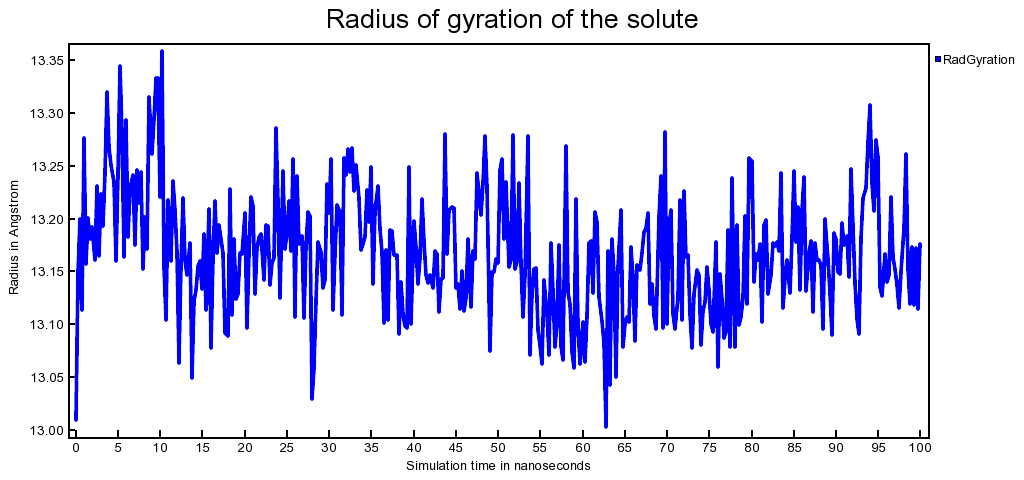

Supplement: S8 File — (ZIP) [file pone.0323003.s008.zip › Result/complex_1/complex_1_report_figure12.png]

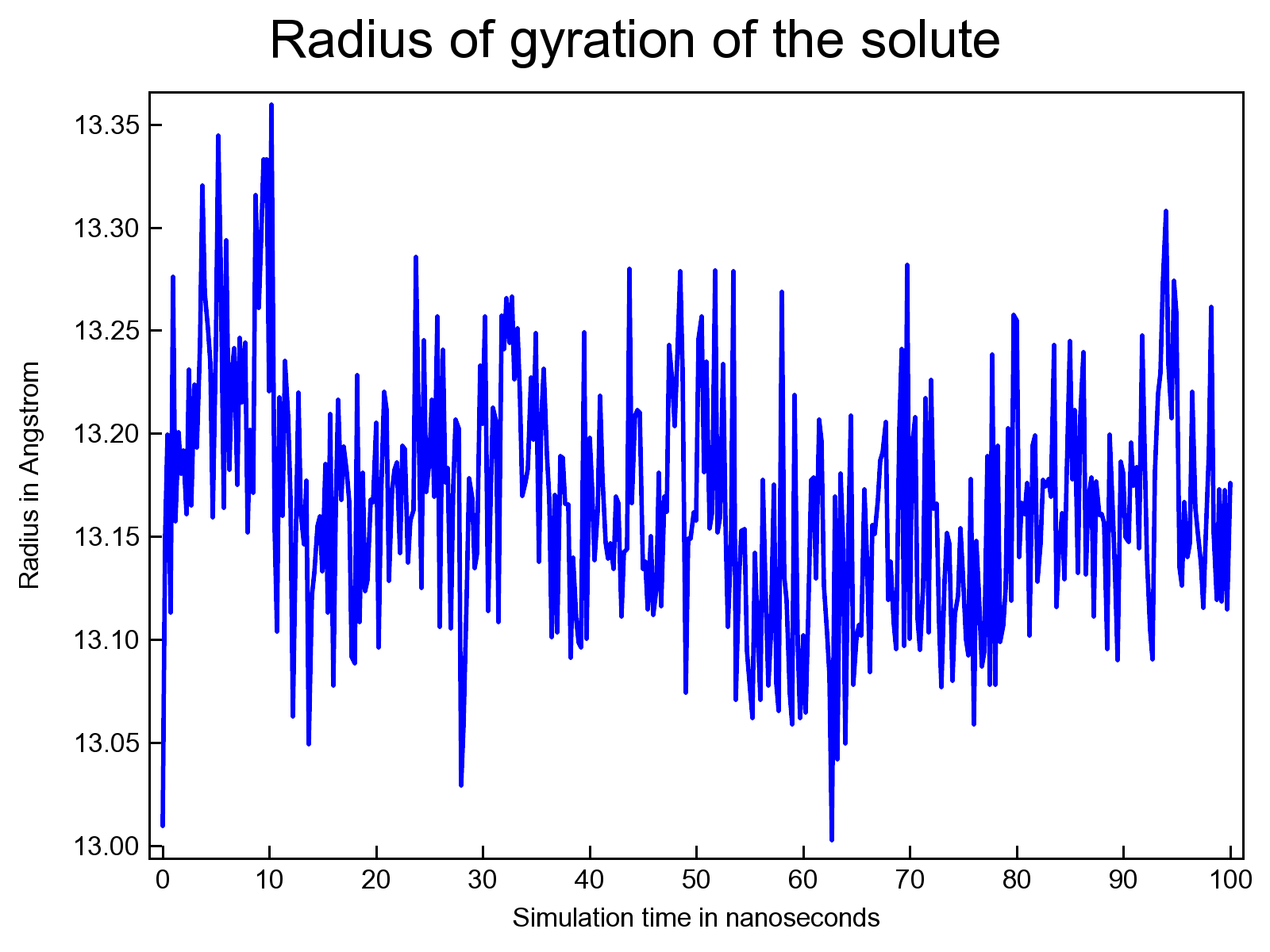

Supplement: S8 File — (ZIP) [file pone.0323003.s008.zip › Result/complex_1/complex_1_report_figure12_hires.png]

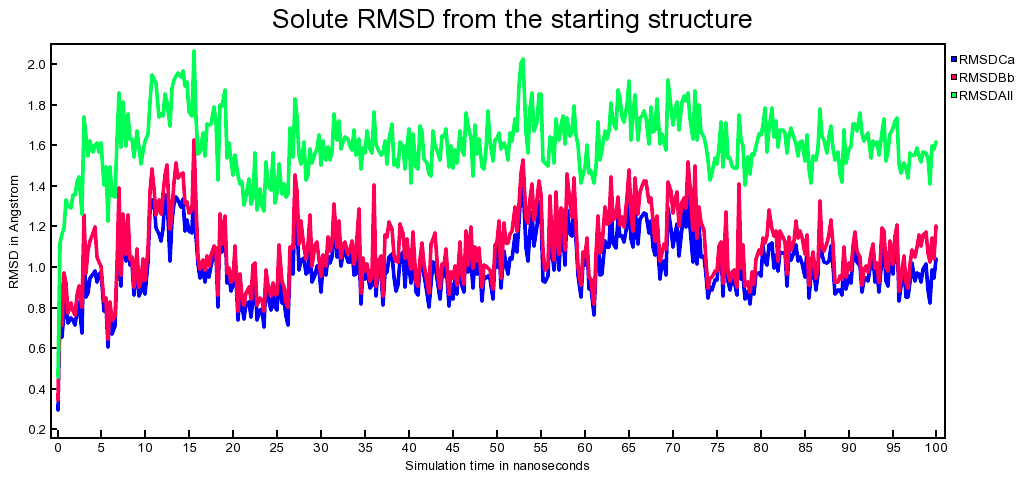

Supplement: S8 File — (ZIP) [file pone.0323003.s008.zip › Result/complex_1/complex_1_report_figure13.png]

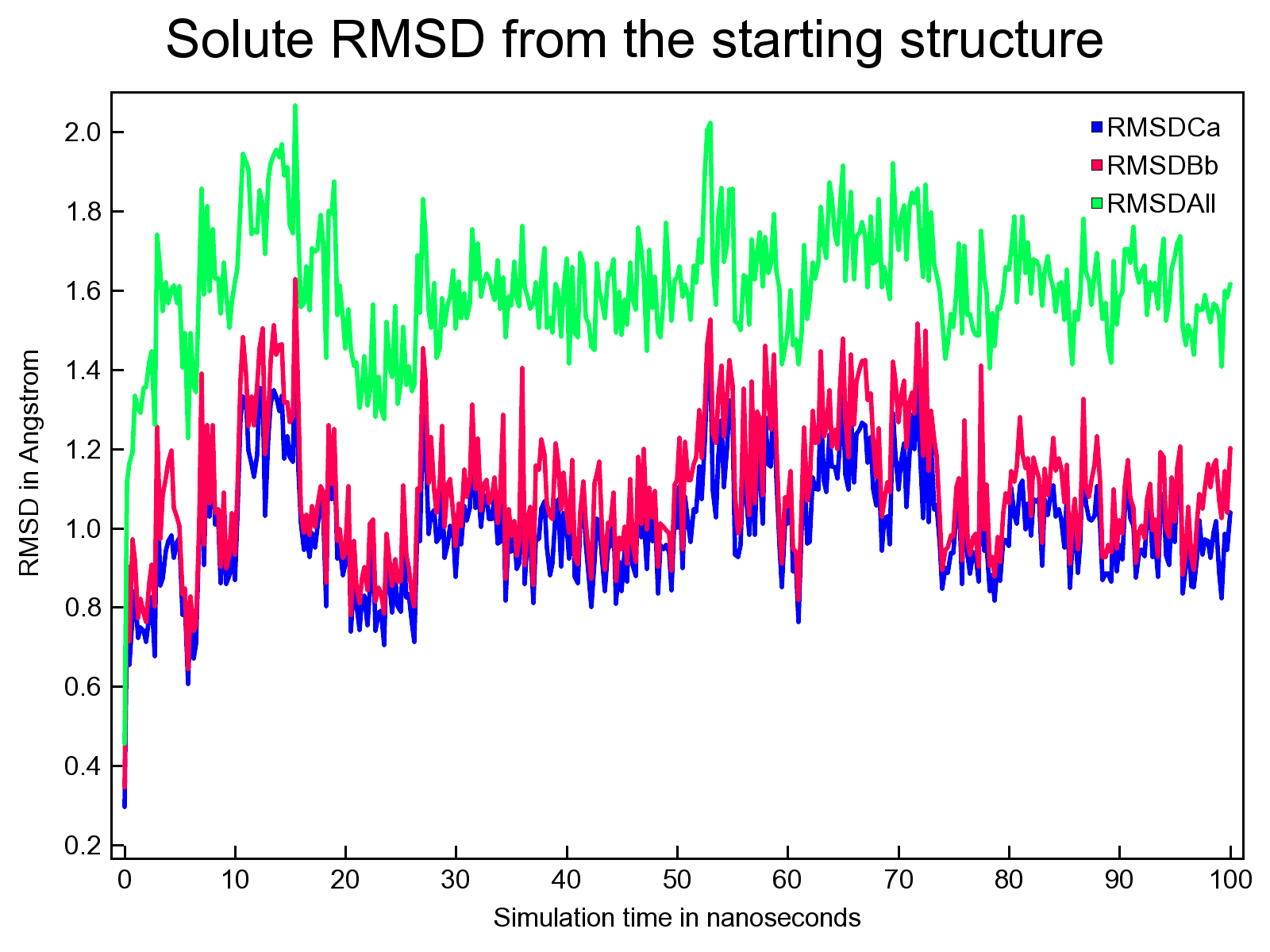

Supplement: S8 File — (ZIP) [file pone.0323003.s008.zip › Result/complex_1/complex_1_report_figure13_hires.png]

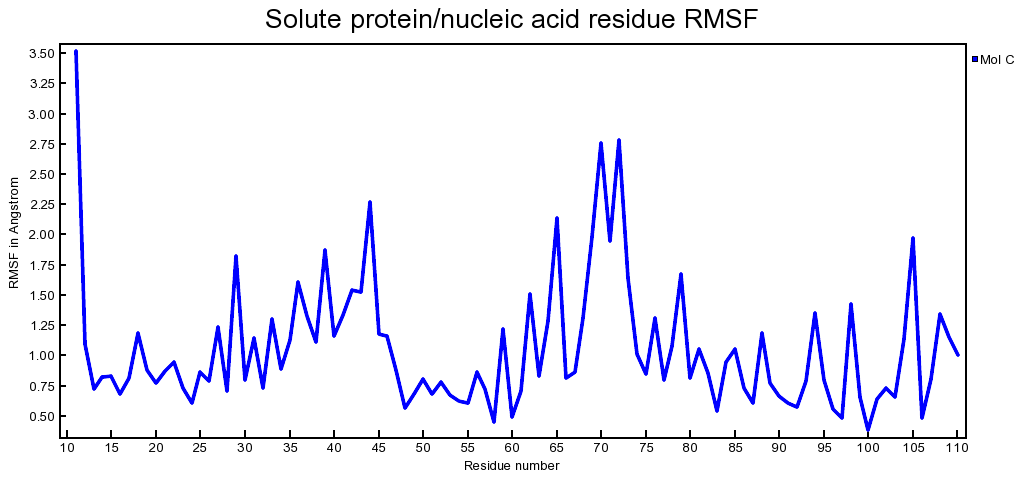

Supplement: S8 File — (ZIP) [file pone.0323003.s008.zip › Result/complex_1/complex_1_report_figure14.png]

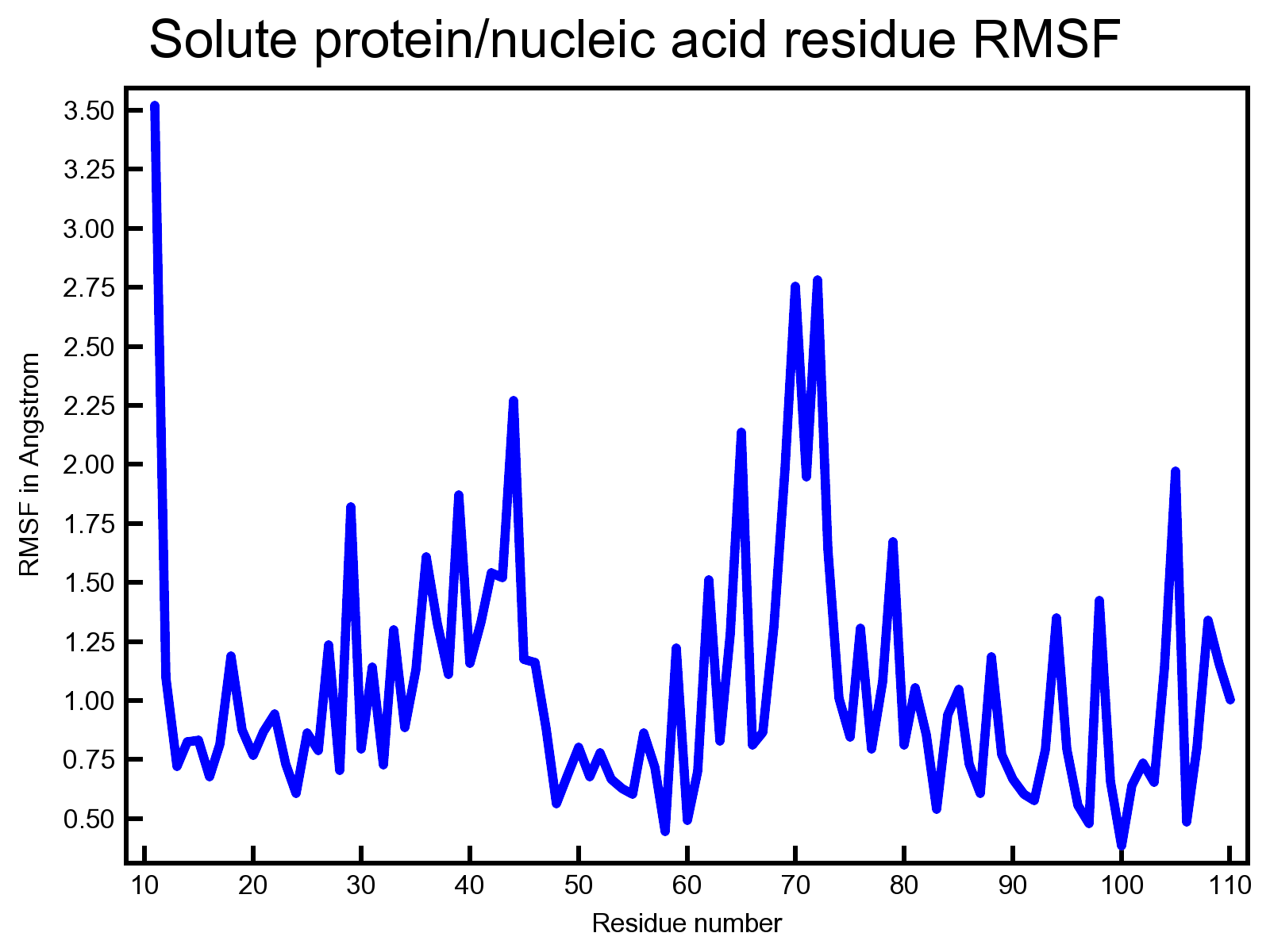

Supplement: S8 File — (ZIP) [file pone.0323003.s008.zip › Result/complex_1/complex_1_report_figure14_hires.png]

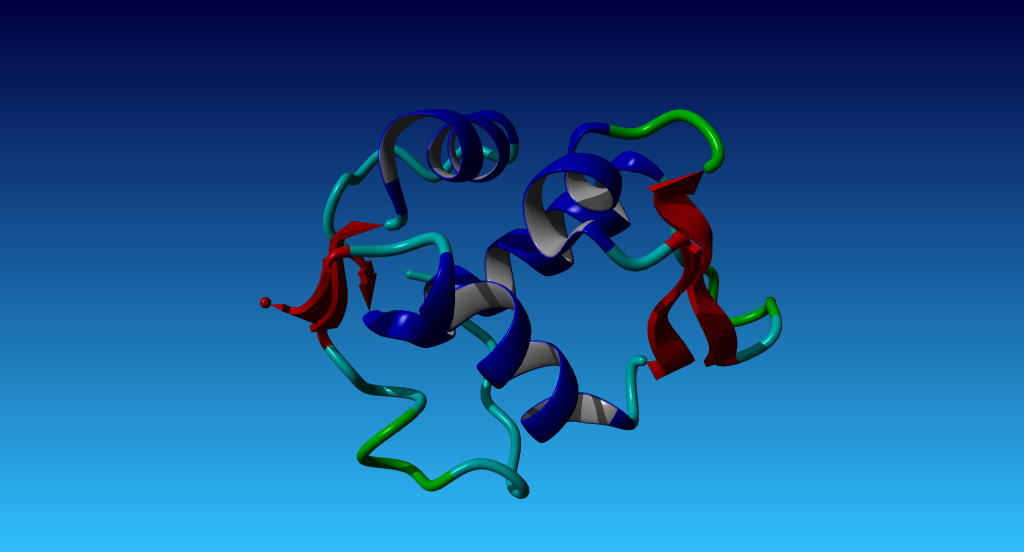

Supplement: S8 File — (ZIP) [file pone.0323003.s008.zip › Result/complex_1/complex_1_report_figure15.png]

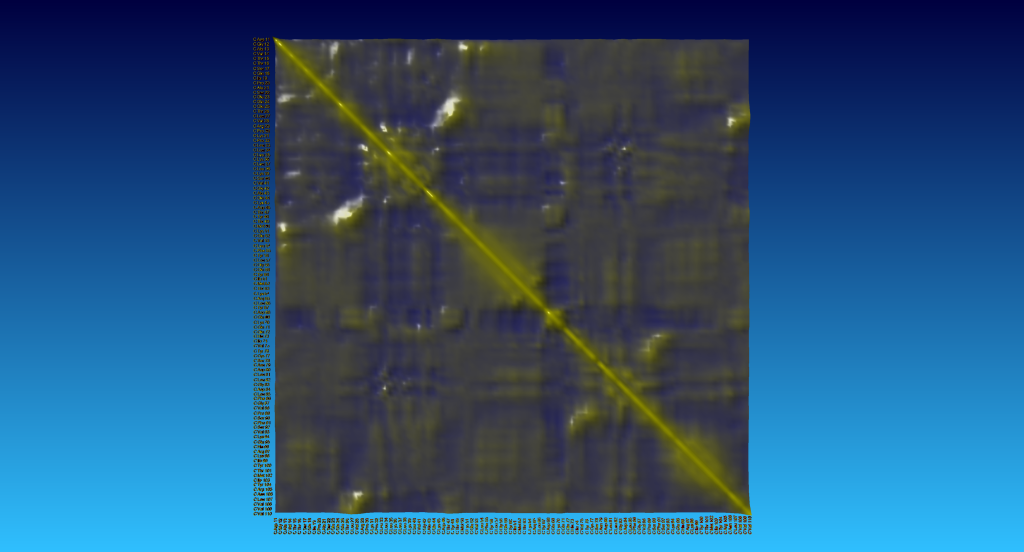

Supplement: S8 File — (ZIP) [file pone.0323003.s008.zip › Result/complex_1/complex_1_report_figure16.png]

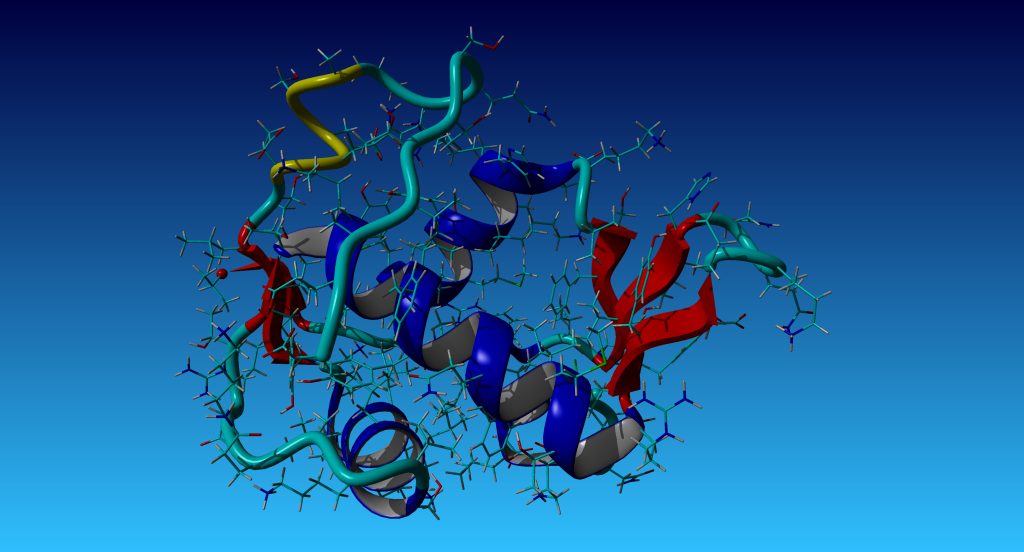

Supplement: S8 File — (ZIP) [file pone.0323003.s008.zip › Result/complex_1/complex_1_report_figure2.png]

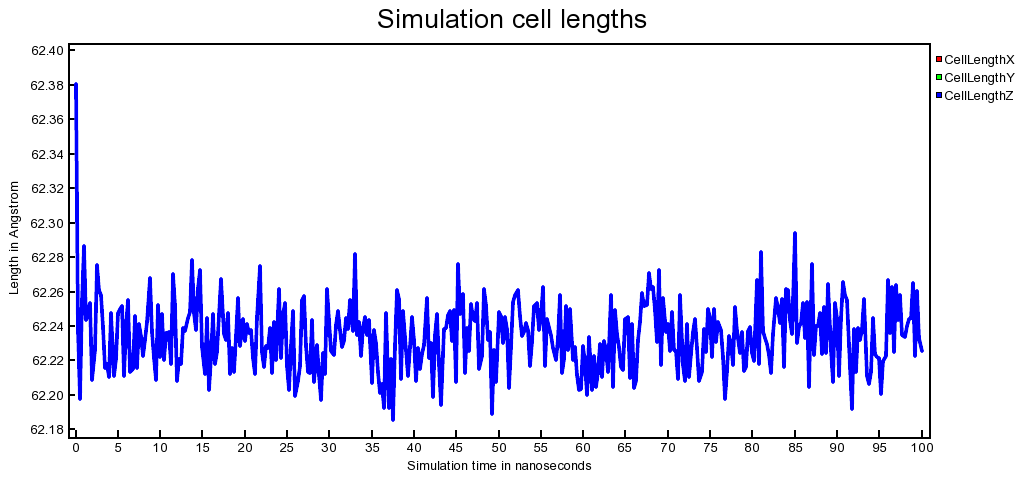

Supplement: S8 File — (ZIP) [file pone.0323003.s008.zip › Result/complex_1/complex_1_report_figure3.png]

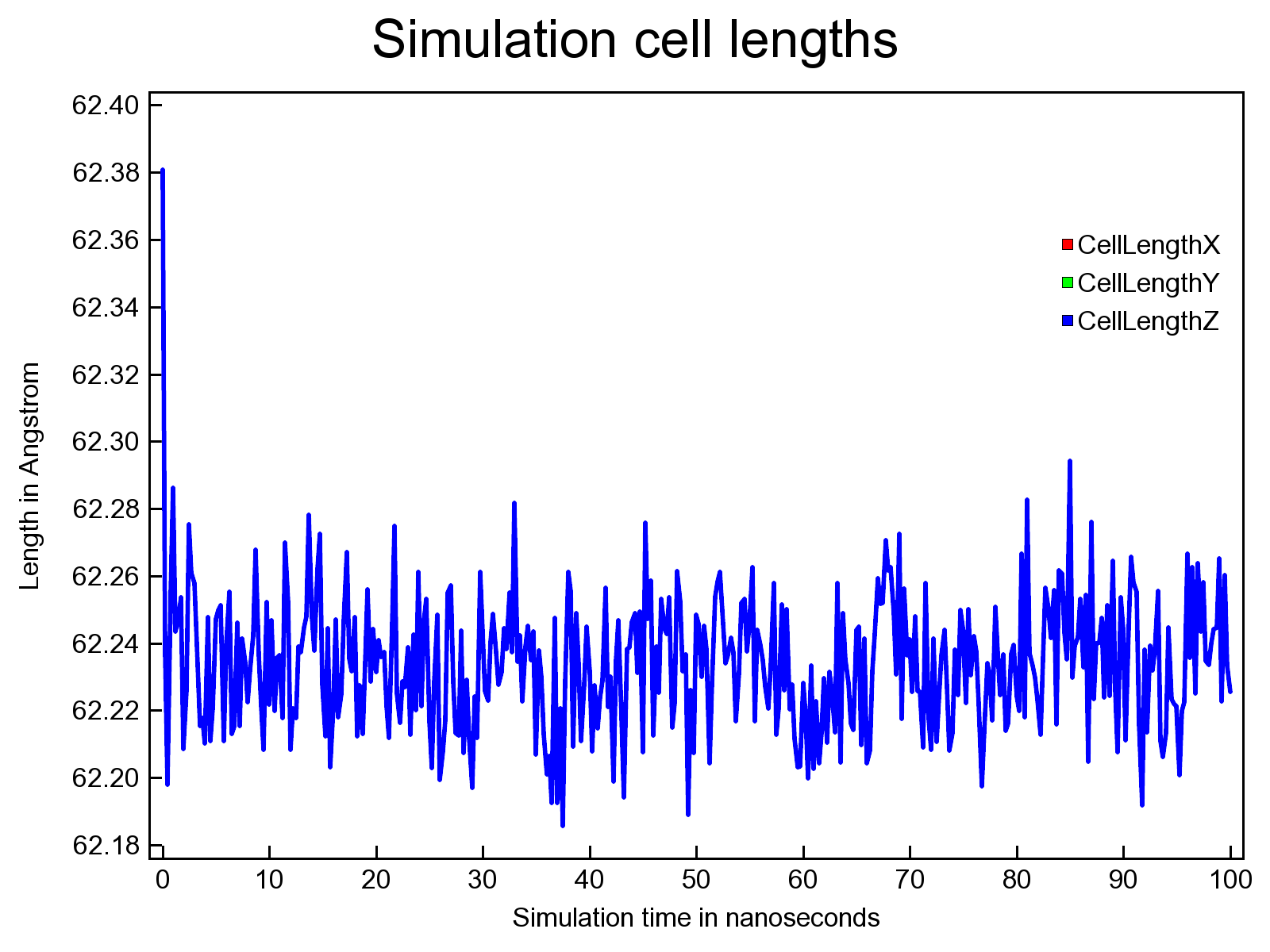

Supplement: S8 File — (ZIP) [file pone.0323003.s008.zip › Result/complex_1/complex_1_report_figure3_hires.png]

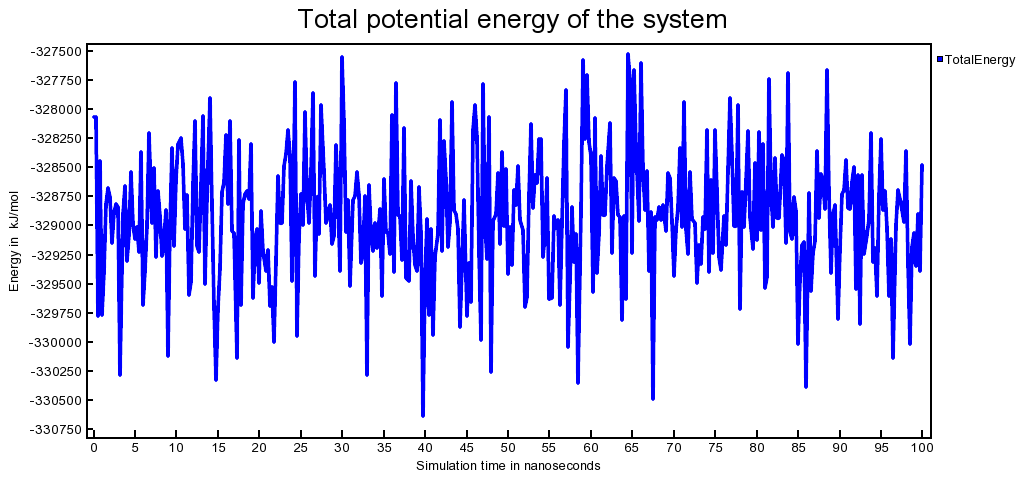

Supplement: S8 File — (ZIP) [file pone.0323003.s008.zip › Result/complex_1/complex_1_report_figure4.png]

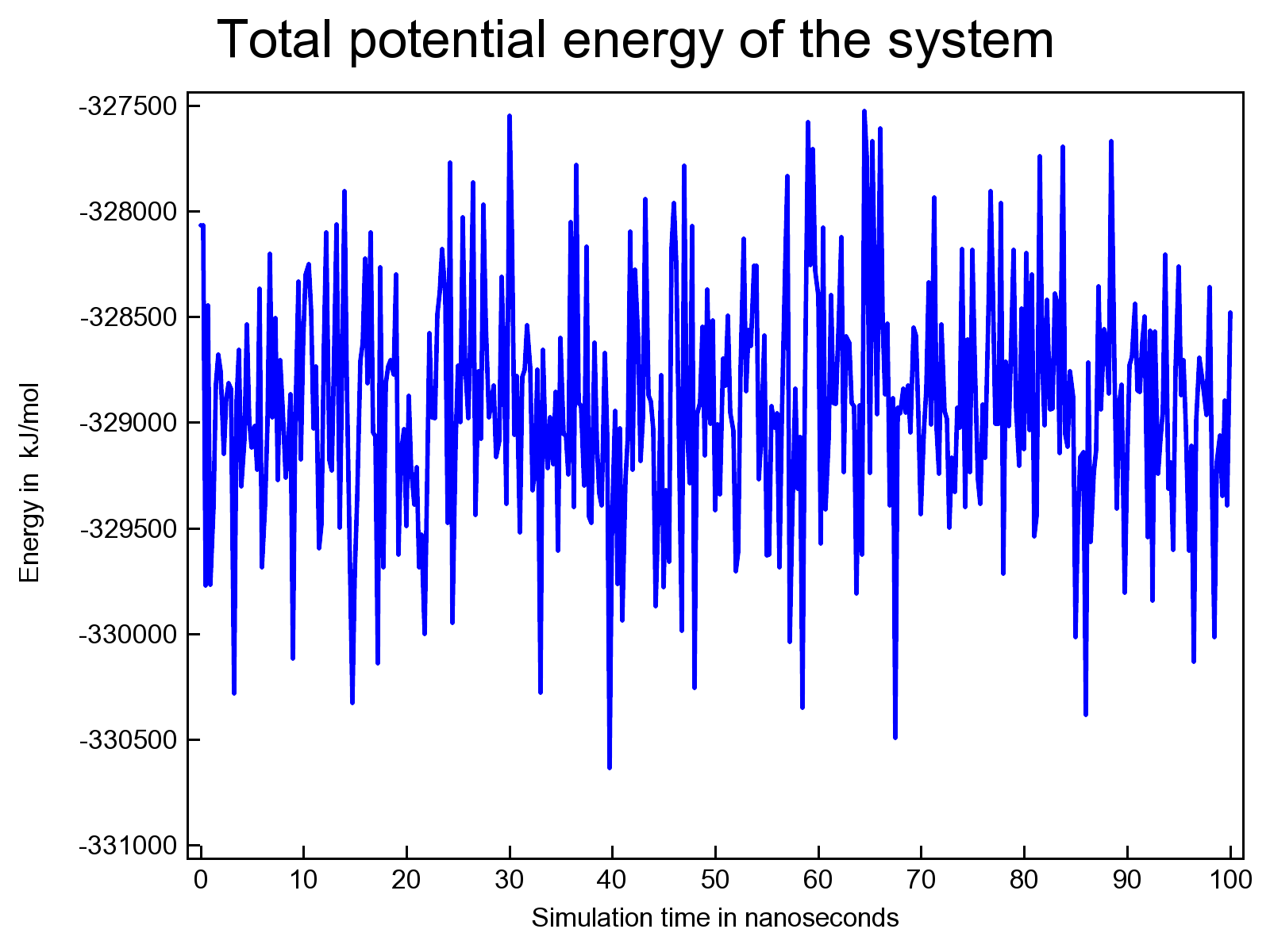

Supplement: S8 File — (ZIP) [file pone.0323003.s008.zip › Result/complex_1/complex_1_report_figure4_hires.png]

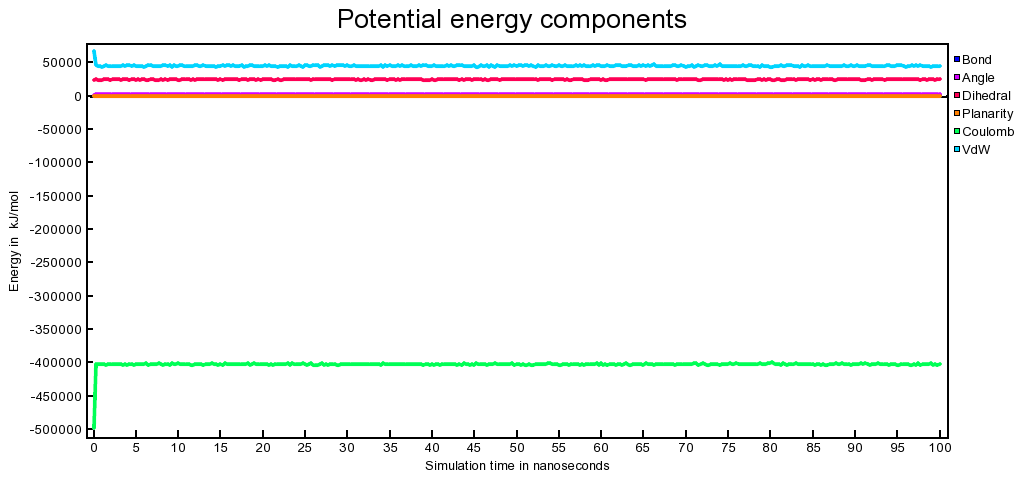

Supplement: S8 File — (ZIP) [file pone.0323003.s008.zip › Result/complex_1/complex_1_report_figure5.png]

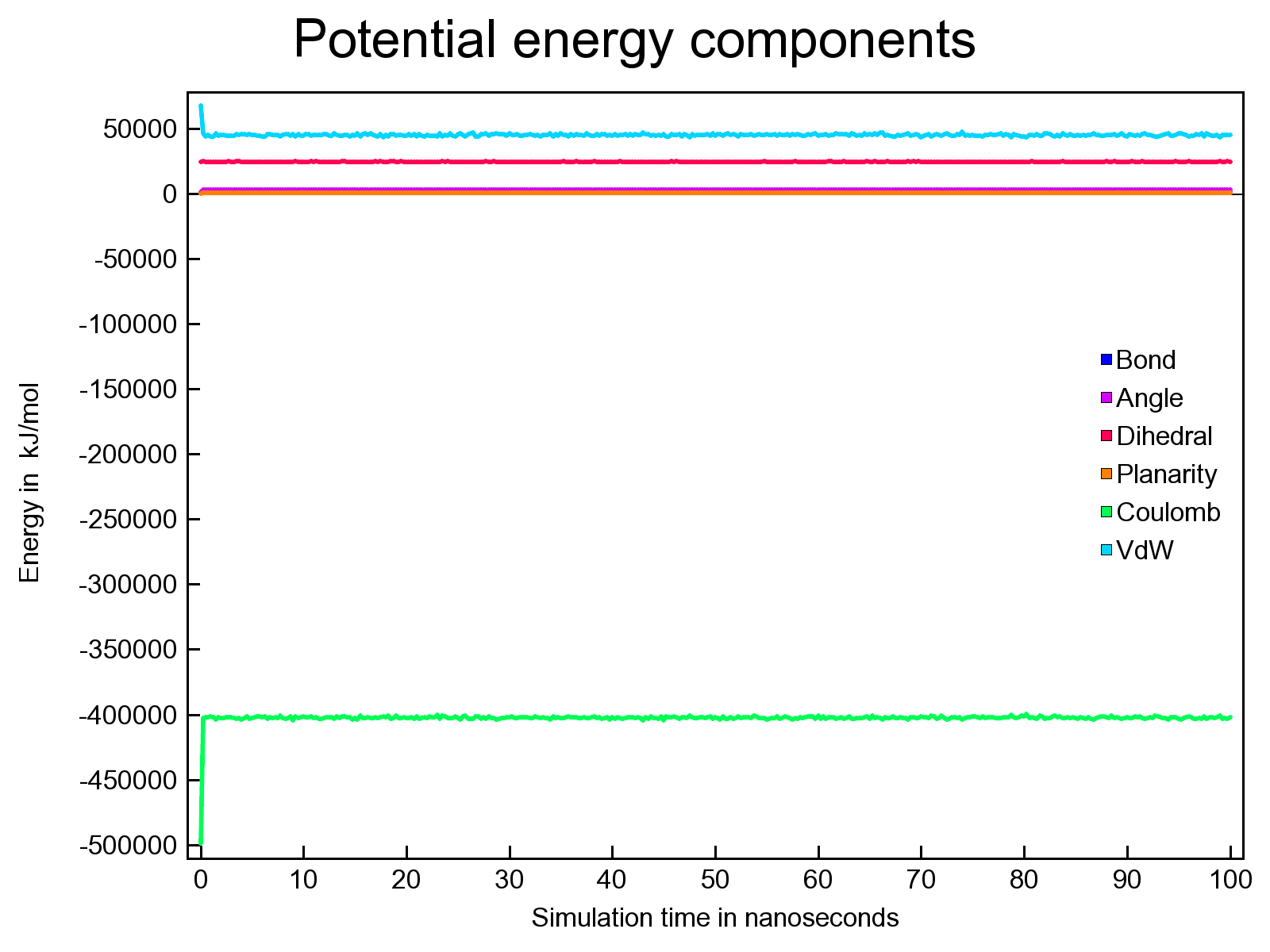

Supplement: S8 File — (ZIP) [file pone.0323003.s008.zip › Result/complex_1/complex_1_report_figure5_hires.png]

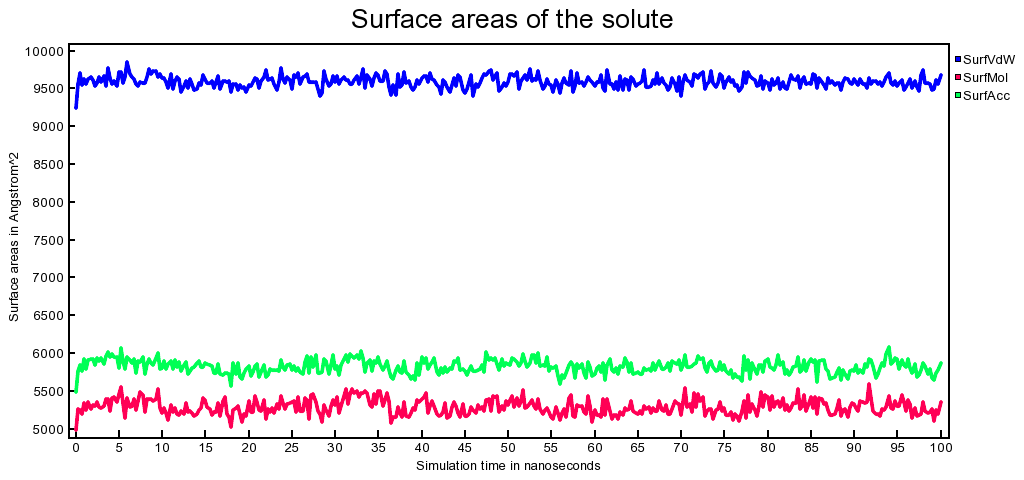

Supplement: S8 File — (ZIP) [file pone.0323003.s008.zip › Result/complex_1/complex_1_report_figure6.png]

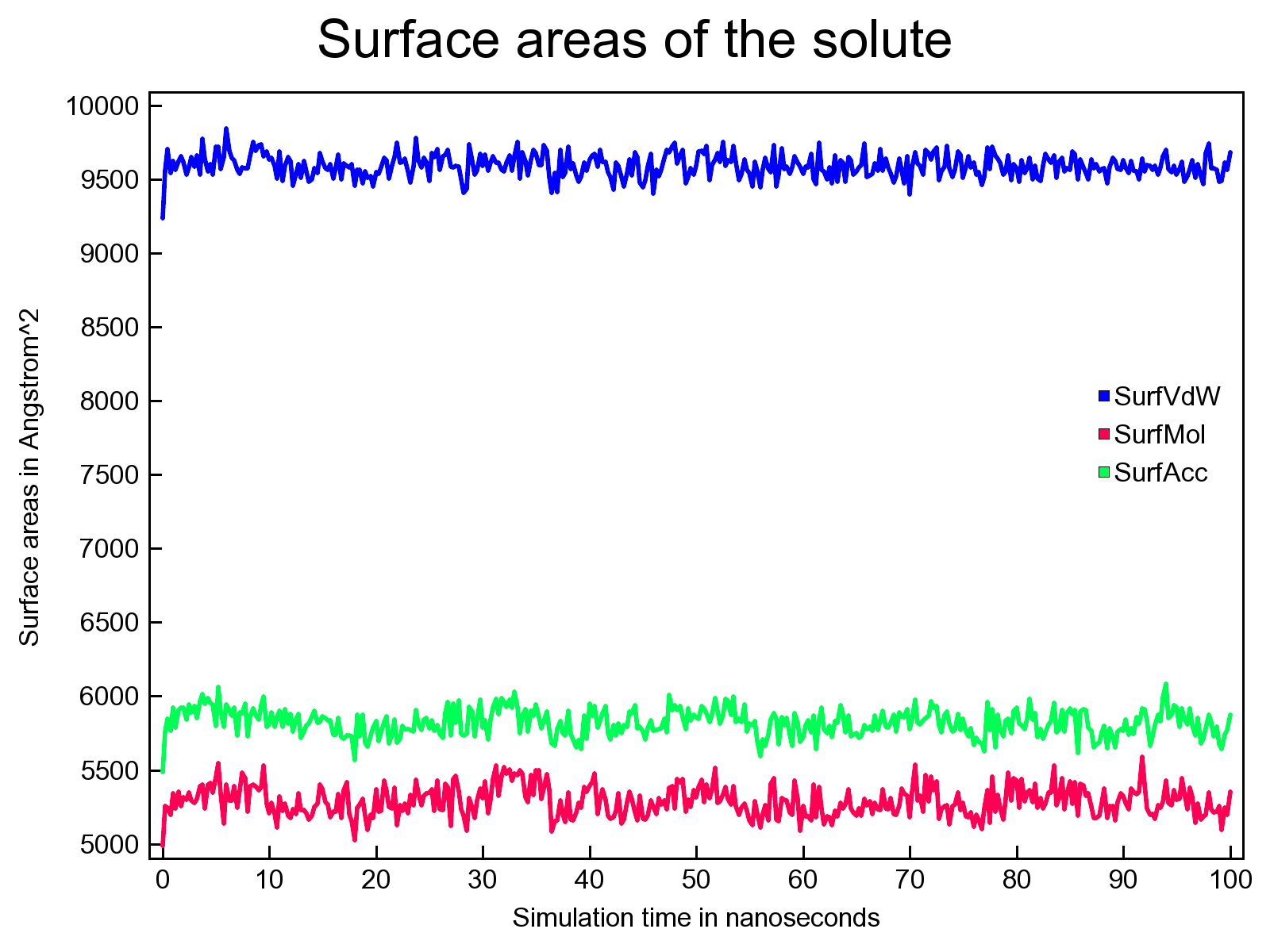

Supplement: S8 File — (ZIP) [file pone.0323003.s008.zip › Result/complex_1/complex_1_report_figure6_hires.png]

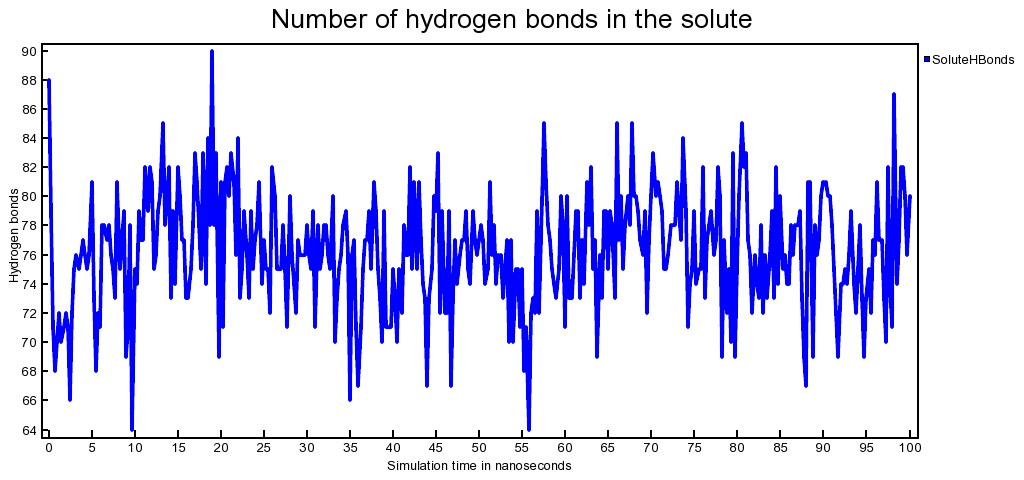

Supplement: S8 File — (ZIP) [file pone.0323003.s008.zip › Result/complex_1/complex_1_report_figure7.png]

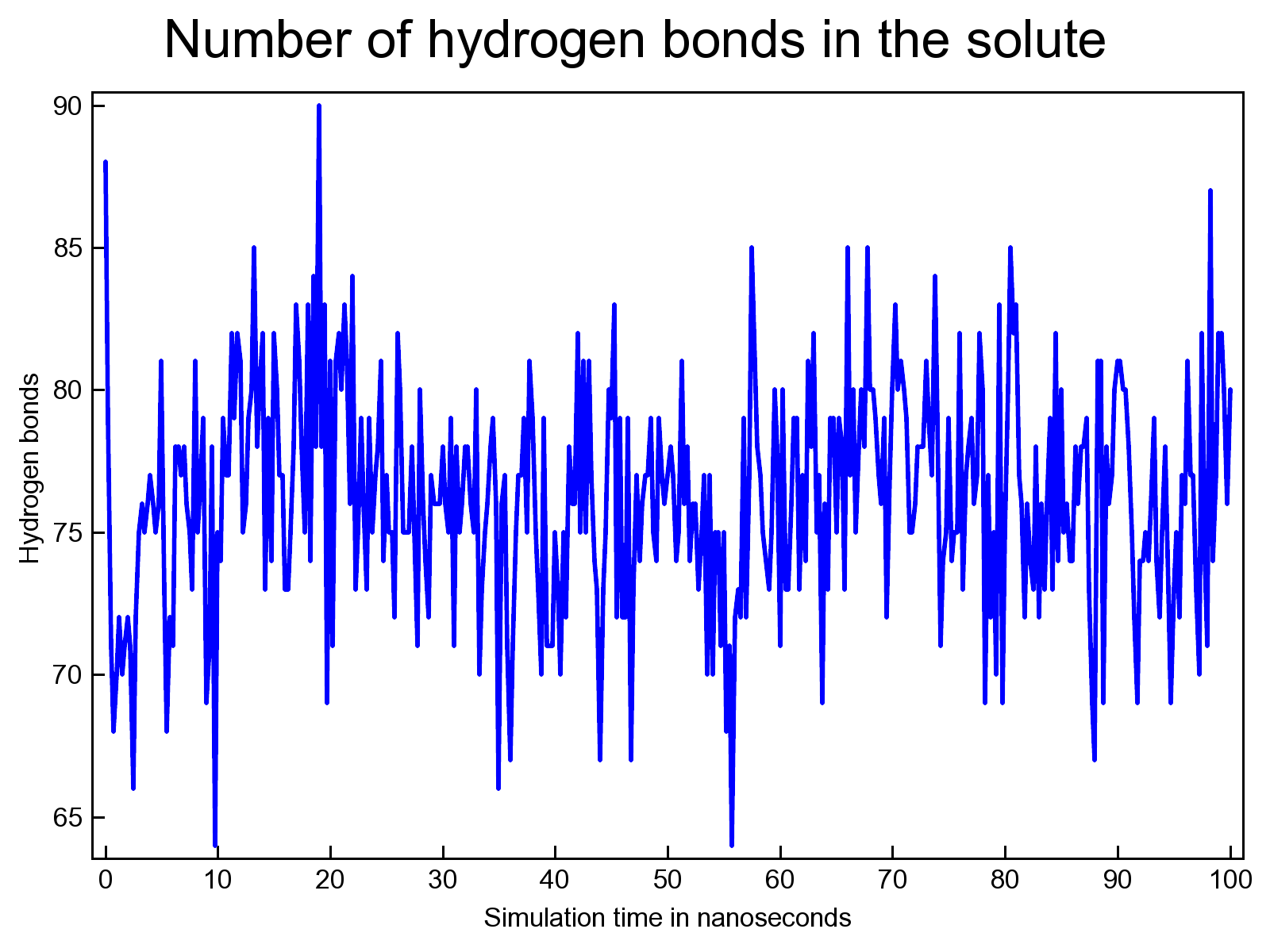

Supplement: S8 File — (ZIP) [file pone.0323003.s008.zip › Result/complex_1/complex_1_report_figure7_hires.png]

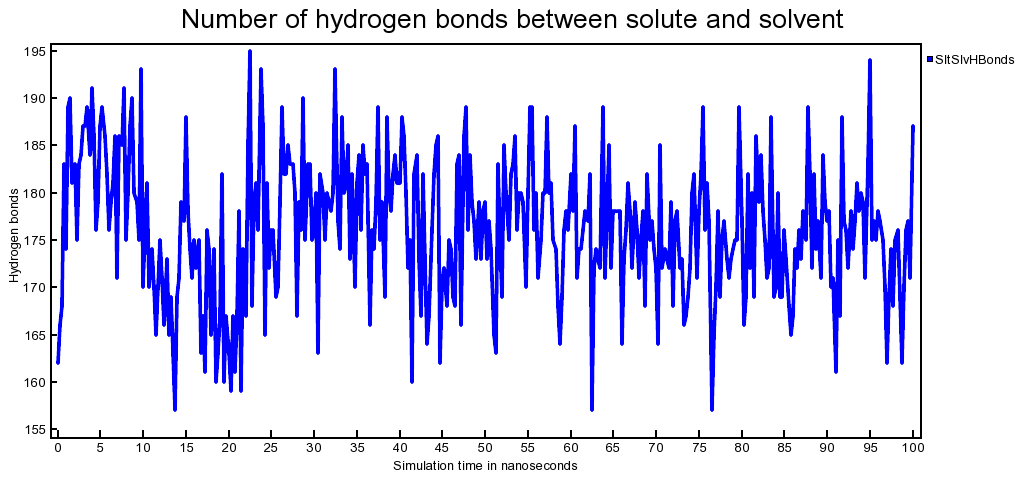

Supplement: S8 File — (ZIP) [file pone.0323003.s008.zip › Result/complex_1/complex_1_report_figure8.png]

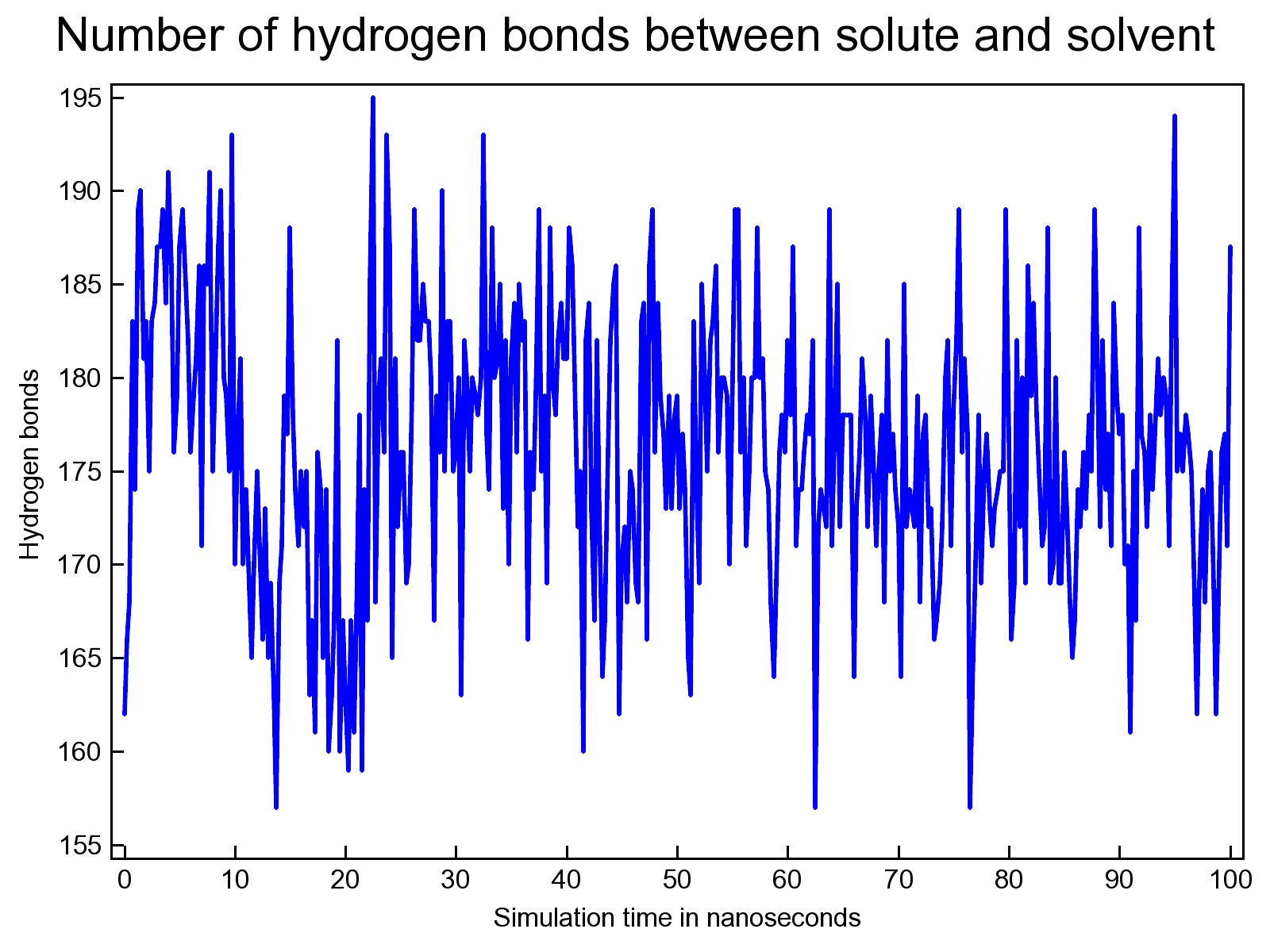

Supplement: S8 File — (ZIP) [file pone.0323003.s008.zip › Result/complex_1/complex_1_report_figure8_hires.png]

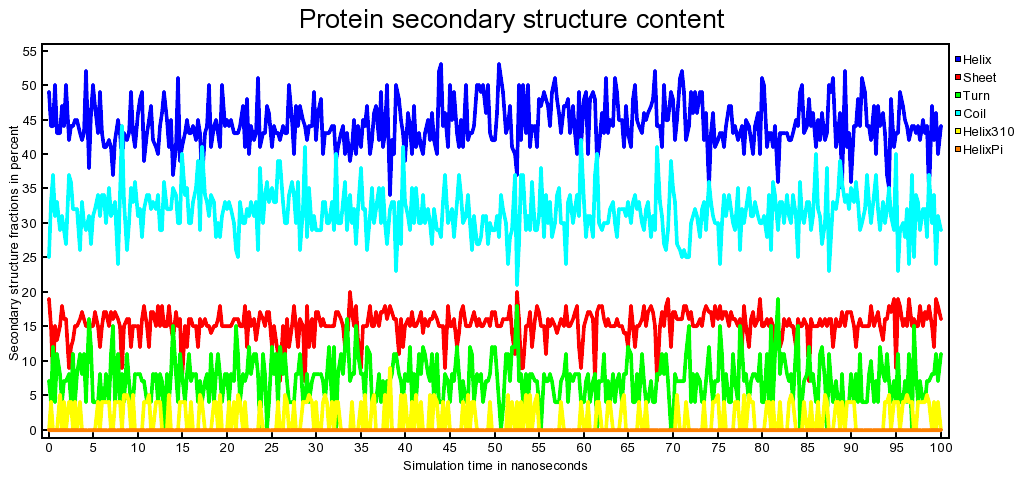

Supplement: S8 File — (ZIP) [file pone.0323003.s008.zip › Result/complex_1/complex_1_report_figure9.png]

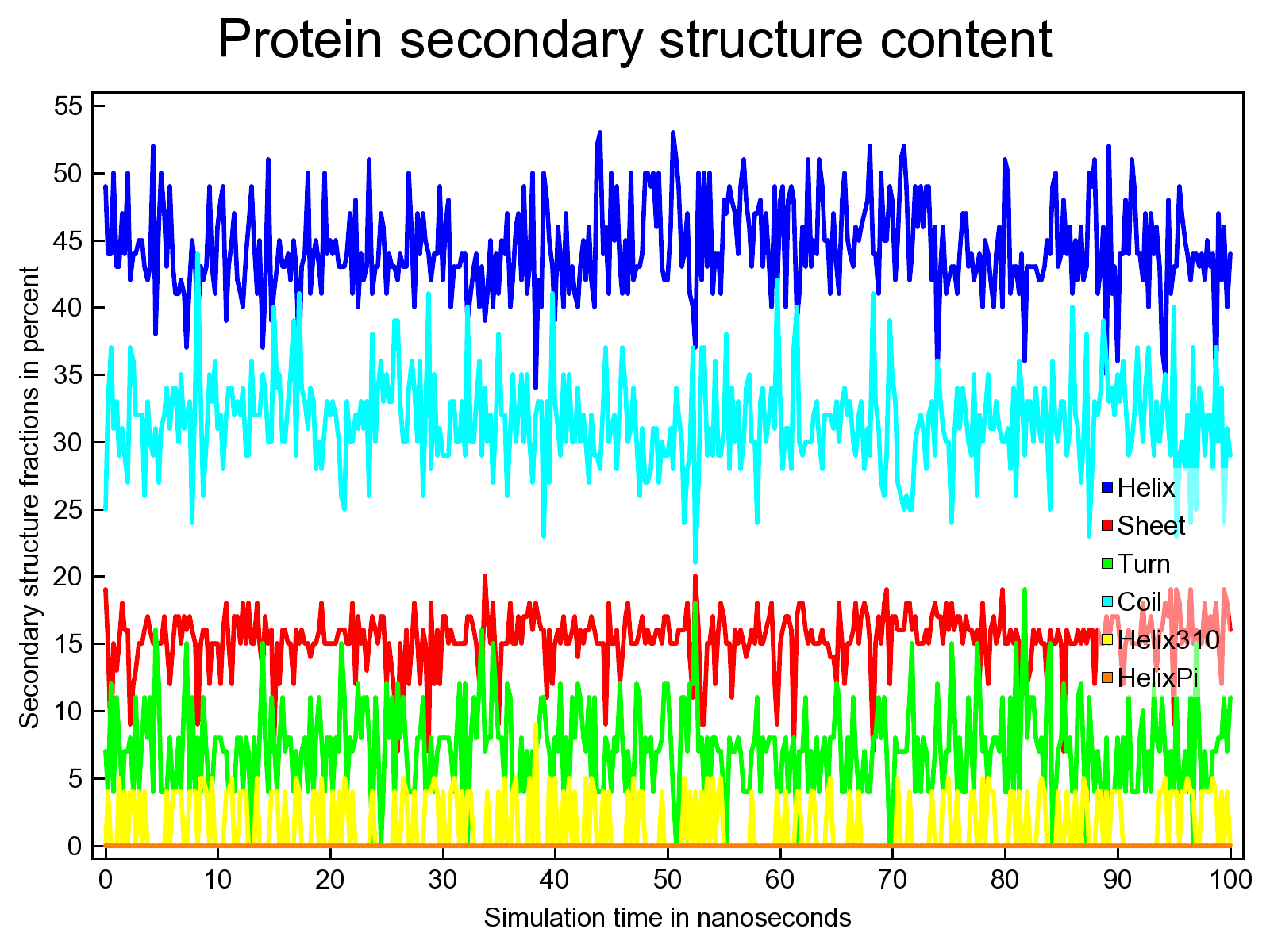

Supplement: S8 File — (ZIP) [file pone.0323003.s008.zip › Result/complex_1/complex_1_report_figure9_hires.png]

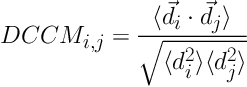

Supplement: S8 File — (ZIP) [file pone.0323003.s008.zip › Result/complex_1/complex_1_report_formula_dccm.png]

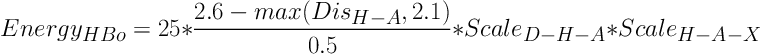

Supplement: S8 File — (ZIP) [file pone.0323003.s008.zip › Result/complex_1/complex_1_report_formula_energyhbo0.png]

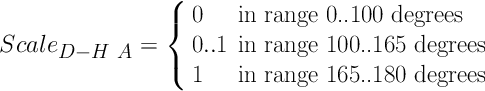

Supplement: S8 File — (ZIP) [file pone.0323003.s008.zip › Result/complex_1/complex_1_report_formula_energyhbo1.png]

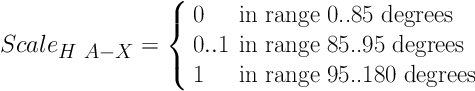

Supplement: S8 File — (ZIP) [file pone.0323003.s008.zip › Result/complex_1/complex_1_report_formula_energyhbo2.png]

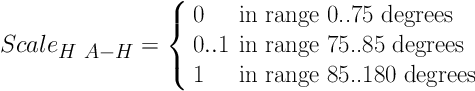

Supplement: S8 File — (ZIP) [file pone.0323003.s008.zip › Result/complex_1/complex_1_report_formula_energyhbo3.png]

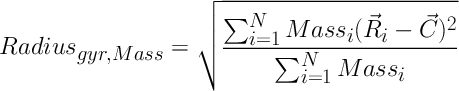

Supplement: S8 File — (ZIP) [file pone.0323003.s008.zip › Result/complex_1/complex_1_report_formula_gyrrad.png]

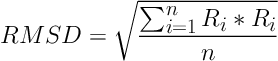

Supplement: S8 File — (ZIP) [file pone.0323003.s008.zip › Result/complex_1/complex_1_report_formula_rmsd.png]

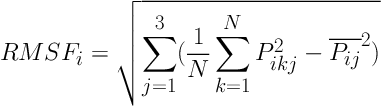

Supplement: S8 File — (ZIP) [file pone.0323003.s008.zip › Result/complex_1/complex_1_report_formula_rmsf.png]

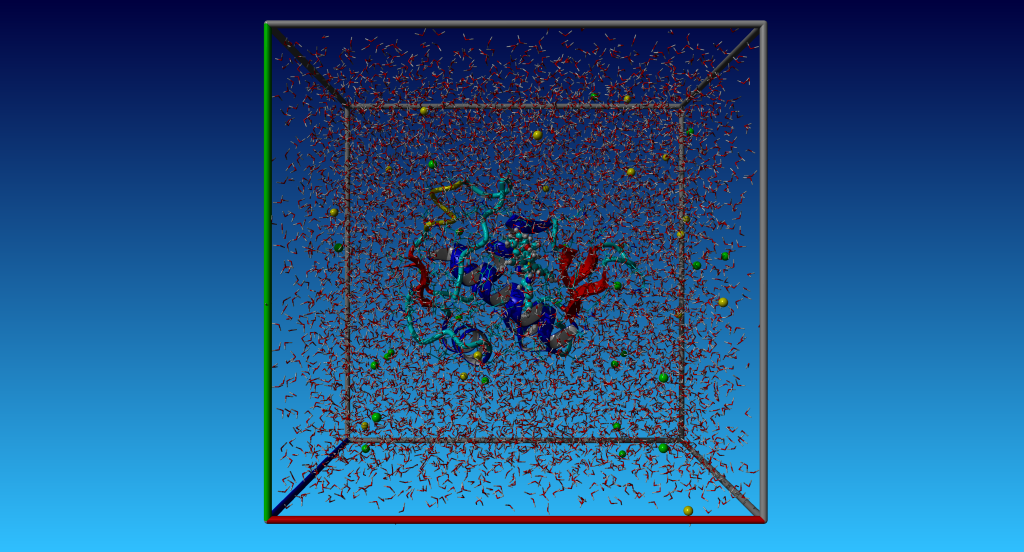

Supplement: S8 File — (ZIP) [file pone.0323003.s008.zip › Result/complex_2/complex_2_report_figure1.png]

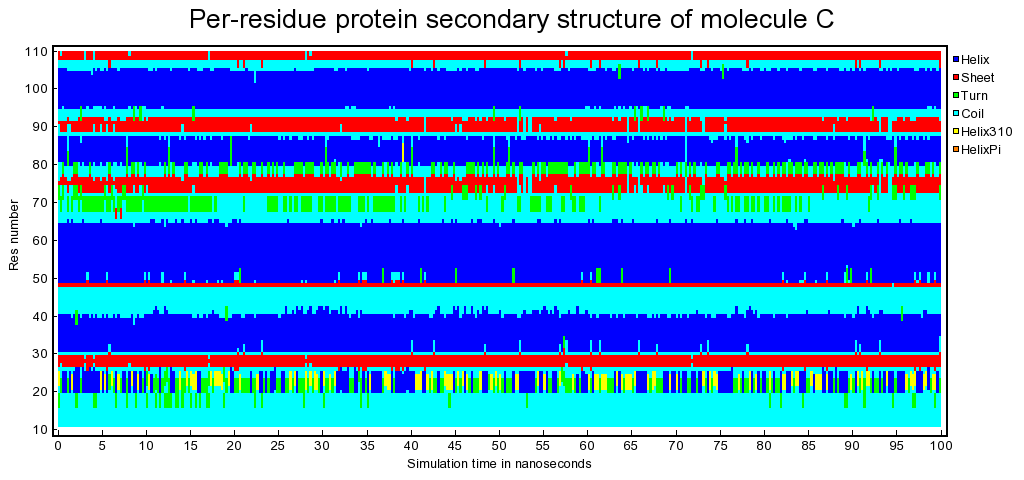

Supplement: S8 File — (ZIP) [file pone.0323003.s008.zip › Result/complex_2/complex_2_report_figure10.png]

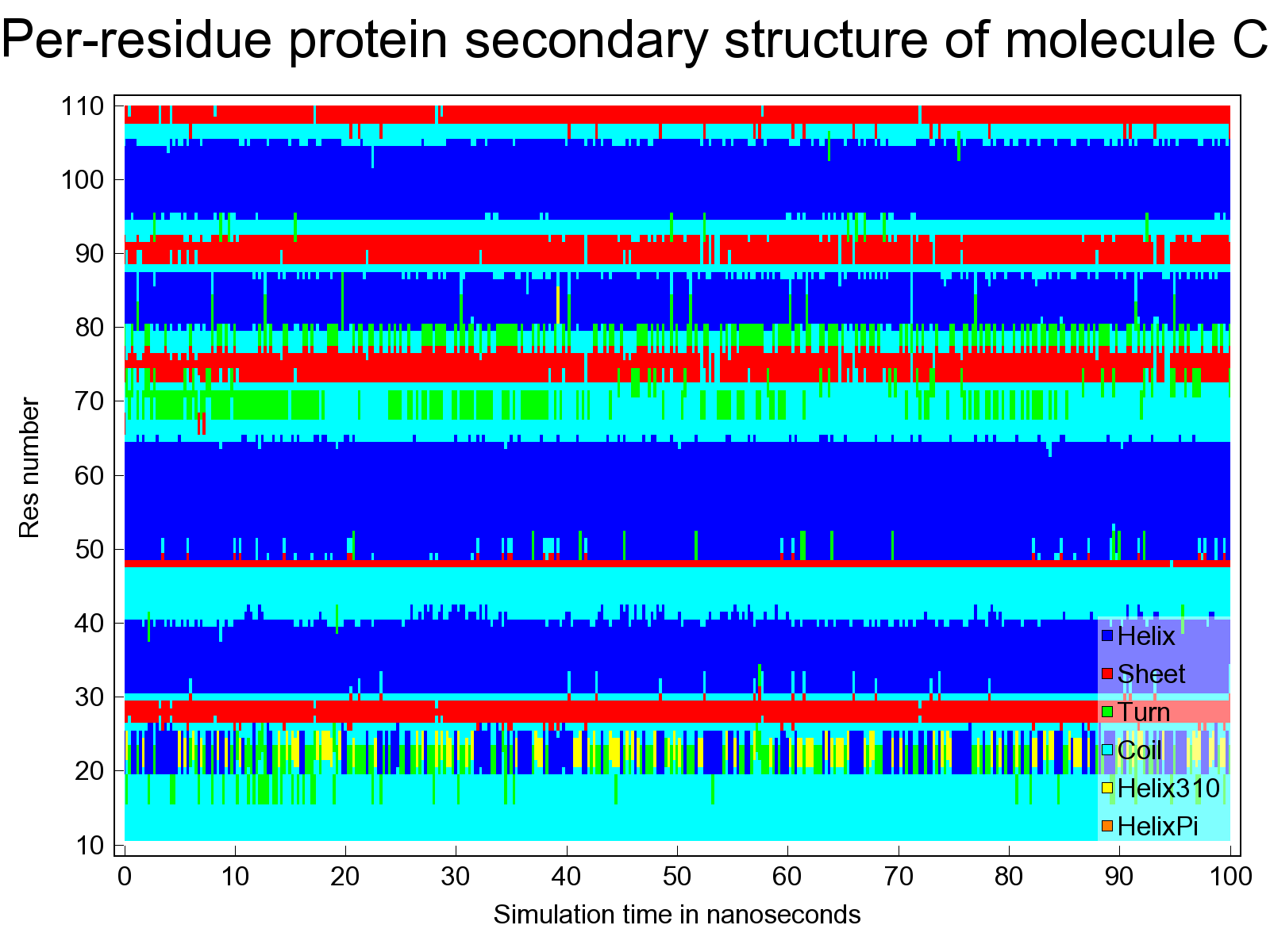

Supplement: S8 File — (ZIP) [file pone.0323003.s008.zip › Result/complex_2/complex_2_report_figure10_hires.png]

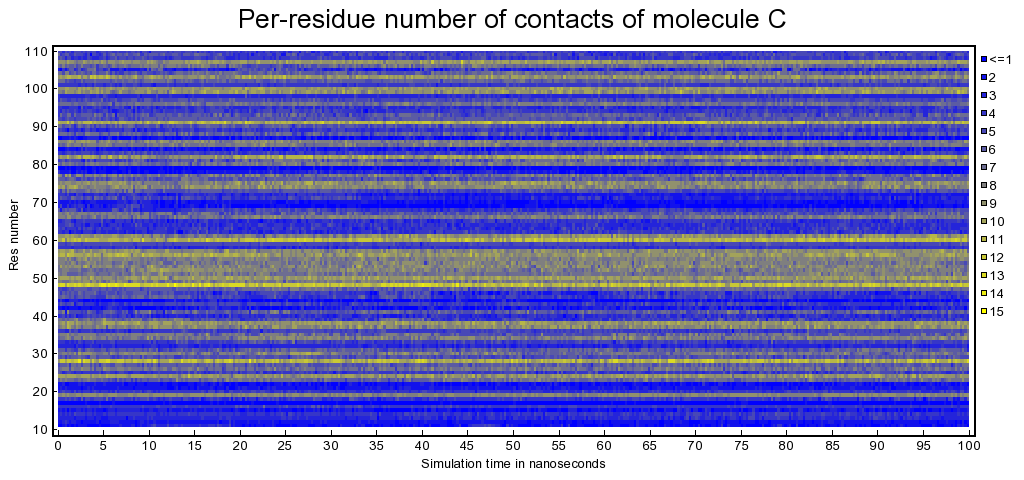

Supplement: S8 File — (ZIP) [file pone.0323003.s008.zip › Result/complex_2/complex_2_report_figure11.png]

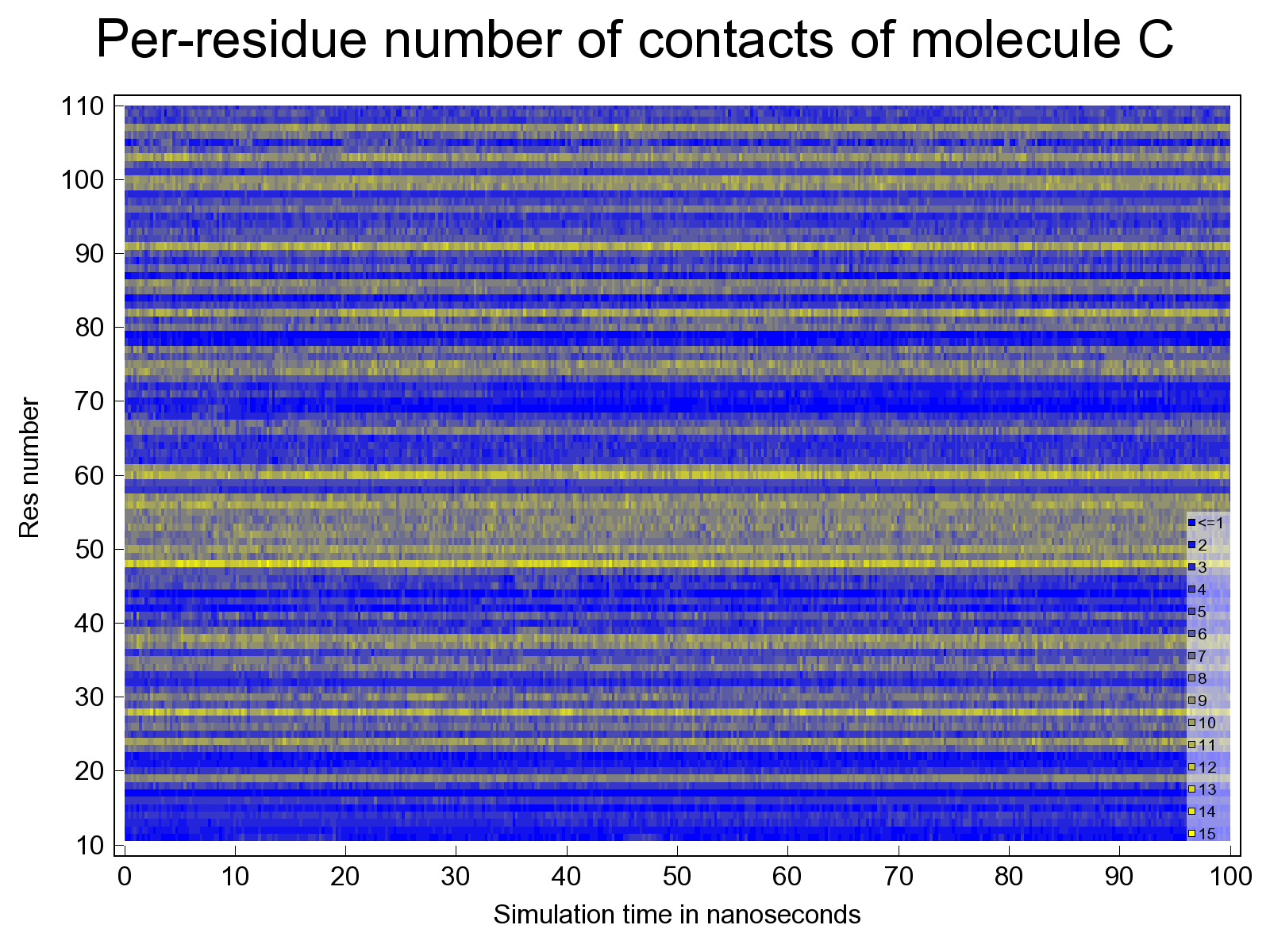

Supplement: S8 File — (ZIP) [file pone.0323003.s008.zip › Result/complex_2/complex_2_report_figure11_hires.png]

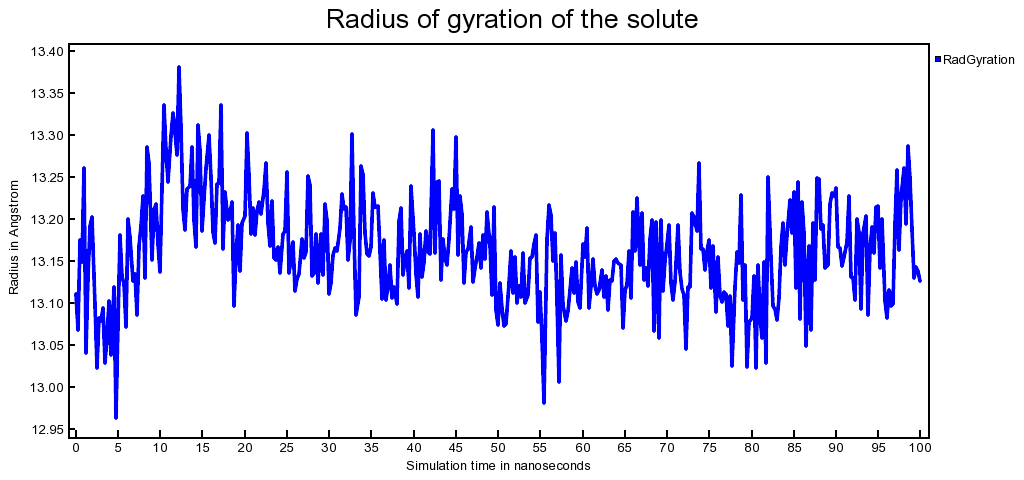

Supplement: S8 File — (ZIP) [file pone.0323003.s008.zip › Result/complex_2/complex_2_report_figure12.png]

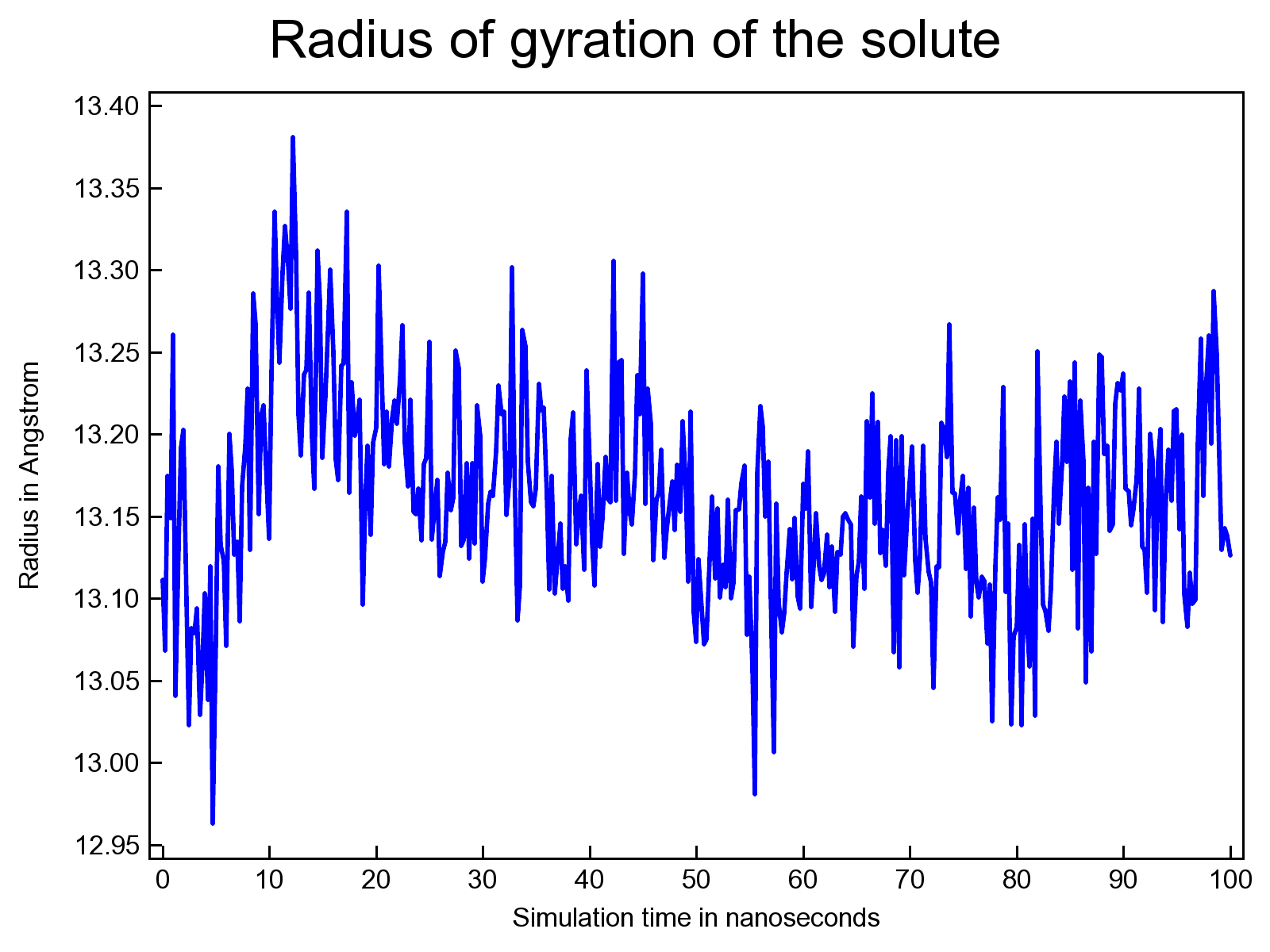

Supplement: S8 File — (ZIP) [file pone.0323003.s008.zip › Result/complex_2/complex_2_report_figure12_hires.png]

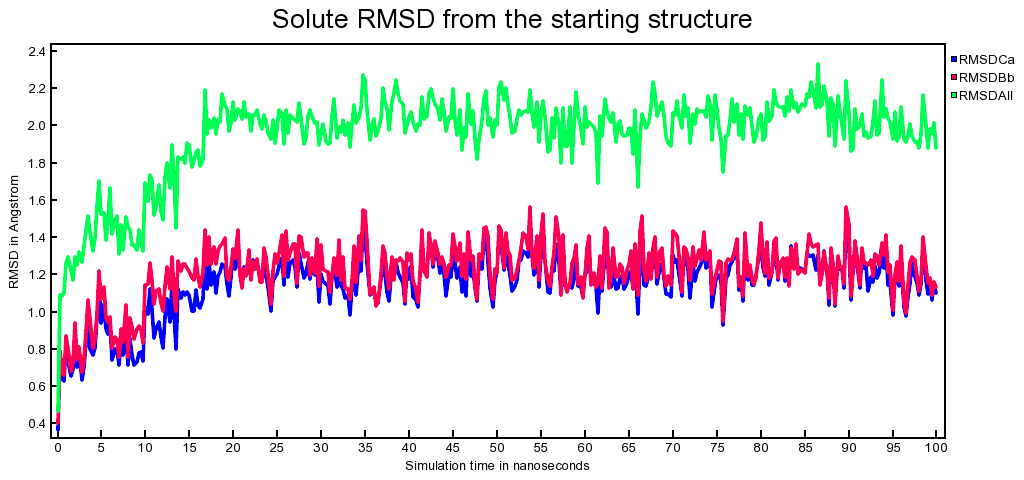

Supplement: S8 File — (ZIP) [file pone.0323003.s008.zip › Result/complex_2/complex_2_report_figure13.png]

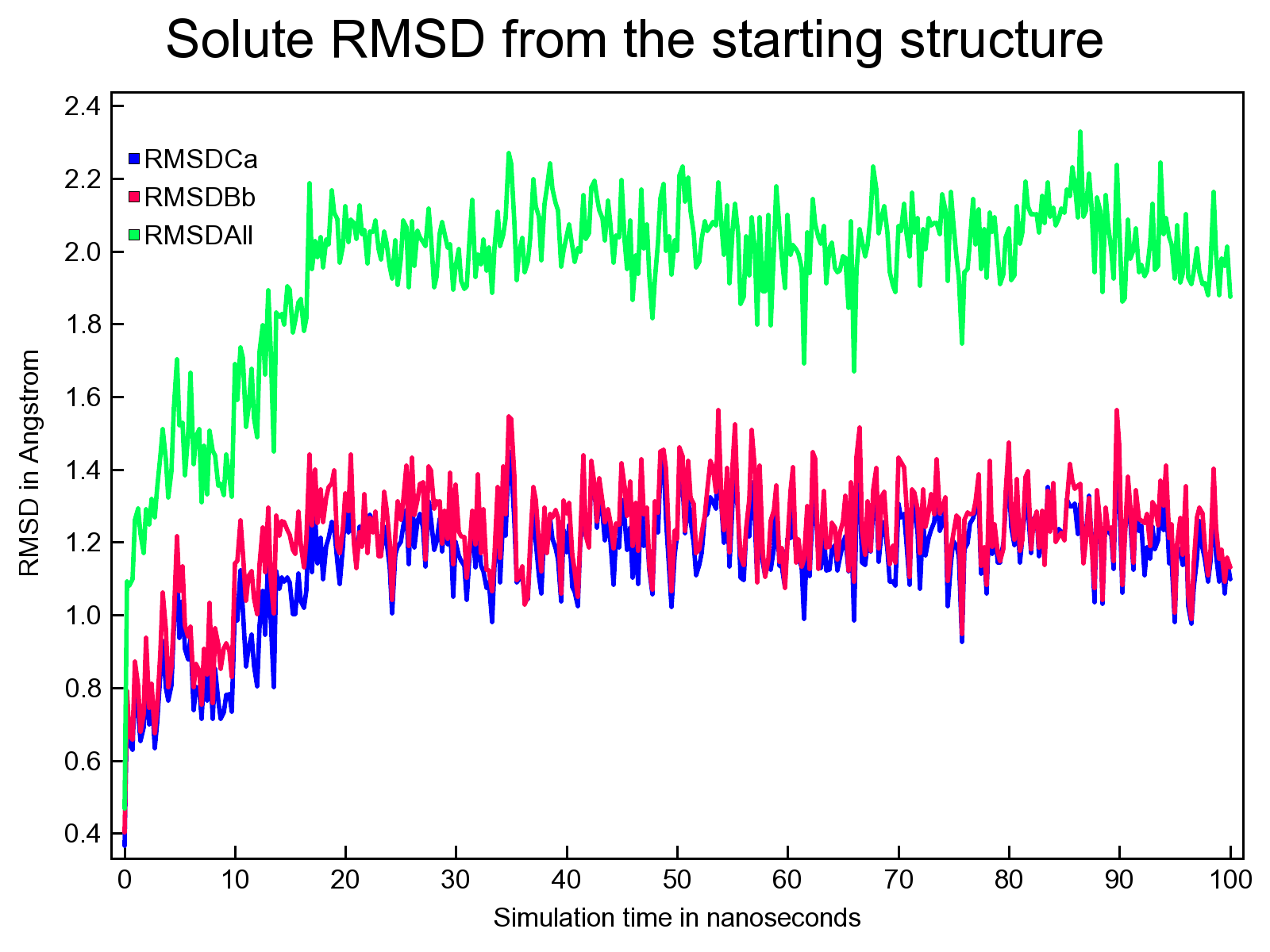

Supplement: S8 File — (ZIP) [file pone.0323003.s008.zip › Result/complex_2/complex_2_report_figure13_hires.png]

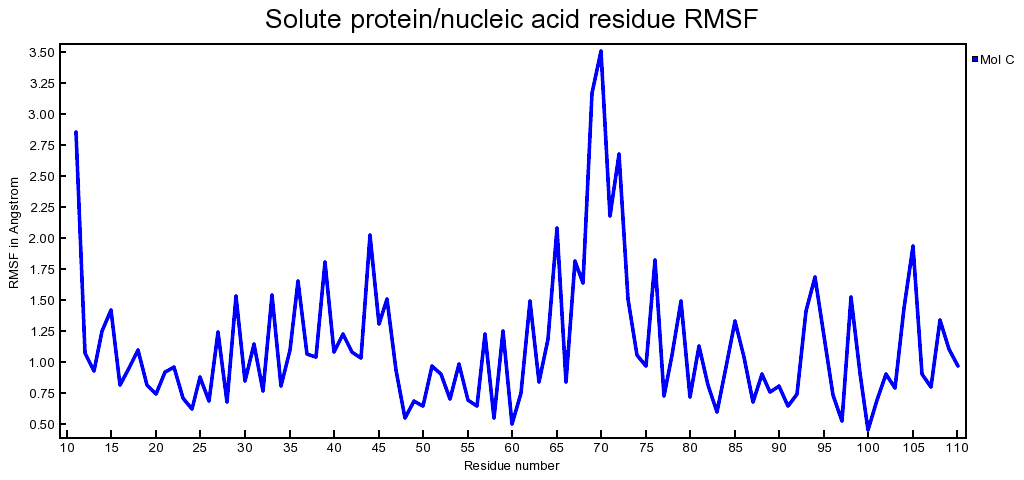

Supplement: S8 File — (ZIP) [file pone.0323003.s008.zip › Result/complex_2/complex_2_report_figure14.png]

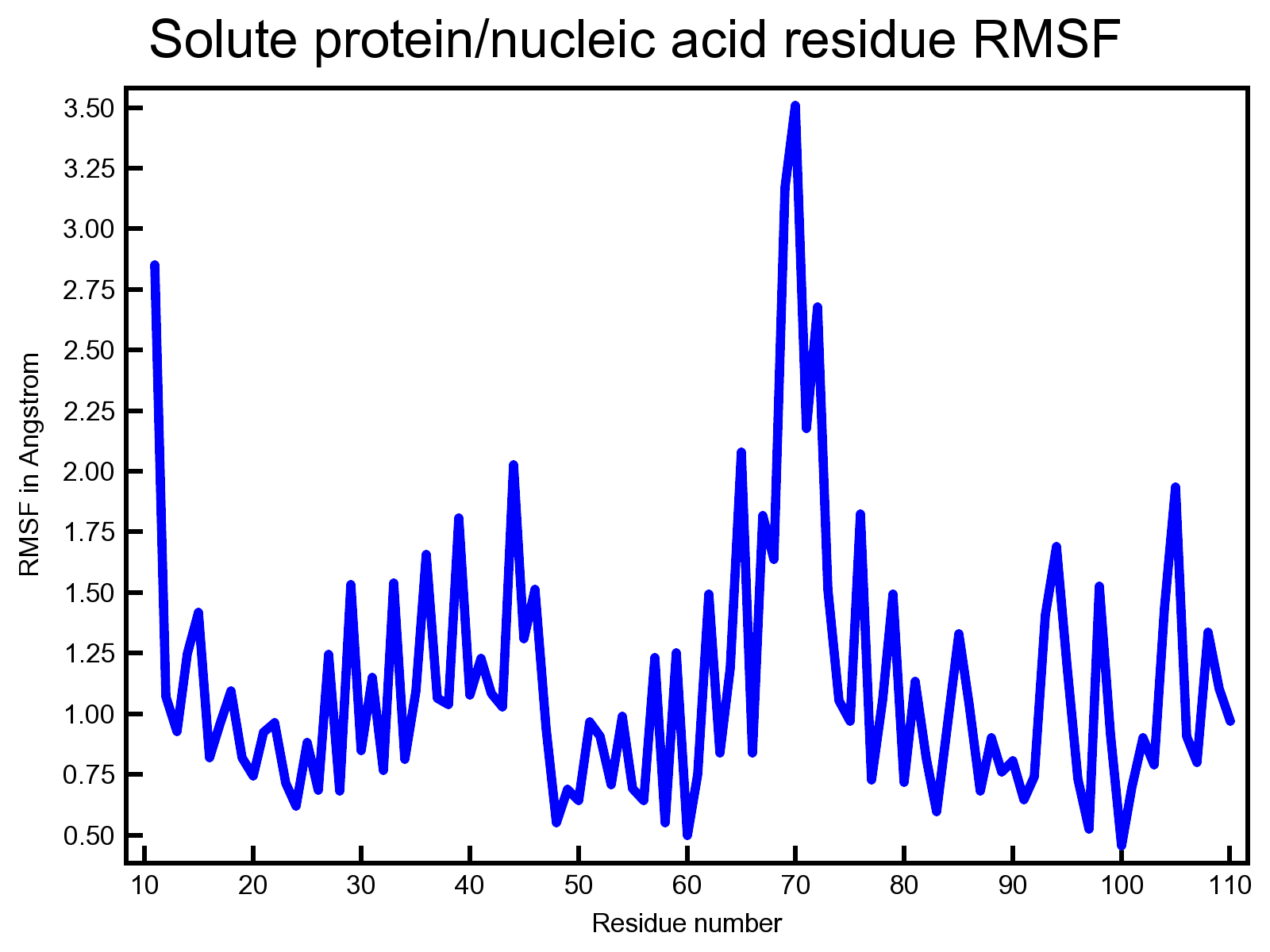

Supplement: S8 File — (ZIP) [file pone.0323003.s008.zip › Result/complex_2/complex_2_report_figure14_hires.png]

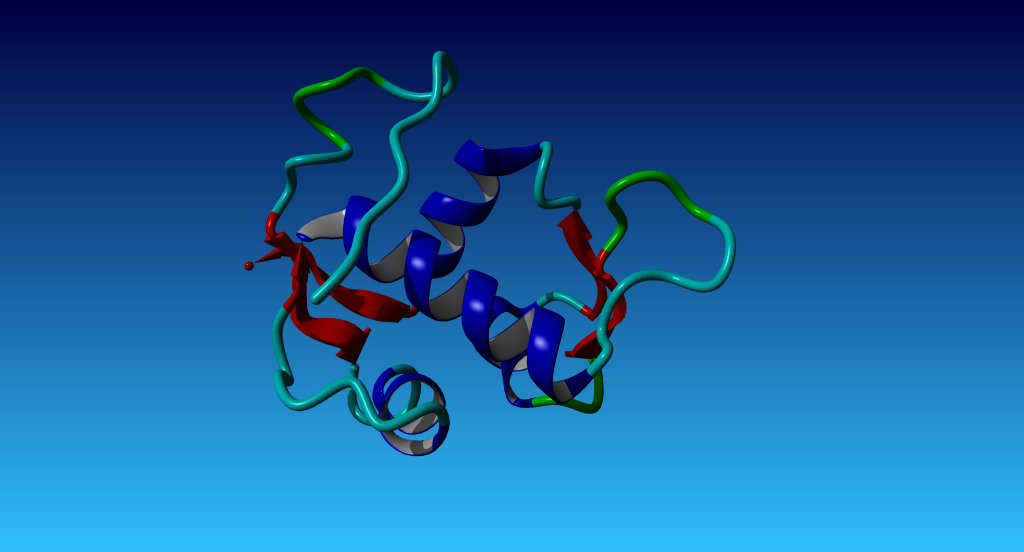

Supplement: S8 File — (ZIP) [file pone.0323003.s008.zip › Result/complex_2/complex_2_report_figure15.png]

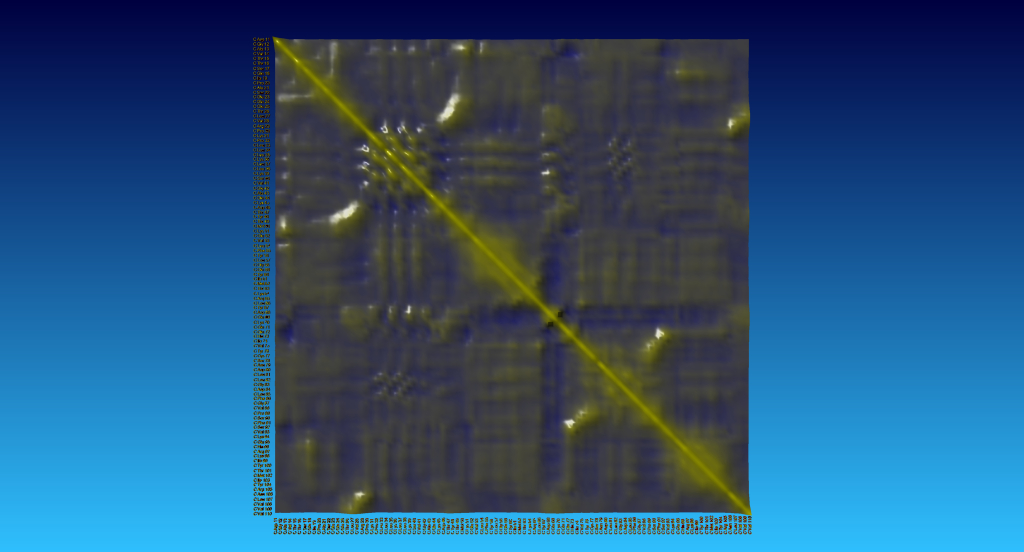

Supplement: S8 File — (ZIP) [file pone.0323003.s008.zip › Result/complex_2/complex_2_report_figure16.png]

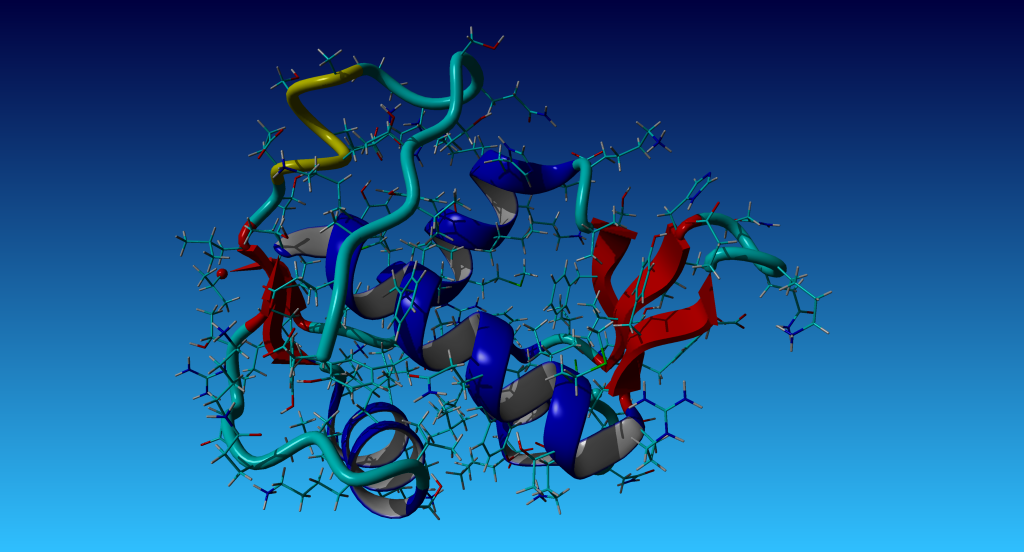

Supplement: S8 File — (ZIP) [file pone.0323003.s008.zip › Result/complex_2/complex_2_report_figure2.png]

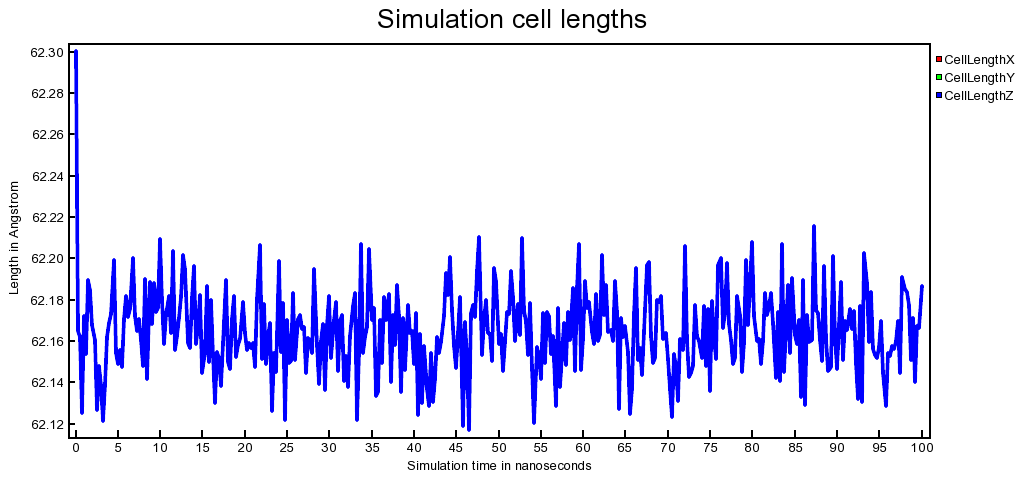

Supplement: S8 File — (ZIP) [file pone.0323003.s008.zip › Result/complex_2/complex_2_report_figure3.png]

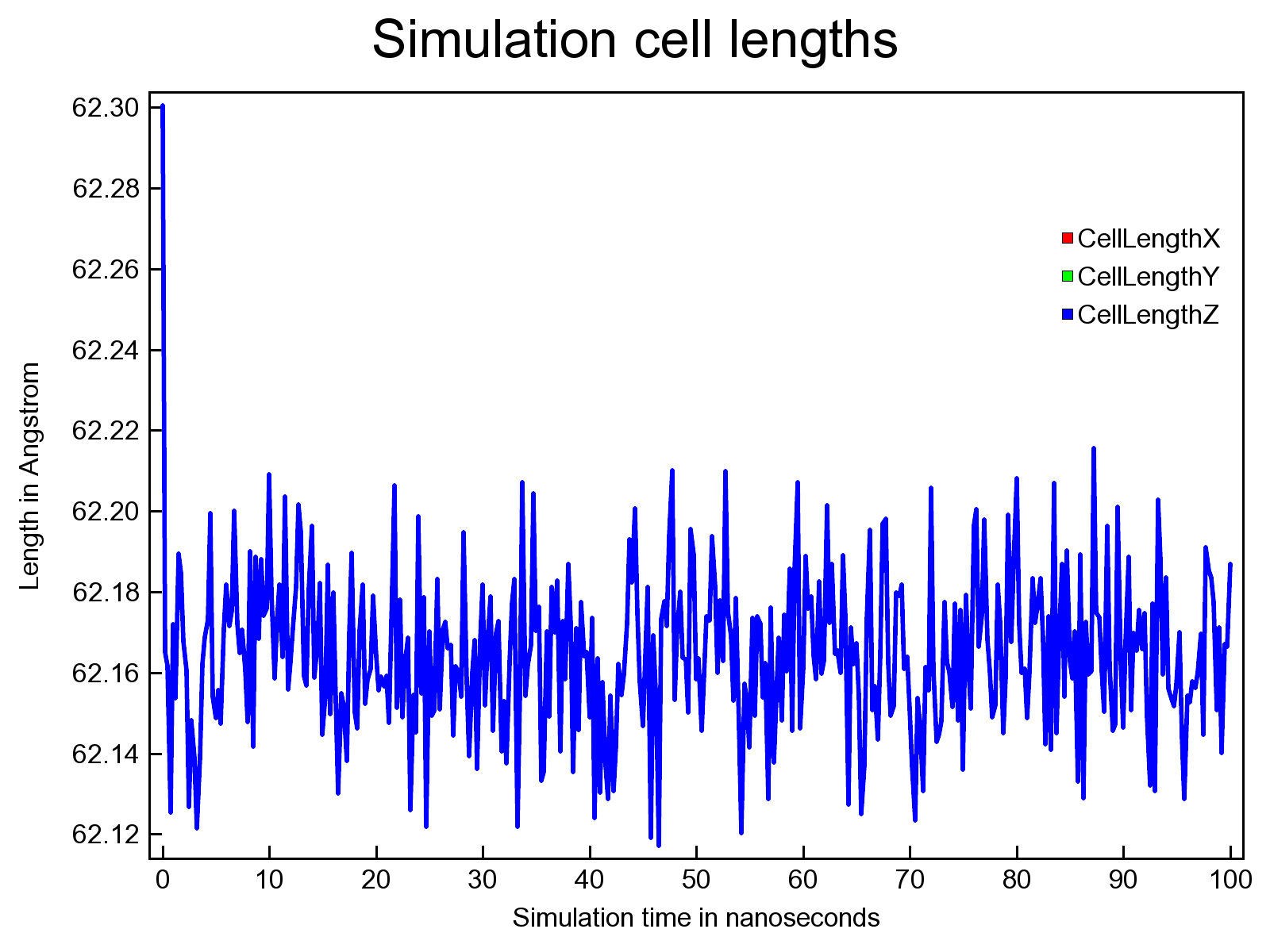

Supplement: S8 File — (ZIP) [file pone.0323003.s008.zip › Result/complex_2/complex_2_report_figure3_hires.png]

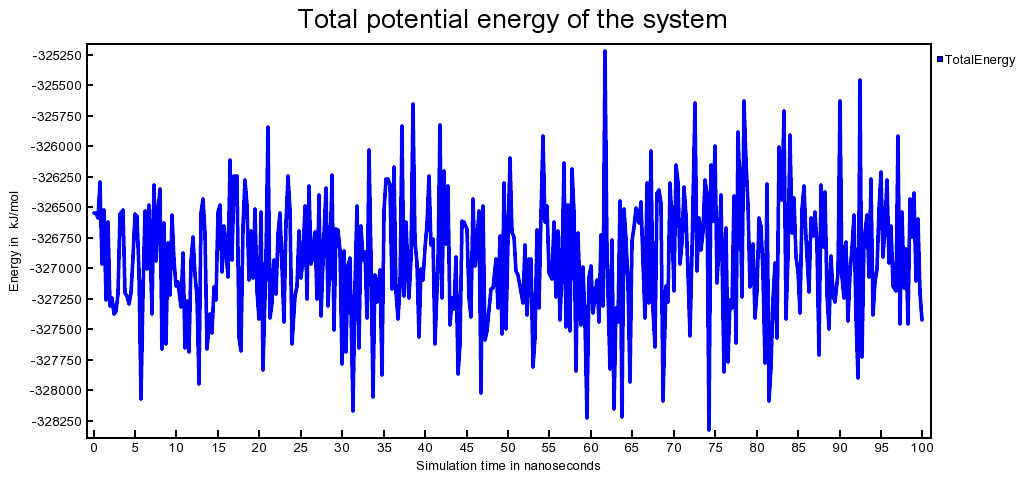

Supplement: S8 File — (ZIP) [file pone.0323003.s008.zip › Result/complex_2/complex_2_report_figure4.png]

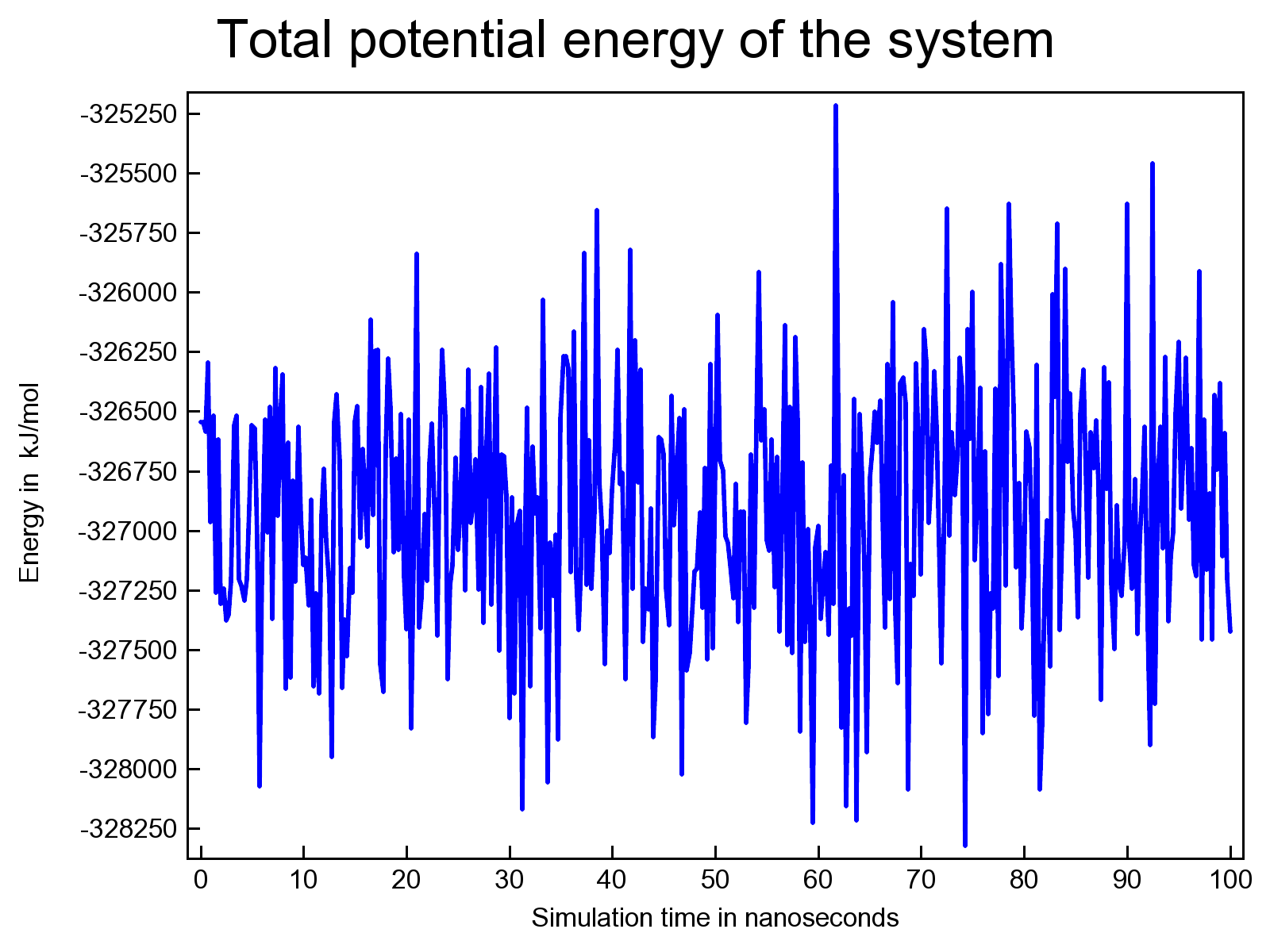

Supplement: S8 File — (ZIP) [file pone.0323003.s008.zip › Result/complex_2/complex_2_report_figure4_hires.png]

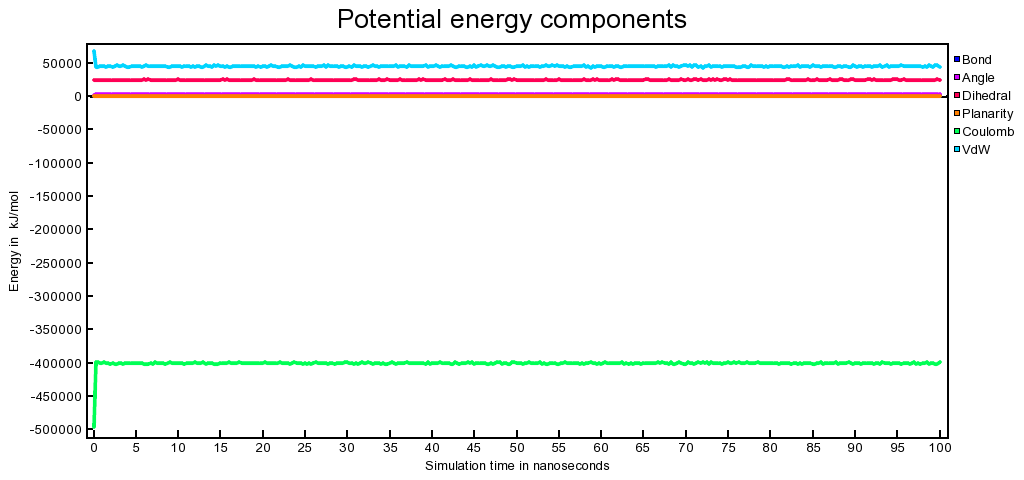

Supplement: S8 File — (ZIP) [file pone.0323003.s008.zip › Result/complex_2/complex_2_report_figure5.png]

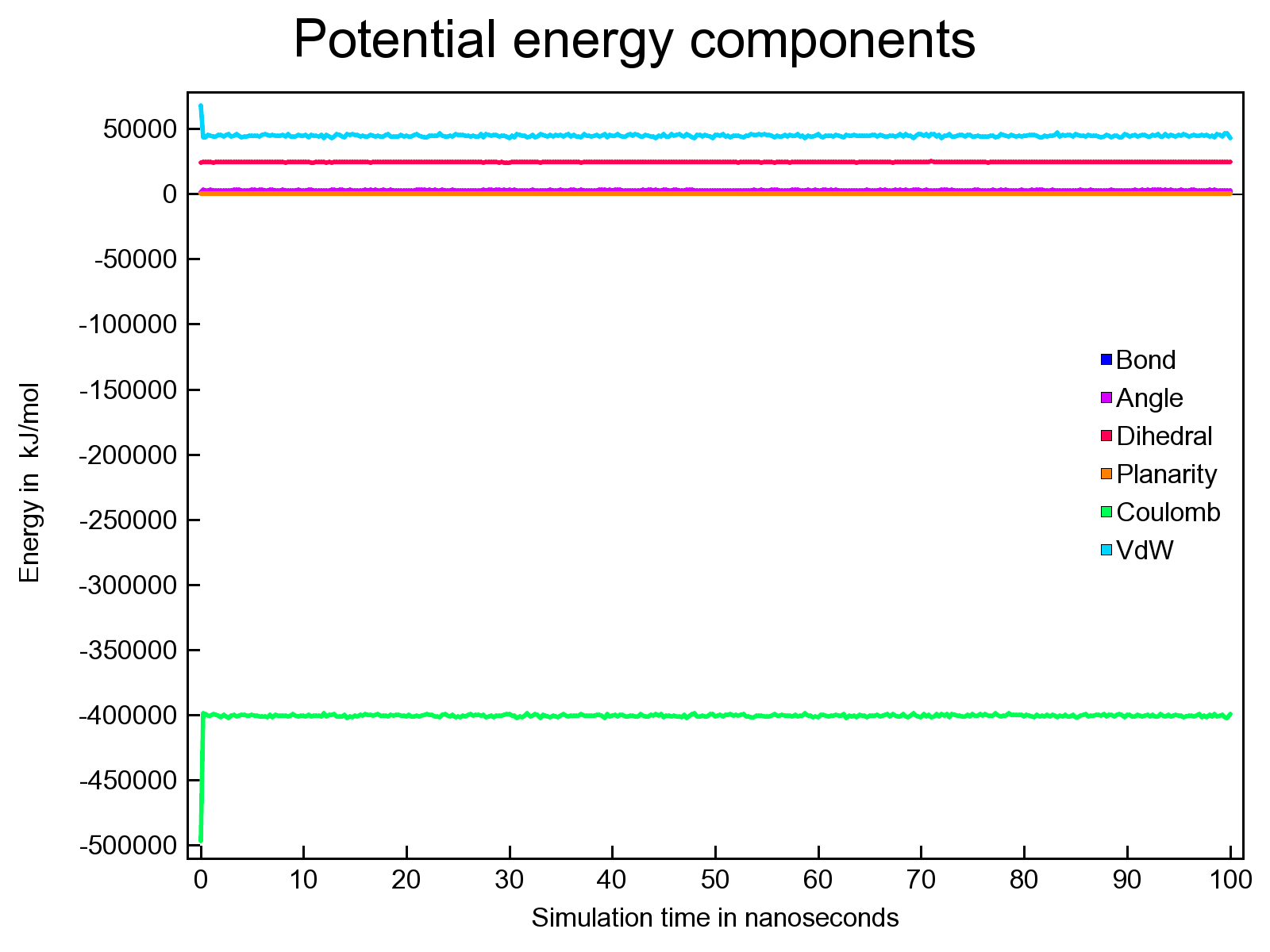

Supplement: S8 File — (ZIP) [file pone.0323003.s008.zip › Result/complex_2/complex_2_report_figure5_hires.png]

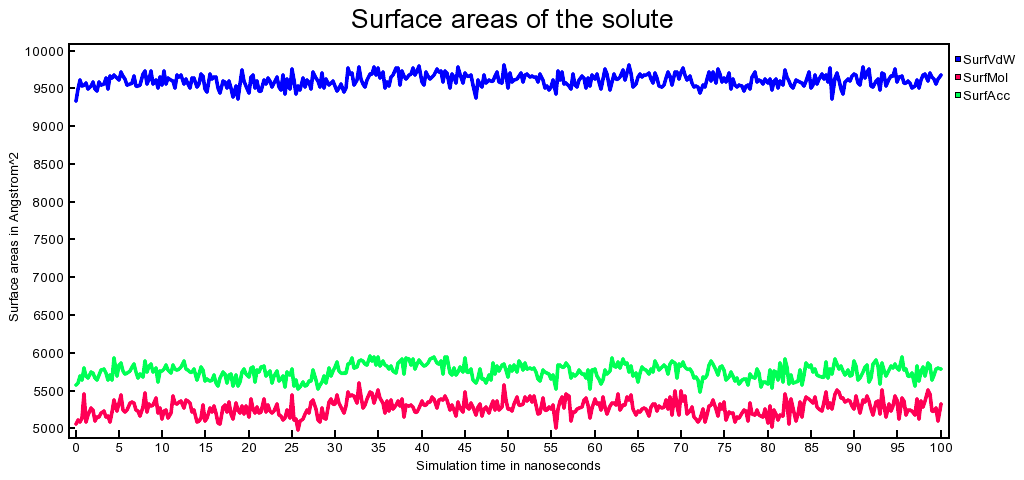

Supplement: S8 File — (ZIP) [file pone.0323003.s008.zip › Result/complex_2/complex_2_report_figure6.png]

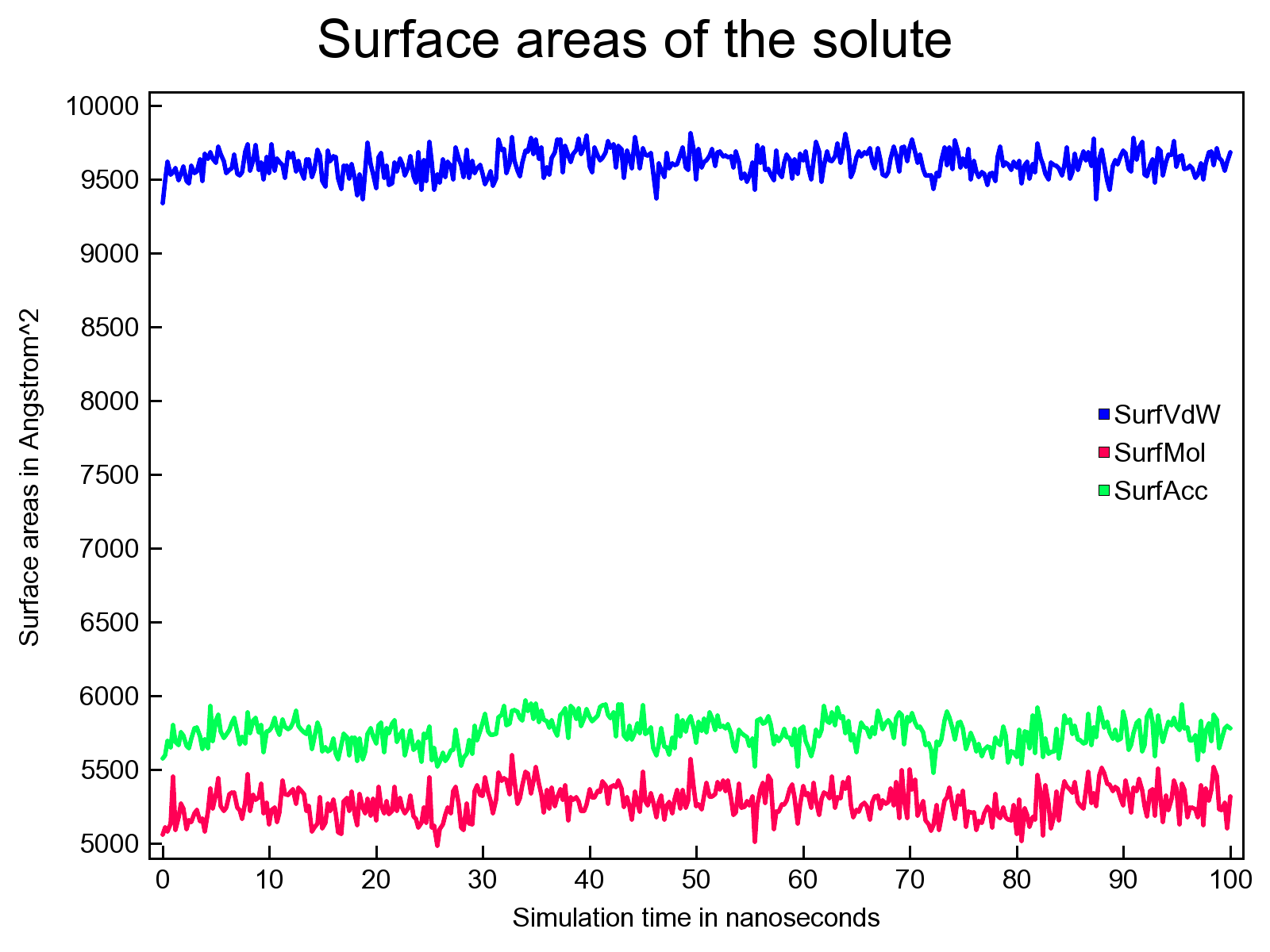

Supplement: S8 File — (ZIP) [file pone.0323003.s008.zip › Result/complex_2/complex_2_report_figure6_hires.png]

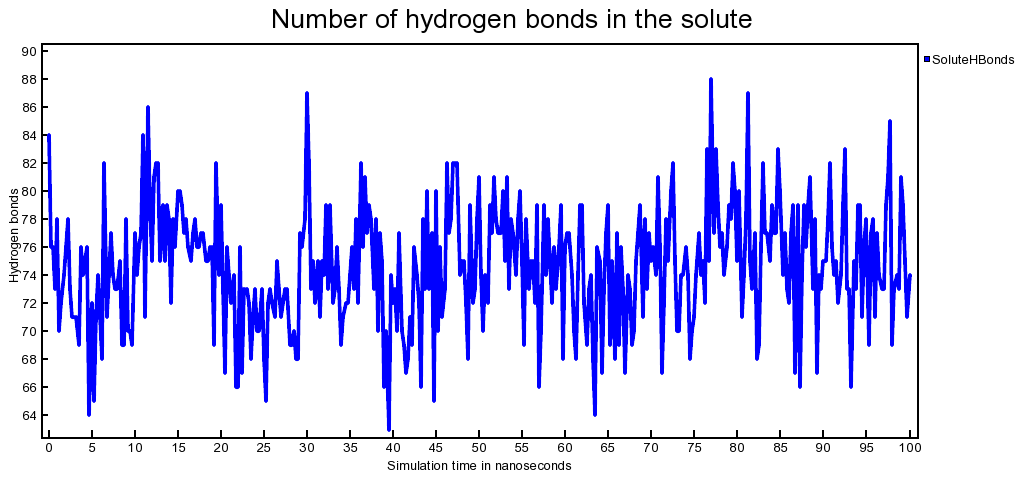

Supplement: S8 File — (ZIP) [file pone.0323003.s008.zip › Result/complex_2/complex_2_report_figure7.png]

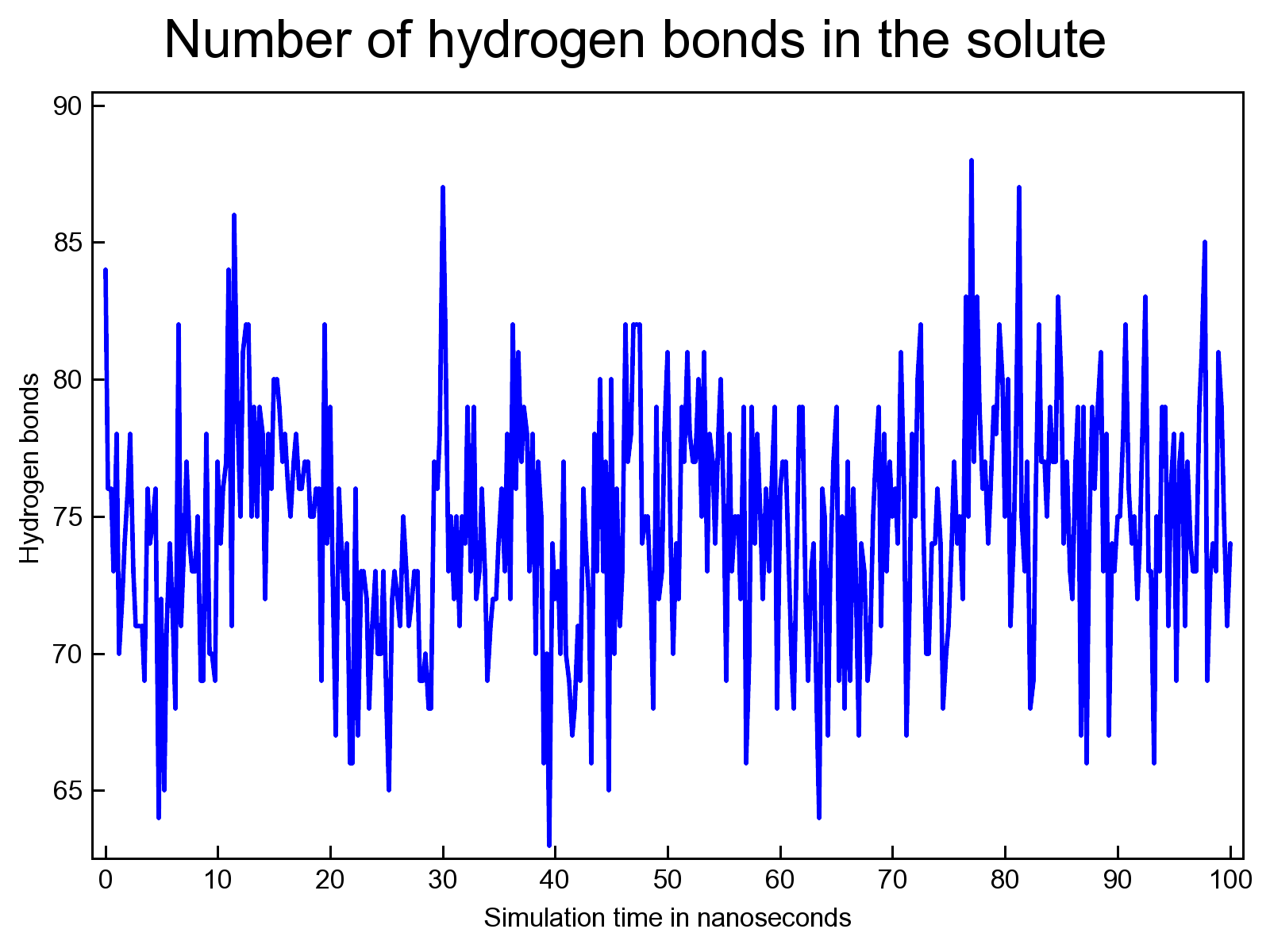

Supplement: S8 File — (ZIP) [file pone.0323003.s008.zip › Result/complex_2/complex_2_report_figure7_hires.png]

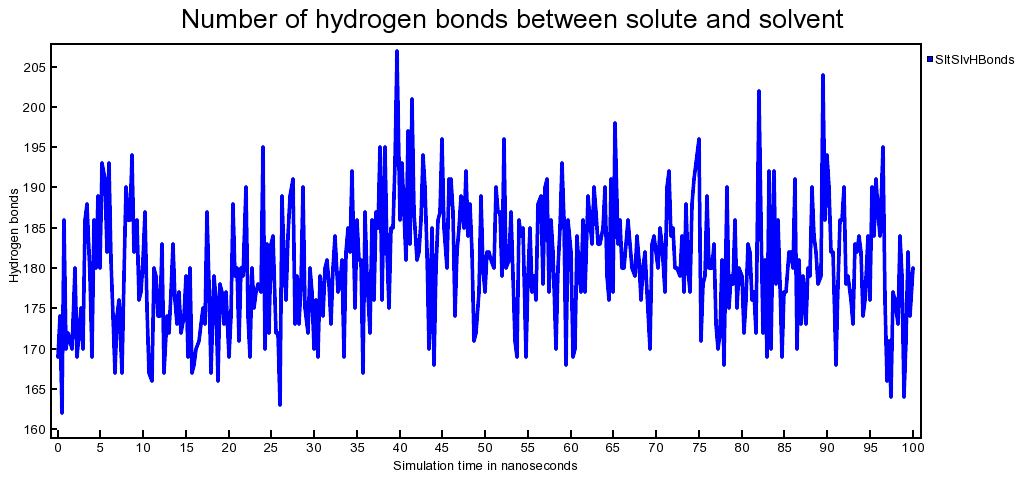

Supplement: S8 File — (ZIP) [file pone.0323003.s008.zip › Result/complex_2/complex_2_report_figure8.png]

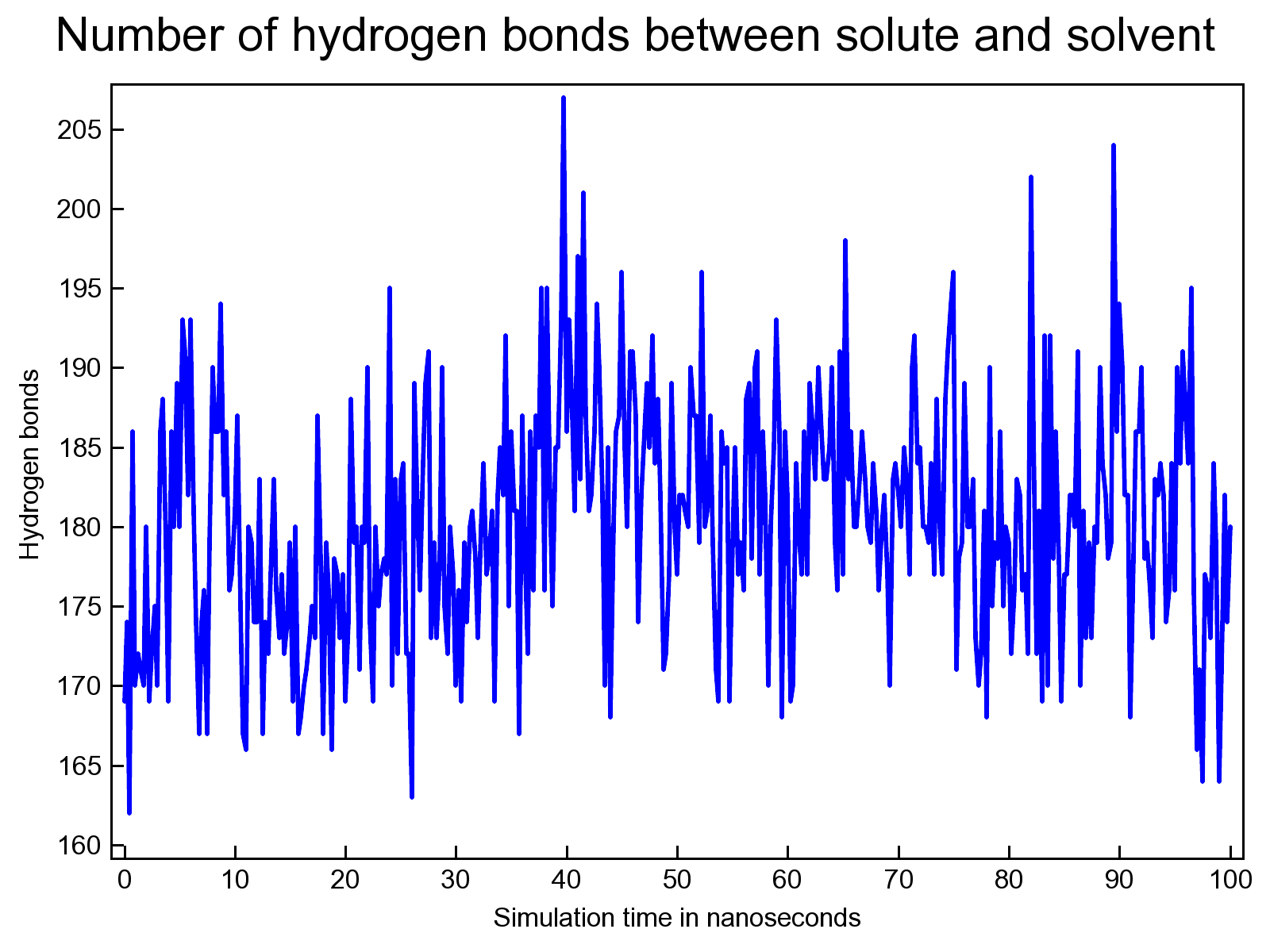

Supplement: S8 File — (ZIP) [file pone.0323003.s008.zip › Result/complex_2/complex_2_report_figure8_hires.png]

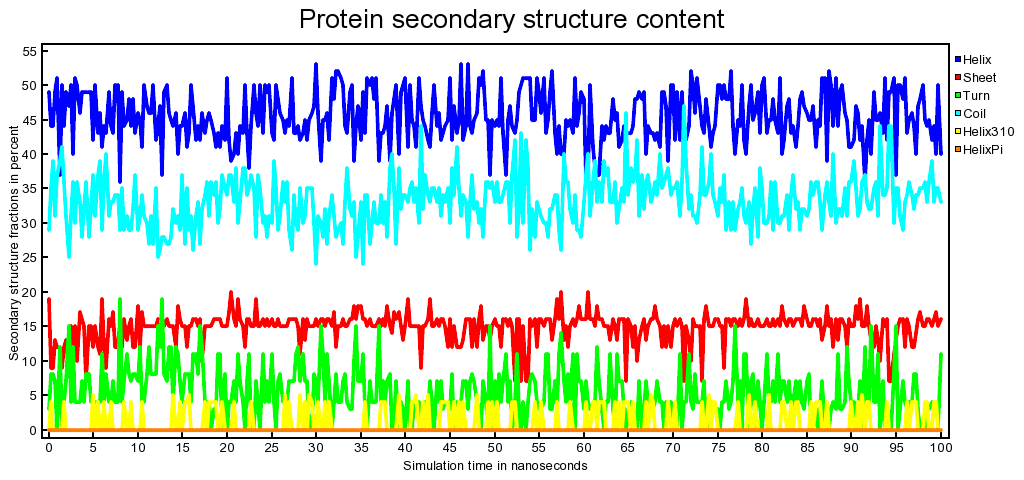

Supplement: S8 File — (ZIP) [file pone.0323003.s008.zip › Result/complex_2/complex_2_report_figure9.png]

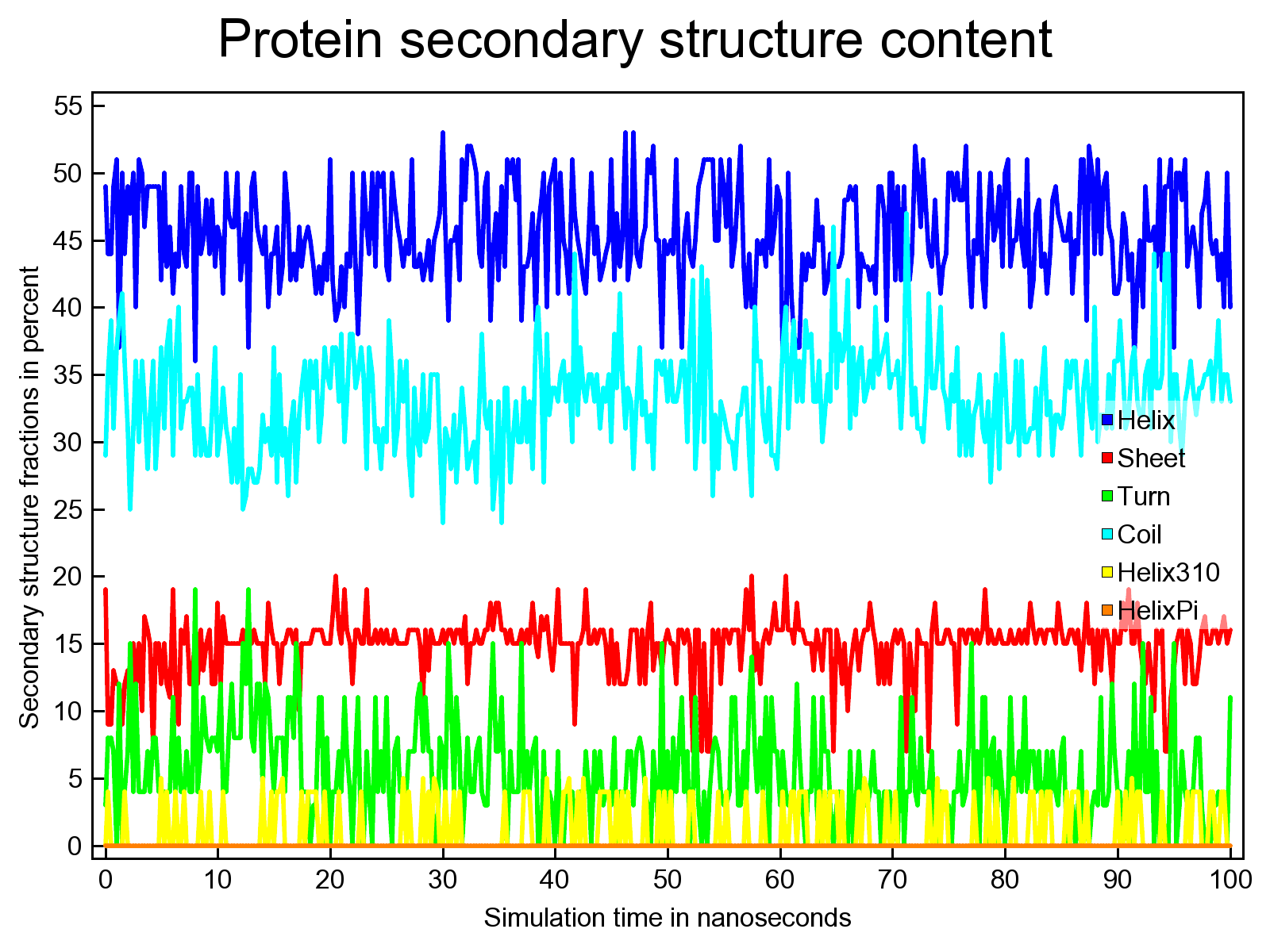

Supplement: S8 File — (ZIP) [file pone.0323003.s008.zip › Result/complex_2/complex_2_report_figure9_hires.png]
